# A transdiagnostic meta-analysis of acute augmentations to psychological therapy

**DOI:** 10.1038/s44220-023-00048-6

**Published:** 2023-05-15

**Authors:** Camilla L. Nord, Beth Longley, Quentin Dercon, Veronica Phillips, Julia Funk, Siobhan Gormley, Rachel Knight, Alicia J. Smith, Tim Dalgleish

**Affiliations:** 1 MRC Cognition and Brain Sciences Unit, University of Cambridge, Cambridge, UK; 2 Division of Psychology and Mental Health, University of Manchester, Manchester, UK; 3 Department of Psychiatry, University of Oxford, Oxford, UK; 4 Medical Library, University of Cambridge, Cambridge, UK; 5 Department of Psychology, LMU Munich, Munich, Germany; 6 Cambridgeshire and Peterborough National Health Service Foundation Trust, Cambridge, UK

**Keywords:** Psychology, Translational research

## Abstract

At least half of all patients with mental health disorders do not respond adequately to psychological therapy. Acutely enhancing particular biological or psychological processes during psychological therapy may improve treatment outcomes. However, previous studies are confined to specific augmentation approaches, typically assessed within single diagnostic categories. Our objective was to assess to what degree acute augmentations of psychological therapy reduce psychiatric symptoms and estimate effect sizes of augmentation types (for example, brain stimulation or psychedelics). We searched Medline, PsycINFO and Embase for controlled studies published between database inception and 25 May 2022. We conducted a preregistered random-effects meta-analysis (PROSPERO CRD42021236403). We identified 108 studies (*N* = 5,889). Acute augmentation significantly reduced the severity of mental health problems (Hedges’ *g* = −0.27, 95% CI: [−0.36, −0.18]; *P* < 0.0001), particularly for the transdiagnostic dimensions 'Fear' and 'Distress'. This result survived a trim-and-fill analysis to account for publication bias. Subgroup analyses revealed that pharmacological, psychological and somatic augmentations were effective, but to varying degrees. Acute augmentation approaches are a promising route to improve outcomes from psychological therapy.

## Abstract (web summary)

Nord and coauthors performed a meta-analysis to identify the degree to which specific biological, psychological or somatic interventions used as acute augmentation of psychological therapy further reduced psychiatric symptoms, demonstrating that various types of acute augmentations (such as pharmacological augmentations) were effective but to varying degrees.

## Main

Mental ill health is the leading cause of global disability^1^, with an estimated economic cost of nearly £119 billion in the United Kingdom alone^2^. Although treatment efficacy and availability for psychiatric disorders have improved over the past 30 years, population prevalence remains high^3^. Psychological therapy (also known as psychotherapy or talk therapy) confers widespread improvements in the disability, mortality and occupational health of patients with mental health conditions, including severe mental illness, particularly when combined with pharmacological treatment^4–8^. Yet even the best therapies leave substantial proportions of patients with ongoing clinical problems^3^.

To improve this, over the past two decades, acute augmentations of psychological therapy have gained traction in experimental neuroscience and psychology. Acute augmentations are interventions delivered before, during or after a session of psychological therapy, with the intention of enhancing the therapeutic impact of a single session of therapy (although acute augmentations can and often are repeated across multiple therapy sessions). This approach is distinct from long-term combination therapy, which describes two independently efficacious therapies that may (or may not) convey additive benefits when used in the same patients (such as daily antidepressant medication prescribed alongside a course of psychological therapy). By contrast, the rationale for acute augmentation is to enhance specific biological or psychological mechanisms of an individual psychological therapy session. For example, a pharmacological agent or cognitive training task might be administered to enhance the impact of a particular aspect of the therapy session, such as mental imagery^9^ or fear extinction^10^.

Acute augmentations often originate from basic experimental science. For instance, experimental neuroscience studies found the partial N-methyl-D-aspartate agonist D-cycloserine, administered before or shortly after exposure to a feared stimulus, enhanced fear extinction^10^. This discovery led to early-stage trials showing that D-cycloserine has a small but significant augmentative effect on the clinical efficacy of exposure-based cognitive behavior therapy for anxiety, obsessive–compulsive and posttraumatic stress disorders^11^. A similar translational process occurred in the psychological literature: influential models suggest that patients with affective disorders^12^ have disrupted processing of negatively valenced information. These cognitive biases are proposed to play a causal role in development and maintenance of various psychiatric disorders^13^. This led to trials testing cognitive bias modification as an acute enhancement of psychological therapy for social anxiety^14^, panic^15^ and obsessive–compulsive disorder^16^, among others. Separately, studies have examined the augmentative effects of somatic interventions such as non-invasive brain stimulation^17–23^, exercise^24^ and controlled breathing^25,26^ on psychological therapy.

While augmentation studies focus almost exclusively on single-diagnosis populations, these examples illustrate that similar or identical augmentations are often tested across multiple patient populations on the basis of similar theoretical grounding. As such, augmentation approaches are likely to have transdiagnostic mechanisms and utility. This echoes a general increasing recognition that psychological treatments and their underlying mechanisms transcend diagnostic boundaries^27^.

It is currently unknown whether augmentative interventions for psychological therapies are potentially useful across a range of disorders and augmentation approaches. To address this gap in the evidence, we conducted a meta-analysis across the extant literature to determine whether acute augmentation of manualized psychological therapy was generally effective for transdiagnostic psychiatric symptoms. Our goal was to quantify the effect sizes of augmentations of psychological therapies. We included a diverse range of acute augmentations, including medications, brain stimulation and cognitive training. This enabled us to assess the overall efficacy of acute augmentations of psychological therapy, as well as examine specific subcategories of augmentation.

## Results

### Results of initial and updated searches

**Fig. 1:**
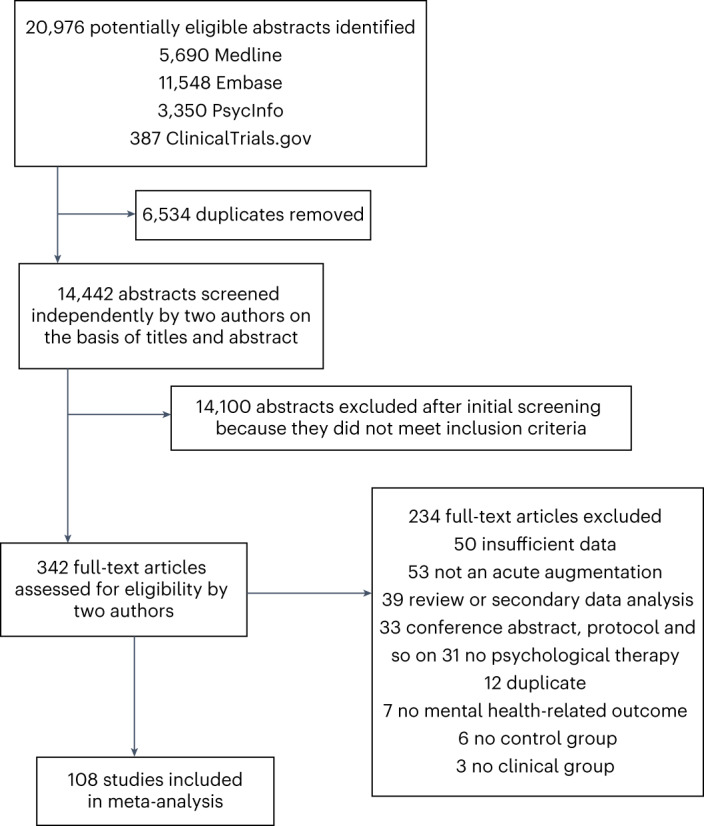
Flow chart of screening protocol. PRISMA flow diagram describing the process of study identification, de-duplication, and screening (note this occurred at two time-points due to the updated search in May 2022).

The initial search results (conducted February 2021) included 12,458 unique studies; the updated search (May 2022) identified a further 1,984 studies (Fig. 1 and Supplementary Tables 1 and 2). Two independent raters screened the titles and abstracts of all studies, after which 12,193 studies were excluded (initial search) followed by 1,907 studies being excluded (updated search) when they did not meet one or more of our prespecified criteria. Raters were 97.29% concordant on the initial (2021) search, with a kappa value (indicating proportion of agreement to include/exclude beyond that expected by chance) of 0.43 (moderate agreement), and 96.27% concordant on the updated (2022) search, with a kappa value of 0.58 for the updated searches, indicating moderate-to-good agreement. Note the large number of abstracts screened (>12,000) likely contributes to the discrepancy between very high concordance (>95%) and moderate (although significantly better than chance) kappa (~0.5) (ref. ^28^). Following discussion to resolve discrepancies, both raters independently screened the full text of 265 studies (initial search) followed by 77 studies (updated search) to assess whether they met inclusion criteria.

The primary reason for excluding studies during full-text screening was the absence of an acute augmentative intervention (53 studies). After all screening, 108 studies were included in the meta-analysis, representing 5,889 participants.

### Characteristics of included studies

The characteristics of each included study are listed in Supplementary Table 2. Of the 108 included studies, 59 involved pharmacological augmentations (2,381 patients), 26 had psychological or cognitive augmentations (2,442 patients) and 20 used somatic augmentations (951 patients). Three studies were included in the primary analysis but excluded from the subgroup analyses as they did not clearly represent any of the three subgroups. All studies were consistently categorized into one of these three groups (or excluded) by the two reviewers (100% concordance). Note that all augmentations tested in two or more studies included in the meta-analysis were ‘transdiagnostic’ (tested in at least two diagnostic/clinical categories) (Table 1).

**Table 1:** Augmentation types according to diagnostic/clinical category in which they were tested

Augmentation type	Clinical category
**Memory**	**MDD, phobia, PTSD**
**Imagery**	**PTSD, social anxiety**
**Cortisol**	**Phobia**
**D-cycloserine**	**OCD, PTSD, schizophrenia, social anxiety, SUD, panic disorder, phobia, body dysmorphic disorder**
**Brain stimulation**	**MDD, anxiety, phobia, SUD, PTSD**
**Psychotropic**	**PTSD, social anxiety, MDD, panic disorder, comorbid PTSD–SUD, anxiety, adjustment disorder**
**Oxytocin**	**Phobia, schizophrenia, PTSD, SUD**
**Yohimbine**	**Phobia, social anxiety, PTSD**
**Motivational**	**PTSD, OCD, anxiety, PTSD**
**Bias modifications**	**Panic disorder, OCD**
Exercise	Panic disorder, MDD
Breathing training	MDD, phobia
Animal-assisted	SUD, PTSD
Hypnosis	Acute stress, depression
Virtual reality	Binge-eating disorder, phobia
Hydrocortisone	Phobia, PTSD

All augmentations tested in at least two studies are listed (augmentations tested in three or more studies are indicated in bold to highlight their inclusion in the subgroup meta-analyses). MDD, major depressive disorder; OCD, obsessive–compulsive disorder; PTSD, posttraumatic stress disorder; SUD, substance-use disorder.

### Data synthesis

**Fig. 2:**
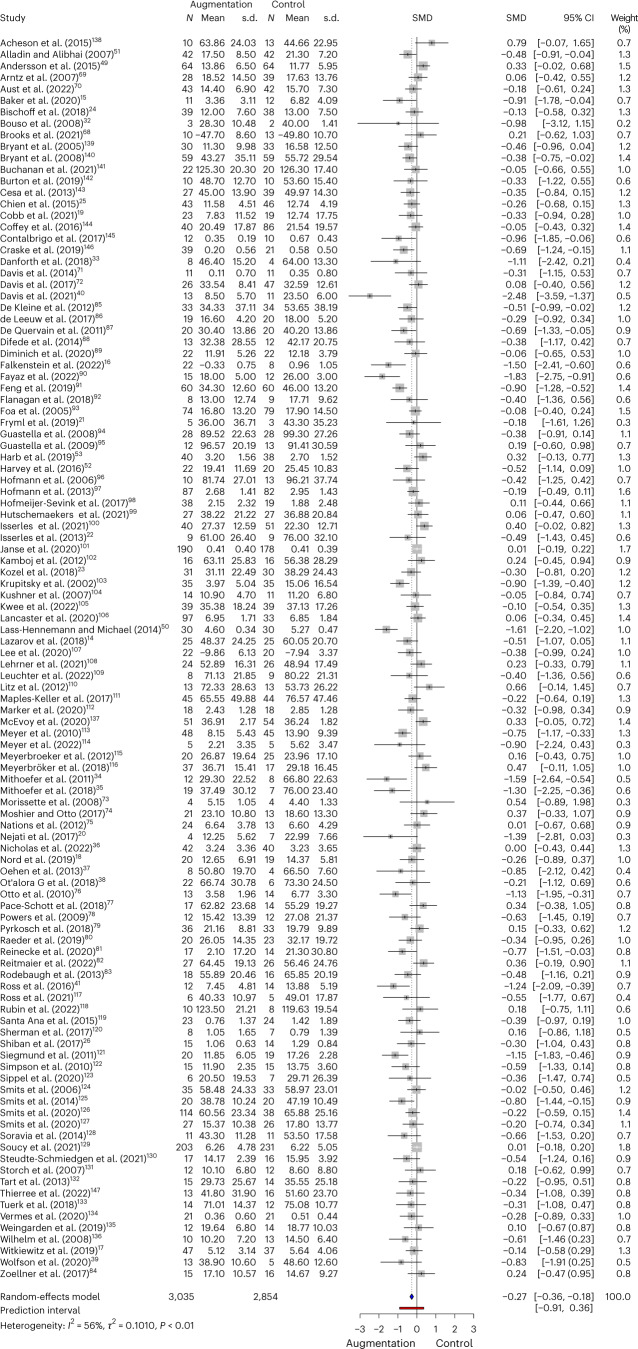
Synthesis of all studies included in the random-effects meta-analysis. The primary outcome of our random-effects meta-analysis of the included acute augmentation studies, i.e. the difference between the two groups at post-treatment (standardised mean difference: SMD) and the 95% confidence interval (CI) around the effect size. Data from refs. ^14–26,32–39,41,49–53,68–147^.

We found a significant advantage for augmentation groups over control groups with a small-to-moderate effect size (Hedges’ *g* = −0.27, 95% confidence interval (CI): [−0.36, −0.18]; *P* < 0.0001) in the full sample (*N* = 5,889) (Fig. 2).

**Fig. 3:**
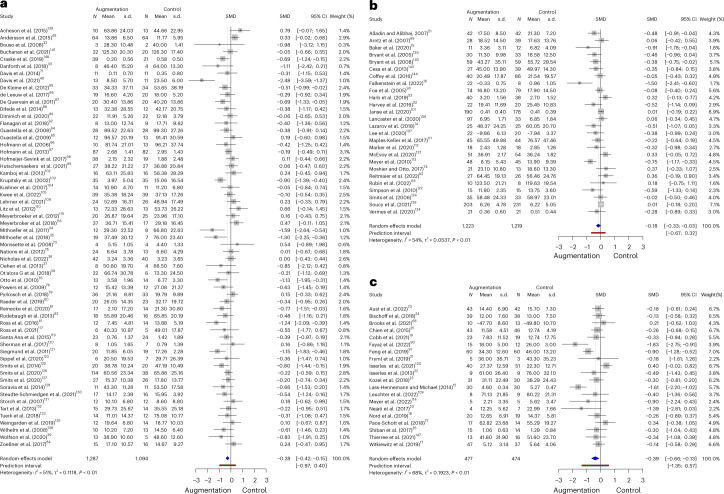
Forest plots of study subcategories in the random-effects meta-analysis. **a**, Pharmacological. **b**, Psychological. **c**, Somatic. Data from refs. ^14–26,32–39,41,49–53,68–147^.

Next, we conducted planned subgroup analyses, additionally reporting Bonferroni correction for multiple comparisons, to determine whether certain types of augmentations were more efficacious than others. All three subgroups (pharmacological, psychological and somatic) showed efficacy at *P* = 0.05, but only pharmacological and somatic interventions showed efficacy at our Bonferroni-corrected alpha (*P* = 0.016). Effect sizes also varied between augmentation subgroups: the effect size for trials using pharmacological augmentations alone (59 studies; *N* = 2,381) was comparable to the overall effect size (Hedges’ *g* = −0.28, 95% CI: [−0.42, −0.15]; *P* < 0.0001) (Fig. 3A). Studies using a psychological or cognitive augmentation (26 studies, *N* = 2,442) had a smaller effect-size estimate (Hedges’ *g* = −0.18, 95% CI: [−0.33, −0.027]; *P* = 0.0225) (Fig. 3B). Studies using a somatic augmentation (20 studies; *N* = 951) had a larger effect-size estimate but a much wider confidence interval (Hedges’ *g* = −0.39, 95% CI: [−0.66, −0.13]; *P* = 0.0063) (see Fig. 3C).

### Exploratory analyses

We conducted two sets of exploratory analyses to examine in more detail the relative efficacy of specific augmentation subtypes and transdiagnostic dimensions.

To examine the relative efficacy of specific augmentation subtypes, we divided the dataset into ten categories, representing finer-grained augmentation approaches. Only those categories with a minimum of three studies per approach were analyzed (*k* = 66 studies met this criterion; see Supplementary Information for included studies). In these preliminary analyses, there was a striking variation between effect sizes of different subtypes. Augmentations using psychedelics, 3,4-methylenedioxy methamphetamine (MDMA, or ecstasy) and cannabis (*k* = 13, labeled ‘psychotropic’ in the following) had a large significant effect (Hedges’ *g* = −0.84, 95% CI: [−1.26, −0.42]; *P* = 0.0009), which remained after exclusion of a wait-list-controlled study (Hedges’ *g* = −0.69, 95% CI: [−1.04, −0.34]; *P* = 0.001). D-cycloserine (*k* = 24) also had a significant, but small, effect size (Hedges’ *g* = −0.21, 95% CI: [−0.37, −0.037]; *P* = 0.019). Other subtypes had non-significant effects, which in some cases may due to low statistical power (for example, brain stimulation: (Hedges’ *g* = −0.28, 95% CI: [−0.6, 0.03]; *P* = 0.072)) (Supplementary Table 6).

We also classified each study into clinical categories using the Hierarchical Taxonomy of Psychopathology, a transdiagnostic approach for classifying psychiatric disorders^29^. According to this approach, transdiagnostic augmentations were efficacious for Fear and Distress dimensions (Fear: Hedges’ *g* = −0.26, 95% CI: [−0.40, −0.12]; *P* = 0.0004; Distress: Hedges’ *g* = −0.28, 95% CI: [−0.44, −0.13]; *P* = 0.0008, with ‘Fear’ encompassing traditional categories of phobias, panic and social anxiety and ‘Distress’ encompassing dysphoria, suicidality and generalized anxiety disorder, among others). Augmentation approaches were not effective for ‘Substance abuse’ or ‘Thought disorder’ dimensions, although these analyses were probably underpowered due to the substantially fewer studies represented (nine and three, respectively) (Substance abuse: Hedges’ *g* = −0.40, 95% CI: [−0.87, 0.065]; *P* = 0.083; Thought disorder: Hedges’ *g* = −0.11, 95% CI: [−0.41, 0.20]; *P* = 0.278) (Supplementary Information section 2.7).

### Heterogeneity and publication bias

To measure between-study heterogeneity, we calculated the *I*² statistic^30^. The *I*² showed moderate heterogeneity (*I*^2^ = 56.2%, 95% CI: [45.7%, 64.7%]; Cochran’s *Q* = 244.4, *P* < 0.0001) across all study subcategories: pharmacological (*I*^2^ = 50.0%, 95% CI: [33.7%, 63.6%]; Cochran’s *Q* = 118.1, *P* < 0.0001), psychological (*I*^2^ = 53.6%, 95% CI: [27.6%, 70.3%]; Cochran’s *Q* = 53.9, *P* = 0.0007) and somatic (*I*^2^ = 67.8%, 95% CI: [48.7%, 79.8%], Cochran’s *Q* = 59.0, *P* < 0.0001).

We conducted an exploratory follow-up analysis repeating our main analysis but excluding outliers, defined as having confidence intervals not overlapping with the confidence intervals of the pooled effect. Sixteen outliers were found (nine pharmacological, three psychological and four somatic: Supplementary Table 3). After exclusion of outliers, there was no longer significant between-study heterogeneity: *I*^2^ = 9.8% (95% CI: [0%; 31.1%]), Cochran’s *Q* = 100.9, *P* = 0.2243. Nevertheless, evidence for our main effect remained (Hedges’ *g* = −0.21, 95% CI: [−0.27; −0.14]; *P* < 0.0001).

**Fig. 4:**
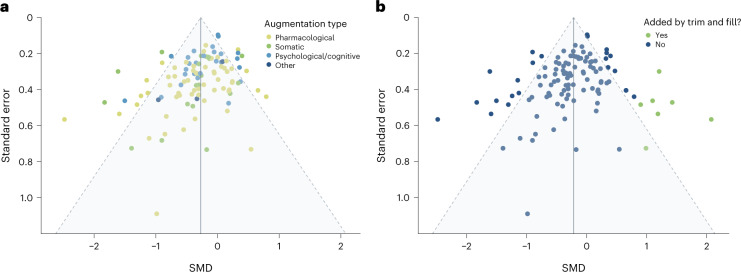
Funnel plot of all studies. **a**, To evaluate publication bias, we plotted the standardized mean difference of each study with respect to its standard error. We visually inspected the plot for asymmetry: 79.6% of studies fell within the funnel. **b**, We then ran a trim-and-fill analysis, which added seven studies.

Visual inspection of the funnel plot (Fig. 4a) showed some moderate asymmetry; 79.6% of studies fell within the funnel. Egger’s regression test confirmed asymmetry (*t*(106) = −4.02, *P* = 0.0001), which was unchanged by excluding outliers (Egger’s regression test *t*(90) = −4.00, *P* = 0.0001). A trim-and-fill analysis was then run, which added seven studies to the right-hand side of the funnel plot (Fig. 4b). Although this again slightly decreased the overall estimated effect size, evidence for our main effect remained strong (Hedges’ *g* = −0.21, 95% CI: [−0.32; −0.11]; *P* = 0.0001).

### Risk-of-bias analysis

We analyzed the risk of study bias using the Cochrane risk-of-bias tool. We assessed the presence of random sequence generation, concealment of allocation, blinding of participants and treatment providers, blinding of outcome assessment and incomplete outcome data, indicating whether each of these criteria was present, absent or uncertain (Supplementary Table 4). Note the presence of some degree of risk of bias across most studies.

To assess the contribution of risk of bias overall, we repeated our primary analysis excluding studies without certain blinded outcome assessment, which we determined to be the most critical risk of experimenter bias in acute augmentations of psychotherapy trials. We found 15 studies without, or with uncertain, blinding of outcome assessment.

Excluding these ‘high-risk’ studies and analyzing only ‘lower-risk’ studies did not alter our main effect, supporting the efficacy of brief augmentations (Hedges’ *g* = −0.29, 95% CI: [−0.39, −0.19]; *P* < 0.0001) (Supplementary Fig. 3). Analyzing only high-risk studies, by contrast, did not support the efficacy of brief augmentations (Hedges’ *g* = −0.19, 95% CI: [−0.45, 0.07]; *P* = 0.013) (Supplementary Fig. 4). This suggests this bias was not a major driver of our effect.

### Sensitivity analyses

We conducted a number of sensitivity analyses to assess the robustness of our effect. First, we replicated our effect when excluding the small number (*k* = 2) of non-randomized trials (Hedges’ *g* = −0.27, 95% CI: [−0.36, −0.17]; *P* < 0.0001) (Supplementary Fig. 7) (the two non-randomized studies themselves did not replicate the effect (Hedges’ *g* = −0.64, 95% CI = [−4.62, −0.17]; *P* < 0.0001) and studies without an active therapeutic control (wait list; *k* = 2) (Hedges’ *g* = −0.26, 95% CI: [−0.35, −0.17]; *P* < 0.0001).

We performed a further sensitivity analysis, excluding six studies where (although meeting our own inclusion criteria) the study authors framed the study as more combinatory than augmentative in nature. This did not alter our main analysis results (Hedges’ *g* = −0.28, 95% CI: [−0.37, −0.18]; *P* < 0.0001) (Supplementary Fig. 6). However, the remaining 20 psychological augmentation studies showed a reduced and now non-significant effect of augmentation: Hedges’ *g* = −0.16, 95% CI: [− 0.35, 0.03]; *P* = 0.089 (Supplementary Fig. 5).

## Discussion

We conducted a transdiagnostic meta-analysis to estimate the effect size of acutely augmented psychological therapy, compared with a control or non-augmented therapy. On our preregistered primary outcome, we found that acute augmentations of psychological therapy are efficacious for a diverse array of mental health problems, with an overall small effect size. Examining specific transdiagnostic dimensions and intervention types, we found support for the use of acute augmentations for fear- and distress-based mental health problems and for interventions employing acute pharmacological (for example, the psychedelic compound psilocybin) and somatic (for example, transcranial magnetic stimulation) augmentations of psychological therapy. This suggests that augmenting specific therapy sessions with medication or brain stimulation interventions (among others) could improve clinical response to psychological therapy.

Although we found evidence for overall moderate efficacy of acute augmentations, we also reported substantial heterogeneity among all types of studies. This did not seem to be driving our overall effect: excluding a small number of outliers (*k* = 15), heterogeneity decreased substantially while the effect of acute augmentations remained with a near-equal magnitude. Similarly, a number of sensitivity analyses did not substantially alter our finding, suggesting that our overall effect was not driven by poor outcome blinding and lack of randomization, which affected a small number of studies.

The small effect size we report is comparable to the effect size recently found in a large umbrella review for the combination of psychotherapy and pharmacotherapy, compared with either as a monotherapy^31^. This is compelling because the nature of long-term (compared with brief) augmentation is very different, commonly associated with different disorders (major depression versus phobias), and far fewer administrations of a medication take place in the context of brief pharmacological augmentations than during longer-term combination therapies. Potentially, the lower intensity and chronicity of brief interventions may offer a similar benefit with fewer adverse effects and at reduced cost; this is an important topic for investigation in future work.

An additional implication of our results is that certain brief augmentations may confer much larger effects than either other brief augmentations or, indeed, long-term combination of psychotherapy and pharmacotherapy. Our meta-analysis included three broad types of augmentation trials: psychological, pharmacological and somatic. In exploratory analyses, we found that all three acute augmentations were somewhat efficacious, but to varying degrees. We found particularly robust evidence for pharmacological augmentations, which also comprised the largest group of acute augmentations. This is exemplified in the case of *d*-cycloserine augmentations of psychological therapy, which represented the largest single augmentation type in our meta-analysis and had a similarly small effect size to our overall effect. Previous meta-analyses have reported a small advantage of d-cycloserine-enhanced exposure therapy, albeit with notable between-study variation^11^, a conclusion supported by our meta-analysis.

Our meta-analysis also included a number of more atypical acute pharmacological augmentations, such as eight studies testing MDMA (ecstasy)^32–39^ or psilocybin^40,41^ as therapeutic augmentations, an area of substantial current interest in the psychiatric research community^42–44^. We found evidence for a large significant effect of recreational drugs as therapeutic enhancers (*d* = −0.84, comprising MDMA, psilocybin and cannabis augmentations) in a subanalysis of 13 such studies (*N* = 412). This compares favorably with the significant but smaller effects of *d*-cycloserine (*d* = 0.21). Our meta-analysis suggests that psychedelic and related compounds may be particularly effective brief therapeutic enhancers.

By contrast, we found weaker evidence for various psychological augmentations (*d* = −0.18), the efficacy of which was not as robust as the other groups of interventions; this did not survive analyses of specific augmentation types (memory, imagery, motivation or bias training) or exclusion of studies framed as more ‘combinatory’ than augmentative. While somatic augmentations had the largest effect size of the three, the estimate of this effect was less precise (95% CI: [−0.66, −0.13]. This is probably a reflection of this category’s relatively smaller size and/or greater heterogeneity among the augmentations, which included brain stimulation, sleep, time of day, exercise and controlled breathing. We had sufficient studies to perform an exploratory subanalysis on the somatic subcategory of brain stimulation, which showed a significant effect (smaller than psychedelic and related drugs but of a similar magnitude to the overall effect of augmentations (*d* = 0.28)). In future, precise estimates of other somatic augmentations’ efficacy could be obtained by splitting this category into its constituent parts (requiring more studies with particular somatic augmentation approaches).

Recent efforts have been made to characterize the ‘active ingredients’ of mental health treatment: that is, the aspects of an intervention that drive clinical effects^45^. This approach may hold particular potential for uncovering acute augmentation strategies. By first identifying key therapeutic mechanisms of action via reverse translation, acute therapeutic augmentations can be applied in novel combinations to specifically target those mechanisms. For instance, mental imagery is thought be an ‘active ingredient’ of effective psychological therapy^46^. Techniques designed to enhance mental imagery have been widely studied as acute augmentations of psychological therapies, with some evidence of success, particularly in young people^46^. Outside of a research setting, imagery-enhanced group cognitive behavior therapy is a highly effective intervention, even when delivered by trainee clinicians in independent settings^9^. Future work could establish whether imagery-based psychological augmentation could be particularly helpful in certain patients or at certain points in a longer course of therapy. Unlike chronic, long-term combination therapies, acute augmentations support modular therapy: during a course of therapy, d-cycloserine might be used in one session dedicated to exposure; brain stimulation might be used at a later session focusing on cognitive restructuring.

Acute augmentations of psychological therapy hold particular promise as tests of whether particular approaches can be subject to clinical translation. For example, as with previous psychological augmentation approaches such as imagery, acute pharmacological augmentations could also focus on targeting specific active ingredients of psychological therapy. This strategy could be helpful for individuals unlikely to respond to psychological therapy alone. For example, patients with pathological disgust respond generally poorly to exposure therapy; our recent experimental work suggests that a peripherally selective dopamine antagonist may enhance disgust habituation, an active ingredient of exposure therapy^47^. Alternatively or in addition, specific neural effects of psychological therapy might be used as future targets of augmentation, for example, with brain stimulation interventions.

A limitation of our meta-analysis is its potential for bias due to existing issues with scientific robustness in the clinical literature. This was seen both in the risk-of-bias assessment, where most studies showed some degree of bias, and in the funnel plot of the effect-size estimates of individual studies against their standard errors, where we found some evidence of asymmetry in our sample of studies. In the absence of publication bias (or any other sources of heterogeneity biased by sample size), 95% of studies should fall inside the funnel of the plot^48^; in our results, approximately 80% of studies fall within the funnel. This probably indicates some publication bias in the augmentation literature, potentially due to selective outcome reporting or under-reporting of null results. This could have inflated our estimates of effect size. However, asymmetry in funnel plots can also be caused by sources of heterogeneity that are genuinely associated with sample size, such as smaller studies having higher treatment fidelity or larger studies generally delivering less-intense interventions due to feasibility^48^, which are plausible in the context of the acute augmentation literature. In our sample, both publication bias and true sources of heterogeneity may have contributed to our findings. Crucially, our central results survived correction for publication bias with trim and fill.

A second caveat originates from the transdiagnostic approach employed in this meta-analysis. A risk of this approach is that it obscures the effectiveness or ineffectiveness of augmentations for particular subcategories of symptoms, such as eating-disorder symptoms, which were under-represented in our studies. In partial mitigation of this concern, trials measuring these symptoms were not statistical outliers in our analysis. As only two studies measured eating-disorder-related symptoms as a primary outcome in our sample, these studies would have been excluded from diagnosis-specific meta-analysis. Therefore, we believe it was beneficial to use a transdiagnostic approach, despite the potential risk that our findings may differ between subcategories in the future. It is possible that some groups of symptoms may be particularly sensitive (or insensitive) to augmentative approaches, which will require a larger number of trials in each domain in the future.

One putative limitation is whether augmented psychological therapy involves additional overall contact time due to the duration of the augmentation, compared with standard interventions. For the majority of pharmacological and somatic augmentations, this was not the case: almost all used placebo medication (for example, refs. ^32^^,^^49^), sham brain stimulation (for example, ref. ^21^) or other somatic conditions (for example, refs. ^24^^,^^50^)—control groups with equivalent therapeutic contact time and duration. By contrast, several psychological augmentations involved additional contact time or duration, such as 15 minutes of hypnotic induction preceding cognitive behavioral therapy^51^ (others, including cognitive bias modification^16^, memory support^52^ and imagery rescripting^53^, explicitly equalized therapist contact time/duration between groups). However, evidence for psychological augmentations was the weakest of the three, suggesting that any increased contact time or duration was unlikely to drive the overall efficacy of augmentative approaches.

Given the debilitating effects of mental health disorders and the challenges in their treatment, improving mental health treatment is of the utmost economic and societal importance. For many decades, research has tended to conclude that psychological and pharmacological treatments are comparably efficacious, or that even different psychological therapies are equally effective (the ‘dodo-bird verdict’: that everybody has won and must have prizes^54^). This is despite differential treatment mechanisms within and between therapeutic modalities^55,56^. Improving on our current treatment paradigm may involve a paradigm shift towards individualized, mechanism-focused interventions. One approach to this is acute augmentations designed to optimize the specific subcomponents of therapy indicated for that particular patient. Augmentations of psychological therapy represent an area in which translational science can be directly tested, potentially improving clinical treatment rapidly and at scale. Future studies should investigate optimal combinations of augmentations and therapies, going beyond use of a single intervention to enhance a course of therapy and towards matching specific medications to the activities and contents of specific therapy sessions.

Additional augmentative domains could also be examined, for example augmentations focused on potentiating the therapeutic relationship or alliance^57^. Even our most effective psychiatric interventions leave many patients with clinically-significant problems. To move the needle on mental health interventions, we may need a different approach. Acute augmentations of psychological therapy represent a route from experimental science to clinical translation and may offer particular promise for precision psychiatry approaches. Our meta-analysis supports the usefulness of acute augmentations in the context of mostly smaller, experimental trials, but there remains a need for robust, real-world trials testing acute augmentation strategies.

## Methods

### Preregistration

Our meta-analysis was preregistered on PROSPERO (CRD42021236403). There were no deviations from the preregistered methods. We report both preregistered (primary) analyses and exploratory (secondary) analyses.

### Inclusion criteria

We included studies in which the following were true.

- Participants had a diagnosed mental disorder^58^ according to a validated questionnaire or interview assessment, or presented with subthreshold clinical symptoms, and in which the mean age of the sample was over 18 years. Mental disorders included mood disorders, anxiety disorders, eating disorders, personality disorders, psychotic disorders, trauma and stressor-related disorders, obsessive–compulsive disorder and substance-use disorders. Patients with comorbidities were not excluded.
- Treatment involved manualized psychological therapy (for example, cognitive behavioral therapy) combined with an acute augmentation (for example, exercise) administered before, during or after the therapy with the aim of enhancing the effect of the psychological therapy on mental health problems. Psychological therapy could have taken place face to face or online but was required to have been at least partially clinician led (not self-guided). Manualized therapies are those for which a manual or guide exists (for example, cognitive behavioral therapy for depression) and where the specific manual used is cited by the study authors.
- A control or comparison group for the acute augmentation was included, namely, a placebo drug, treatment as usual, another psychological treatment, wait list, sham brain stimulation or sham cognitive training. We recorded the primary mental health-related outcome reported in the study (for example, interview, self-report questionnaire or physiological measure). Included studies could be randomized or non-randomized controlled trials, including feasibility trials. Studies were required to include at least one session of psychological therapy and one acute augmentation aimed at enhancing the effects of the psychological therapy.

### Exclusion criteria

We excluded studies aimed at treating neurological disorders or neurodevelopmental disorders unless the primary outcome of the intervention was a mental health symptom. We also excluded studies in which the intervention was a longer-term combination treatment, the study did not report any mental health-related outcomes measured on a continuous scale, the study was a case report, case series, conference abstract or animal study or the data presented were insufficient to calculate effect sizes.

### Information sources and search strategy

We conducted searches in the following databases: Medline (via Ovid), PsycINFO (via EBSCOhost) and Embase (via Ovid) (from inception until 2 February 2021; following a reviewer request to repeat searches, from inception until 25 May 2022). The full search terms used on each database are provided in Supplementary Information. We used a search strategy combining psychological therapy terms with augmentation-related terms, trial terms and psychiatric disorder-related terms using Boolean operators (>100 search terms included per database). Clinical trials registrations were also searched via ClinicalTrials.gov with the search terms (psychiatric disorder*) AND (behavioral OR pharmacological OR somatic) AND (psychological therapy).

We also performed reference tracing for additional studies meeting inclusion criteria referenced in the articles from our database searches.

To determine study suitability, we used a two-step approach. First, a minimum of two independent raters separately screened all titles and abstracts. Studies were excluded if they did not meet inclusion criteria on the basis of their title or abstract. Any discrepancies were resolved via discussion. Subsequently, the full text of the included studies was assessed by two independent raters to ensure these met inclusion criteria; discrepancies were again resolved by discussion.

The following information was collected from the included studies: author(s), year of publication, mental health assessment measure, mental health diagnosis or dimension studied, sample size, mean age, gender, types of interventions (augmentation(s) and control(s)), duration of interventions and outcome data (see the following).

### Primary outcome data

We extracted summary data from reports by recording the primary mental health-related outcome reported in each study. If no measure was designated primary, or the primary outcome was categorical, we recorded the first continuous mental health-related outcome reported in the Results. If no continuous outcomes were reported, we excluded the study.

### Grouping for synthesis

Our preregistered primary outcome was the standardized mean difference (Hedges’ *g*) across all studies corresponding to the difference between the two groups at posttreatment (standardized mean difference) and the 95% confidence intervals around the effect sizes. We conducted random-effects meta-analysis due to anticipated between-study heterogeneity (confirmed in heterogeneity analyses (Results)).

We ran exploratory subgroup analyses using the same approach to obtain the effect size for each of three types of augmentations: (1) pharmacological (for example, cortisol or psilocybin), (2) psychological or cognitive (for example, attention bias modification or cognitive control training) and (3) somatic (nonpharmacological biological interventions, for example, transcranial magnetic stimulation or exercise).

### Synthesis of results

In most studies, a lower value posttreatment indicated an improvement in mental health. In three studies, a higher value on their primary outcome indicated an improvement in symptoms, so mean values were multiplied by −1 to align the direction of the scales. Where we could not retrieve the relevant summary statistics from the text, the software WebPlotDigitizer was used to extract the data from figures where possible^59^ (WebPlotDigitizer is accurate to the level of an individual pixel of the plot published; users can zoom in and out to select the most precise location within a pixel for data extraction. Intercoder reliability and validity of WebPlotDigitizer is high (*r* = 0.999 intercoder correlation)^60^). In studies that included multiple augmentation groups that all met criteria, the sample size, means and standard deviations for the multiple groups were combined, as per Cochrane recommendations^61^; we followed the same recommendation for studies reporting multiple active control groups (combining and treating as a single control group); for studies reporting active and inactive control groups, we always used the active control.

All analyses were performed in the statistical software environment R using the meta package^62^. We report pooled between-group effects by calculating Hedges’ *g* corresponding to the difference between the augmentation group and the control group posttreatment (standardized mean difference)^63^. We also report the 95% confidence intervals around the effect sizes as a measure of certainty in the evidence for each pooled outcome. We used a random-effects model to calculate a pooled effect size and the Der Simonian–Laird method to estimate tau squared^64^.

We used the *I*² statistic to assess the proportion of variability due to heterogeneity and calculated the *Q* statistic to test the existence of heterogeneity in our sample^30^. We interpreted the *I*² value according to the following guidelines: 25% representing low heterogeneity, 50% representing moderate heterogeneity and 75% representing high heterogeneity^30^. We also assessed the potential for publication bias via (1) visual inspection of a funnel plot and (2) Egger’s regression test^65^, and conducted a trim-and-fill analysis to assess the impact of including studies that may be missing due to publication bias^66^.

### Risk of bias

For each included study, we assessed risk of bias according to the Cochrane risk-of-bias tool^67^. This tool provides a framework for assessing different contributors to potential study bias originating from study design, conduct, analysis and reporting^67^. For each study, we assessed the use of random sequence generation, concealment of allocation, blinding of participants and treatment providers, blinding of outcome assessment and incomplete outcome data. We summarized each study’s overall risk of bias following the Cochrane recommendation to use the domain(s) of most importance in the context of our meta-analysis^67^. We assessed blinding of outcome assessment to be the most critical risk in acute augmentations of psychotherapy trials. Therefore, we repeated our primary analysis excluding studies without blinded outcome assessment or with uncertain blinding of outcome assessment.

### Reporting summary

Further information on research design is available in the Nature Portfolio Reporting Summary linked to this article.

### Supplementary information

Supplementary Information Supplementary Figs. 1–10, Tables 1–5 and Methods.

Reporting Summary

## Data Availability

The preregistration can be found at https://www.crd.york.ac.uk/prospero/display_record.php?ID=CRD42021236403, and all data can be found on the Open Science Framework: https://osf.io/a7x8j/.

## Code availability

All relevant analysis code can also be found on the Open Science Framework: https://osf.io/a7x8j/.
