# Supplementary material for: A transdiagnostic meta-analysis of acute augmentations to psychological therapy
Source: Nat Ment Health. 2023 May 15;1(6):389–401. doi: 10.1038/s44220-023-00048-6 (PMC11041792; doi:10.1038/s44220-023-00048-6)
Supplement: Supplementary file 1 — Supplementary Figs. 1–10, Tables 1–5 and Methods. [file 44220_2023_48_MOESM1_ESM.pdf]

nature mental health

Analysis

https://doi.org/10.1038/s44220-023-00048-6

A transdiagnostic meta-analysis of acute augmentations to psychological therapy

In the format provided by the authors and unedited

Supplemental Materials

1. Supplementary Methods

1.1. Search terms

The databases Medline (via Ovid), Embase (via Ovid) and PsycInfo (via Ebscohost) were searched from inception to February 2nd 2021 (initial search) and then again from inception until 25 May 2022 (updated search), using the search terms below. Results were limited to English language papers. In addition, the ClinicalTrials.gov website was searched for grey literature, using the modified search terms below.

Medline via Ovid

("cognitive processing" OR "narrative exposure therapy" OR "prolonged exposure therapy" OR "virtual reality exposure therapy" OR "cognitive behavioural therapy" OR "cognitive behavioral therapy" OR CBT OR "eye movement desensiti*" OR EMDR OR "psychodynamic psychotherapy" OR "interpersonal psychotherapy" OR "mindfulness" OR "acceptance and commitment therapy" OR "attention training" OR "dialectical behavioural therapy" OR "dialectical behavioral therapy" OR "animal assisted" OR "animal-assisted" OR "cognitive analytical therapy" OR "imagery psychotherapy" OR psychotherapy OR "Cognitive therapy" OR "behavioural activation" OR "behavioral activation" OR "behaviour therapy" OR "behavior therapy" OR "cognitive analytic therapy" OR "cognitive behaviour therapy" OR "cognitive behavior therapy" OR "dialectical behaviour therapy" OR "dialectical behavior therapy" OR ACT OR "exposure therapy").ti,ab,kw. OR Cognitive Behavioral Therapy/ or Narrative Therapy/ OR Eye Movement Desensitization Reprocessing/ OR psychotherapy/ or imagery, psychotherapy/ or interpersonal psychotherapy/ or psychotherapy, psychodynamic/ OR mindfulness/ OR "Acceptance and Commitment Therapy"/ OR animal assisted therapy/ OR behavior therapy/ OR dialectical behavior therapy/

AND

(psychiatric OR "mood disorder*" OR depress* OR dysphori* OR dysthymi* OR bipolar OR mania OR manic OR schiz* OR psychosis OR psychotic OR delusional OR paranoid OR anxiety OR "post traumatic stress disorder" OR PTSD OR "posttraumatic stress disorder*" OR "post-traumatic stress disorder*" OR "acute stress disorder*" OR agoraphobia OR phobia OR panic OR "obsessive compulsive" OR OCD OR hoarding OR "eating disorder*" OR anorexi* OR bulimi* OR "binge eating" OR "body dysmorphi*" OR "personality disorder*" OR borderline OR antisocial OR narcissistic OR histrionic OR somatoform OR somatic OR "substance use disorder*" OR "substance abuse" OR "substance dependence" OR addiction OR dependen* OR "oppositional defiant" OR "intermittent explosive" OR "sexual dysfunction" OR "dissociative disorder" OR Hypomania).ti,ab,kw. OR exp mental disorders/

AND

(augment* OR enhanc* OR adjunct* OR conjunct* OR amplify OR assist* OR supplement* OR combin*).ti,ab,kw.

AND

("clinical trial*" OR "controlled trial*" OR "Controlled Before-After Studies" OR "Pilot Projects" OR "feasibility trial*" OR "exploratory trial*").ti,ab,kw. OR exp Clinical Trials/ OR Controlled Before-After Studies/ OR Pilot Projects/

Embase via Ovid

("cognitive processing" OR "narrative exposure therapy" OR "prolonged exposure therapy" OR "virtual reality exposure therapy" OR "cognitive behavioural therapy" OR "cognitive behavioral therapy" OR CBT OR "eye movement desensiti*" OR EMDR OR "psychodynamic psychotherapy" OR "interpersonal psychotherapy" OR mindfulness OR "acceptance and commitment therapy" OR "attention training" OR "dialectical behavioural therapy" OR "dialectical behavioral therapy" OR "animal assisted" OR "animal-assisted" OR "cognitive analytical therapy" OR "imagery psychotherapy" OR psychotherapy OR “Cognitive therapy” OR “behavioural activation” OR “behavioral activation” OR “behaviour therapy” OR “behavior therapy” OR “cognitive analytic therapy” OR “cognitive behaviour therapy” OR “cognitive behavior therapy” OR “dialectical behaviour therapy” OR “dialectical behavior therapy” OR ACT OR “exposure therapy”).ti,ab,kw. OR Cognitive Behavioral Therapy/ or Narrative Therapy/ OR Eye Movement Desensitization Reprocessing/ OR psychotherapy/ or imagery, psychotherapy/ or interpersonal psychotherapy/ or psychotherapy, psychodynamic/ OR mindfulness/ OR "Acceptance and Commitment Therapy"/ OR animal assisted therapy/ OR cognitive therapy/ OR behaviour therapy/ OR exposure therapy/

AND

(psychiatric OR "mood disorder*" OR depress* OR dysphori* OR dysthymi* OR bipolar OR mania OR manic OR schiz* OR psychosis OR psychotic OR delusional OR paranoid OR anxiety OR "post traumatic stress disorder" OR PTSD OR "posttraumatic stress disorder*" OR "post-traumatic stress disorder*" OR "acute stress disorder*" OR agoraphobia OR phobia OR panic OR "obsessive compulsive" OR OCD OR hoarding OR "eating disorder*" OR anorexi* OR bulimi* OR "binge eating" OR "body dysmorphi*" OR "personality disorder*" OR borderline OR antisocial OR narcissistic OR histrionic OR somatoform OR somatic OR "substance use disorder*" OR "substance abuse" OR "substance dependence" OR addiction OR dependen* OR "oppositional defiant" OR "intermittent explosive" OR "sexual dysfunction" OR "dissociative disorder" OR Hypomania).ti,ab,kw. OR exp mental disorders/

AND

(augment* OR enhanc* OR adjunct* OR conjunct* OR amplify OR assist* OR supplement* OR combin*).ti,ab,kw.

AND

("clinical trial*" OR "controlled trial*" OR "Controlled Before-After Studies" OR "Pilot Projects" OR “feasibility trial*” OR “exploratory trial*”).ti,ab,kw. OR exp clinical trial/ OR Controlled Before-After Studies/ OR pilot projects/

PsycInfo via Ebscohost

TI (“cognitive processing” OR “narrative exposure therapy” OR “prolonged exposure therapy” OR "virtual reality exposure therapy" OR “cognitive behavioural therapy” OR

“cognitive behavioral therapy” OR CBT OR “eye movement desensiti*” OR EMDR OR “psychodynamic psychotherapy” OR “interpersonal psychotherapy” OR “mindfulness” OR “acceptance and commitment therapy” OR “attention training” OR “dialectical behavioural therapy” OR “dialectical behavioral therapy” OR “animal assisted” OR “animal-assisted” OR “cognitive analytical therapy” OR “imagery psychotherapy” OR “psychotherapy” OR “Cognitive therapy” OR “behavioural activation” OR “behavioral activation” OR “behaviour therapy” OR “behavior therapy” OR “cognitive analytic therapy” OR “cognitive behaviour therapy” OR “cognitive behavior therapy” OR “dialectical behaviour therapy” OR “dialectical behavior therapy” OR ACT OR “exposure therapy”) OR AB (“cognitive processing” OR “narrative exposure therapy” OR “prolonged exposure therapy” OR "virtual reality exposure therapy” OR “cognitive behavioural therapy” OR “cognitive behavioral therapy” OR CBT OR “eye movement desensiti*” OR EMDR OR “psychodynamic psychotherapy” OR “interpersonal psychotherapy” OR “mindfulness” OR “acceptance and commitment therapy” OR “attention training” OR “dialectical behavioural therapy” OR “dialectical behavioral therapy” OR “animal assisted” OR “animal-assisted” OR “cognitive analytical therapy” OR “imagery psychotherapy” OR “psychotherapy” OR “Cognitive therapy” OR “behavioural activation” OR “behavioral activation” OR “behaviour therapy” OR “behavior therapy” OR “cognitive analytic therapy” OR “cognitive behaviour therapy” OR “cognitive behavior therapy” OR “dialectical behaviour therapy” OR “dialectical behavior therapy” OR ACT OR “exposure therapy”) OR DE "Cognitive Processing Therapy" OR DE "Narrative Therapy" OR DE "Exposure Therapy" OR DE "Eye Movement Desensitization Therapy" OR DE "Psychodynamic Psychotherapy" OR DE "Interpersonal Psychotherapy" OR DE "Mindfulness" OR DE "Animal Assisted Therapy" OR DE "Psychotherapy" OR DE "Cognitive Therapy" OR DE "Behavioral Activation System" OR DE "Behavior Therapy" OR DE "Cognitive Behavior Therapy" OR DE "Dialectical Behavior Therapy" OR DE "Exposure Therapy"

AND

TI (psychiatric OR "mood disorder*" OR depress* OR dysphori* OR dysthymi* OR bipolar OR mania OR manic OR schiz* OR psychosis OR psychotic OR delusional OR paranoid OR anxiety OR "post traumatic stress disorder" OR PTSD OR "posttraumatic stress disorder*" OR "post-traumatic stress disorder*" OR "acute stress disorder*" OR agoraphobia OR phobia OR panic OR "obsessive compulsive" OR OCD OR hoarding OR "eating disorder*" OR anorexi* OR bulimi* OR "binge eating" OR "body dysmorphi*" OR "personality disorder*" OR borderline OR antisocial OR narcissistic OR histrionic OR somatoform OR somatic OR "substance use disorder*" OR "substance abuse" OR "substance dependence" OR addiction OR dependen* OR "oppositional defiant" OR "intermittent explosive" OR "sexual dysfunction" OR “dissociative disorder” OR Hypomania) OR AB (psychiatric OR "mood disorder*" OR depress* OR dysphori* OR dysthymi* OR bipolar OR mania OR manic OR schiz* OR psychosis OR psychotic OR delusional OR paranoid OR anxiety OR "post traumatic stress disorder" OR PTSD OR "posttraumatic stress disorder*" OR "post-traumatic stress disorder*" OR "acute stress disorder*" OR agoraphobia OR phobia OR panic OR "obsessive compulsive" OR OCD OR hoarding OR "eating disorder*" OR anorexi* OR bulimi* OR "binge eating" OR "body dysmorphi*" OR "personality disorder*" OR borderline OR antisocial OR narcissistic OR histrionic OR somatoform OR somatic OR "substance use disorder*" OR "substance abuse" OR "substance dependence" OR addiction OR dependen* OR "oppositional defiant" OR

"intermittent explosive" OR "sexual dysfunction" OR “dissociative disorder” OR Hypomania) OR DE "Mental Disorders" OR DE "Affective Disorders" OR DE "Anxiety Disorders" OR DE "Autism Spectrum Disorders" OR DE "Bipolar Disorder" OR DE "Borderline States" OR DE "Chronic Mental Illness" OR DE "Dissociative Disorders" OR DE "Eating Disorders" OR DE "Gender Dysphoria" OR DE "Mental Disorders due to General Medical Conditions" OR DE "Neurocognitive Disorders" OR DE "Neurodevelopmental Disorders" OR DE "Neurosis" OR DE "Paraphilias" OR DE "Personality Disorders" OR DE "Psychosis" OR DE "Serious Mental Illness" OR DE "Sleep Wake Disorders" OR DE "Somatoform Disorders" OR DE "Stress and Trauma Related Disorders" OR DE "Substance Related and Addictive Disorders" OR DE "Thought Disturbances" OR DE “Hypomania”

AND

TI (augment* OR enhanc* OR adjunct* OR conjunct* OR amplify OR assist* OR supplement* OR combin*) OR AB (augment* OR enhanc* OR adjunct* OR conjunct* OR amplify OR assist* OR supplement* OR combin*)

AND

TI (“clinical trial*” OR “controlled trial*” OR "Controlled Before-After Studies" OR "Pilot Projects" OR “feasibility trial*” OR “exploratory trial*”) OR AB (“clinical trial*” OR “controlled trial*” OR "Controlled Before-After Studies" OR "Pilot Projects" OR “feasibility trial*” OR “exploratory trial*”) OR DE "Clinical Trials" OR DE "Randomized Controlled Trials"

ClinicalTrials.gov

(Psychiatric disorder*) AND (behavioral OR pharmacological OR somatic) AND (psychological therapy)

2. Supplementary Results

2.1. Database search results

Supplemental Table S1. Studies identified by database in each search

Database	Results	New results (updated May 2022)
Medline via Ovid	4923	767
Embase via Ovid	10175	1373
PsycInfo via Ebscohost	2908	443
ClinicalTrials.gov	341	46

Total results prior to deduplication: 17347
Unique results after deduplication: 12458
New total results May 2022: 2629
Unique results after deduplication May 2022: 1984

Supplemental Table S2. Characteristics of studies included in the meta-analysis

Authors (year of publication)	Mental health measure (assessment)	HiTOP dimension	N	Mean (SD) age (1) Augmentation (2) Control group	Gender (% female)	(1) Augmentation group(s) & (2) Control group	Type of augmentation	Duration
Acheson et al. (2015)[38]	Arachnophobia (FSQ)	Fear	23	(1) 33.3 (4.01)<br>(2) 26.77 (2.67)	82.6	(1) Exposure therapy + Oxytocin<br>(2) Exposure therapy + Placebo	Pharmacological	Single session
Alladin & Alibhai (2007)[39]	Depression (BDI-II)	Distress	84	(1) 35.67 (11.40)<br>(2) 33.67 (9.87)	57.1	(1) CBT + Hypnosis<br>(2) CBT	Psychological/cognitive	16 weeks
Andersson et al. (2015)[40]	OCD (Y-BOCS)	Fear	128	(1) 34.64 (12.42)<br>(2) 34.88 (12.22)	58	(1) ICBT + D-cycloserine<br>(2) ICBT + Placebo	Pharmacological	12 weeks
Arntz et al. (2007)[41]	PTSD (PSS-SR)	Distress	67	(1) 35.29 (11.29)<br>(2) 35.41 (12.73)	65.7	(1) Imaginal exposure + Imagery rescripting<br>(2) Imaginal exposure	Psychological/cognitive	10 weeks
Aust et al. (2022)[42]	Depression (MADRS)	Distress	95	(1) 42.7 (12.7)<br>(2) 39.0 (14.8)	60.1	(1) CBT + tDCS<br>(2) CBT + Sham tDCS	Somatic	6 weeks
Baker et al. (2020)[15]	Panic Disorder (PDSS)	Fear	23	*32.4 (1.53)	43.5	(1) CBT + Attention Bias Modification Task<br>(2) CBT + Sham task	Psychological/cognitive	6 weeks
Bischoff et al. (2018)[24]	Panic Disorder with agoraphobia (HAM-A)	Fear	77	(1) 37.2 (10)<br>(2) 36.2 (10.1)	63.6	(1) CBT + Moderate-intensity exercise<br>(2) CBT + Low-intensity exercise	Somatic	7 weeks
Bouso et al. (2008)[43]	PTSD (SSSPTSD)	Distress	5	(1) 36.67<br>(2) 33	100	(1) Psychotherapy + MDMA<br>(2) Psychotherapy + Placebo	Pharmacological	Not reported
Brooks et al. (2021)[44]	Depression or anxiety + cognitive decline (FCC)	-	26	(1) 68.2 (5.9)<br>(2) 69.2 (4.6)	46.2	(1) Mindfulness-based stress reduction + tDCS<br>(2) Mindfulness-based stress reduction + Sham	Somatic	10 weeks
Bryant et al. (2005)[45]	Acute stress disorder (IES)	Distress	63	(1) 32.97 (7.70)<br>(2) 33.09 (12.45)	Not reported	(1) CBT + Hypnosis<br>(2) CBT	Psychological/cognitive	5 weeks
Bryant et al. (2008)[46]	PTSD (CAPS)	Distress	118	(1) 34.85 (10.99)<br>(2) 39.98 (13.36)	Not reported	(1) Exposure therapy + IE/IVE & IE/IVE/CR<br>(2) Exposure therapy + IE & IVE	Psychological/cognitive	8 weeks
Buchanan et al. (2021)[47]	Social functioning in schizophrenia (BSFS)	Thought disorder	62	(1) 42.8 (8.7)<br>(2) 40.7 (10.2)	38.7	(1) CBSST + Oxytocin<br>(2) CBSST + Placebo	Pharmacological	24 weeks
Burton et al. (2019)[48]	PTSD (PCL-M)	Distress	20	(1) 48 (15)<br>(2) 46 (13)	20	(1) Psychotherapy + Equine-assistance<br>(2) Psychotherapy	Other	6 weeks
Cesa et al. (2013)[49]	Binge eating disorder (BSS)	-	66	(1) 32.9 (8.8)<br>(2) 31.02 (7.22)	100	(1) CBT + VR-enhanced CBT<br>(2) CBT	Psychological/cognitive	6 weeks
Chien et al. (2015)[25]	Depression (PSQI)	Distress	89	(1) 28.15 (10.66)<br>(2) 28.95 (11.55)	5.6	(1) Cognitive behavioural intervention + Breathing relaxation exercise<br>(2) No therapy or augmentation	Somatic	4 weeks
Cobb et al. (2021)[19]	Phobia (BAT)	Fear	49	*21.41 (7.12)	71	(1) Exposure therapy + tDCS<br>(2) Exposure therapy + Sham tDCS	Somatic	Single session
Coffey et al. (2016)[50]	PTSD (IES-R)	Distress	126	(1) 34.4 (11.32)<br>(2) 33.84 (10.44)	43.5	(1) Prolonged exposure + Motivational enhancement therapy<br>(2) Prolonged exposure + Relaxation session	Psychological/cognitive	5 to 8 weeks
Contalbrigo et al. (2017)[51]	Substance use disorder (SCL-90-R)	Substance abuse	22	(1) 35.5 (13.83)<br>(2) 42.9 (9.1)	0	(1) Psychotherapy + Dog assistance<br>(2) Psychotherapy	Other	6 months
Craske et al. (2019)[52]	Social anxiety (SCR to public speaking onset)	Fear	60	(1) 25.99 (10.27)<br>(2) 23.05 (8.58)	67.5	(1) Exposure therapy + Scopolamine (0.5mg)<br>(2) Exposure therapy + Placebo	Pharmacological	Not reported
Danforth et al. (2018)[53]	Social anxiety (LSAS)	Fear	12	(1) 32.8 (10.4)<br>(2) 28.3 (3.8)	16.7	(1) Psychotherapy + MDMA<br>(2) Psychotherapy + Placebo	Pharmacological	Not reported

Davis et al. (2014)[54]	Schizophrenia (Social-cognition composite score)	Thought disorder	22	(1) 42.8 (9.1)<br>(2) 37 (10.8)	0	(1) Social cognitive skills training + Oxytocin<br>(2) Social cognitive skills training + Placebo	Pharmacological	6 weeks
Davis et al. (2017)[55]	Social anxiety (LSAS)	Fear	73	(1) 26.88 (6.99)<br>(2) 24.98 (7.73)	68	(1) Exposure therapy + Power posing<br>(2) Exposure therapy + Submissive posing & no posing	Other	1 week
Davis et al. (2021)[56]	MDD (GRID-HAMD)	Distress	24	(1) 43.6 (13)<br>(2) 35.2 (9.9)	66.7	(1) Psychotherapy + Psilocybin<br>(2) Wait list (no therapy or augmentation)	Pharmacological	8 weeks
De Kleine et al. (2012)[57]	PTSD (CAPS)	Distress	67	(1) 36.27 (11.56)<br>(2) 40.26 (11.05)	80.6	(1) Prolonged exposure + D-cycloserine<br>(2) Prolonged exposure + Placebo	Pharmacological	Not reported
de Leeuw et al. (2017)[58]	OCD (Y-BOCS)	Fear	39	(1) 38.1 (14.2)<br>(2) 32.2 (8.9)	Not reported	(1) Exposure therapy + D-cycloserine<br>(2) Exposure therapy + Placebo	Pharmacological	7 weeks
De Quervain et al. (2011)[59]	Acrophobia (AQ)	Fear	40	(1) 42.8 (2.4)<br>(2) 40.2 (2.6)	45	(1) VRET + Cortisol<br>(2) VRET + Placebo	Pharmacological	2 weeks
Difede et al. (2014)[60]	PTSD (CAPS)	Distress	25	(1) 47.77 (11.92)<br>(2) 43.75 (8.72)	24	(1) VRET + D-cycloserine<br>(2) VRET + Placebo	Pharmacological	12 weeks
Diminich et al. (2020)[61]	Delusions (PSYRATS-D)	Thought disorder	58	(1) 44.60 (13.3)<br>(2) 45.55 (12.0)	39.7	(1) CBT + D-cycloserine<br>(2) CBT + Placebo	Pharmacological	12 weeks
Falkenstein et al. (2022)[16]	OCRDs (TBI, interpretation)	Fear	31	(1) 30.36 (8.44)<br>(2) 27.25 (3.77)	50	(1) IRT + CBM-I<br>(2) IRT + Psychoeducation	Psychological/cognitive	~4 weeks
Fayaz Feyzi et al. (2022)[62]	Methamphetamine use disorder (OCDUS)	Substance abuse	27	(1) 34 (6.7)<br>(2) 37 (7)	0	(1) Matrix Model psychotherapy + tDCS<br>(2) Matrix Model psychotherapy + Sham tDCS	Somatic	12 weeks
Feng et al. (2019)[63]	PTSD (CAPS)	Distress	120	(1) 40.9 (12.7)<br>(2) 41 (12.8)	69.2	(1) CBT + Transcutaneous electrical acupoint stimulation<br>(2) CBT + Simulated TEAS	Somatic	12 weeks
Flanagan et al. (2018)[64]	PTSD (CAPS)	Distress	17	(1) 41.63 (14.58)<br>(2) 45.78 (15.09)	17.7	(1) Prolonged exposure + Oxytocin<br>(2) Prolonged exposure + Placebo	Pharmacological	10 weeks
Foa et al. (2005)[65]	PTSD (PSS-I)	Distress	153	Not reported	100	(1) Prolonged exposure + Cognitive restructuring<br>(2) Prolonged exposure	Psychological/cognitive	12 weeks
Fryml et al. (2019)[21]	PTSD (CAPS)	Distress	8	(1) 27 (2.1)<br>(2) 30 (2.6)	12.5	(1) Exposure therapy + rTMS<br>(2) Exposure therapy + Sham rTMS	Somatic	5 weeks
Guastella et al. (2008)[66]	Social anxiety (SPAI)	Fear	56	*35.48 (11.35)	43	(1) Exposure therapy + D-cycloserine<br>(2) Exposure therapy + Placebo	Pharmacological	5 weeks
Guastella et al. (2009)[67]	Social anxiety (SPAI)	Fear	25	*42.28 (11.27)	0	(1) Exposure therapy + Oxytocin<br>(2) Exposure therapy + Placebo	Pharmacological	5 weeks
Harb et al. (2019)[68]	PTSD (NFQ)	Distress	78	(1) 37 (9.9)<br>(2) 37.2 (10.1)	13.9	(1) CBT-I + Imagery rescripting<br>(2) CBT-I	Psychological/cognitive	6 weeks
Harvey et al. (2016)[69]	MDD (IDS-SR)	Distress	42	(1) 43.92 (9.98)<br>(2) 44.65 (12.17)	60.4	(1) Cognitive therapy + Memory support<br>(2) Cognitive therapy	Psychological/cognitive	14 weeks
Hofmann et al. (2006)[70]	Social anxiety (SPAI)	Fear	23	(1) 30.08 (7.56)<br>(2) 36.53 (22.04)	33.3	(1) Exposure therapy + D-cycloserine<br>(2) Exposure therapy + Placebo	Pharmacological	5 weeks
Hofmann et al. (2013)[71]	Social anxiety (SPD-S)	Fear	169	(1) 34.6<br>(2) 30.5	43.2	(1) CBT + D-cycloserine<br>(2) CBT + Placebo	Pharmacological	12 weeks
Hofmeijer-Sevink et al. (2017)[72]	Panic disorder with agoraphobia (Mobility Inventory, alone subscale)	Fear	57	(1) 33.95 (10)<br>(2) 38.3 (11.4)	59.6	(1) Exposure with response prevention + D-cycloserine<br>(2) Exposure with response prevention + Placebo	Pharmacological	12 weeks
Hutschemaekers et al. (2021)[73]	Social anxiety disorder (SUDS)	Fear	54	(1) 22.61 (4.12)<br>(2) 24.00 (6.85)	100	(1) Exposure therapy + Testosterone<br>(2) Exposure therapy + Placebo	Pharmacological	Twice over month

Isserles et al. (2021)[74]	PTSD (CAPS)	Distress	125	(1) 44.8 (13.19)<br>(2) 43.7 (12.25)	66.4	(1) Exposure (ultrabrief SDI) + DTMS<br>(2) Exposure (ultrabrief SDI) + Sham DTMS	Somatic	4 weeks
Isserles et al. (2013)[22]	PTSD (CAPS)	Distress	18	(1) 49 (12.5)<br>(2) 40.4 (10.5)	16.7	(1) Exposure therapy + DTMS<br>(2) Exposure therapy + Sham DTMS	Somatic	4 weeks
Janse et al. (2020)[75]	Somatoform, mood, and/or anxiety disorder (SCL-90-R, GSI)	-	368	(1) 40.4 (11.9)<br>(2) 42.6 (12.3)	57.9	(1) CBT + High-intensity progress monitoring (feedback)<br>(2) CBT + Low-intensity feedback monitoring	Psychological/cognitive	33 weeks
Kamboj et al. (2012)[76]	Nicotine dependence (VAS)	Substance abuse	32	(1) 30 (9.37)<br>(2) 32.31 (10.64)	31.2	(1) Exposure/response prevention + D-cycloserine<br>(2) Exposure/response prevention + Placebo	Pharmacological	Two sessions
Kozel et al. (2018)[23]	PTSD (CAPS)	Distress	61	(1) 34.06 (7.56)<br>(2) 32.93 (6.04)	Not reported	(1) Cognitive processing therapy + rTMS<br>(2) Cognitive processing therapy + Sham rTMS	Somatic	12 weeks
Krupitsky et al. (2002)[77]	Heroin dependence (VAS, craving)	Substance abuse	70	(1) 23 (4.4)<br>(2) 21.6 (3)	21.4	(1) Psychotherapy + Ketamine (2mg)<br>(2) Psychotherapy + Ketamine (0.2mg)	Pharmacological	Single session
Kushner et al. (2007)[78]	OCD (Y-BOCS)	Fear	25	Not reported	Not reported	(1) Exposure/ritual prevention therapy + D-cycloserine<br>(2) Exposure/ritual prevention therapy + Placebo	Pharmacological	5 weeks
Kwee et al. (2022)[79]	Panic disorder with agoraphobia or social anxiety (Fear Questionnaire)	Fear	80	(1) 34.9 (9.3)<br>(2) 38.3 (11.3)	40	(1) Exposure therapy + Cannabidiol<br>(2) Exposure therapy + Placebo	Pharmacological	8 weeks
Lancaster et al. (2020)[80]	Fear of spiders or snakes (BAT-T, step no.)	Fear	166	(1) 19.51 (2.20)<br>(2) 19.27 (1.46)	80	(1) Exposure therapy + Pre-extinction fear memory reactivation & deepened extinction<br>(2) Exposure therapy	Psychological/cognitive	1 day
Lass-Hennemann & Michael (2014)[81]	Spider phobia (BAT)	Fear	60	(1) 23.33 (1.12)<br>(2) 25.87 (1.06)	100	(1) Exposure therapy + High endogenous cortisol levels (morning)<br>(2) Exposure therapy + Low endogenous cortisol (evening)	Somatic	Single session
Lazarov et al. (2018)[14]	Social anxiety (LSAS)	Fear	50	(1) 33.60 (5.14)<br>(2) 35.54 (10.62)	36	(1) CBGT + Attention bias modification<br>(2) CBGT + Placebo	Psychological/cognitive	18 weeks
Lee et al. (2020)[82]	MDD (Patient recall task)	Distress	42	(1) 42.18 (8.10)<br>(2) 45.25 (12.02)	54.8	(1) Cognitive therapy + Memory support<br>(2) Cognitive therapy	Psychological/cognitive	14 weeks
Lehrner et al. (2021)[83]	PTSD (CAPS)	Distress	60	(1) 36.07 (8.07)<br>(2) 34.63 (7.68)	90	(1) Prolonged exposure + Hydrocortisone<br>(2) Prolonged exposure + Placebo	Pharmacological	12 weeks
Leuchter et al. (2022)[84]	Depression (FSQ)	Distress	17	(1) 21.1 (2.3)<br>(2) 21.1 (3.8)	64.7	(1) Exposure therapy + rTMS (vmPFC)<br>(2) Exposure therapy + vertex rTMS	Somatic	3 sessions over 2 weeks
Litz et al. (2012)[85]	PTSD (CAPS)	Distress	26	(1) 32.77 (9.85)<br>(2) 31.62 (9.10)	0	(1) Exposure therapy + D-cycloserine<br>(2) Exposure therapy + Placebo	Pharmacological	6 weeks
Maples-Keller et al. (2017)[86]	Fear of flying (FFI)	Fear	89	(1) 43.4 (11.32)<br>(2) 40.8 (12)	79.8	(1) VRET + Reactivation cue<br>(2) VRET + Neutral cue	Psychological/cognitive	8 weeks
Marker et al. (2020)[87]	Anxiety, OCD and/or PTSD (ADIS-5, CSR)	Fear	36	(1) 41.56 (12.89)<br>(2) 40.61 (17.61)	55.6	(1) CBT + Motivational interviewing<br>(2) CBT + Psychoeducation	Psychological/cognitive	12 weeks
McEvoy et al. (2020)[9]	Social anxiety (SIAS)	Fear	105	(1) 30.14 (12.79)<br>(2) 27.06 (9.71)	49.5	(1) CBT + Imagery-enhanced CBT<br>(2) CBT + Verbally-based CBT	Psychological/cognitive	12 weeks
Meyer et al. (2010)[88]	OCD (Y-BOCS)	Fear	93	*38.6 (12.5)	75.3	(1) CBGT + Motivational interviewing and thought mapping<br>(2) CBGT	Psychological/cognitive	12 weeks
Meyer et al. (2022)[89]	Depression (HAM-D)	Distress	10	(1) 46 (range 35-59)	90	(1) Online CBT + Moderate-intensity exercise (ActiveCBT)	Somatic	8 weeks

				(2) 44 (range 19-51)		(2) Online CBT + Usual activities before therapy (CalmCBT)		
Meyerbroeker et al. (2012)[90]	Fear of flying (FAM)	Fear	45	Not reported	Not reported	(1) Virtual reality exposure therapy + Yohimbine Hydrochloride<br>(2) Virtual reality exposure therapy + Placebo	Pharmacological	4 weeks
Meyerbröker et al. (2018)[91]	Fear of flying (FAM)	Fear	54	*36.6 (12.1)	55	(1) VRET + Yohimbine Hydrochloride & propranolol<br>(2) VRET + Placebo	Pharmacological	Not reported
Mithoefer et al. (2011)[92]	PTSD (CAPS)	Distress	20	(1) 40.2 (7.6)<br>(2) 40.8 (7)	85	(1) Psychotherapy + MDMA<br>(2) Psychotherapy + Placebo	Pharmacological	Not reported
Mithoefer et al. (2018)[93]	PTSD (CAPS)	Distress	26	(1) 36.43 (10.66)<br>(2) 39.2 (9.7)	27	(1) Psychotherapy + MDMA (75 or 125mg)<br>(2) Psychotherapy + MDMA (30mg)	Pharmacological	Not reported
Morissette et al. (2008)[94]	Social anxiety (SUDS)	Fear	8	*27.5 (range 22-34)	Not reported	(1) Exposure therapy + Alprazolam & propranolol<br>(2) Exposure therapy + Placebo or no pill	Pharmacological	Two sessions
Moshier & Otto (2017)[95]	MDD (BDI-II)	Distress	34	(1) 36.3 (14.4)<br>(2) 34.38 (15.4)	52	(1) BATD + CCT (Cognitive Control Training)<br>(2) BATD + PVT	Psychological/cognitive	4 weeks
Nations et al. (2012)[96]	Panic disorder (PDSS)	Fear	37	(1) 35.09 (9.76)<br>(2) 32.4 (11.2)	67.5	(1) CBT + Org 25935 (Glycine transporter inhibitor; 4mg or 12mg)<br>(2) CBT + Placebo	Pharmacological	6 weeks
Nejati et al. (2017)[20]	Major depression (BDI-II)	Distress	11	*19.40 (1.96)	Not reported	(1) STPP + tDCS<br>(2) STPP	Somatic	5 weeks
Nicholas et al. (2022)[97]	Comorbid PTSD and substance use disorder (AUDIT)	Substance abuse	90	(1) 44.18 (13.10)<br>(2) 38.53 (10.64)	64.6	(1) Trauma-focused therapy + MDMA<br>(2) Trauma-focused therapy + Placebo	Pharmacological	3-4 weeks
Nord et al. (2019)[18]	Major depression (HAM-D)	Distress	39	(1) 35.60 (12.91)<br>(2) 31.05 (8.17)	51.3	(1) CBT + tDCS<br>(2) CBT + Sham tDCS	Somatic	8 weeks
Oehen et al. (2013)[98]	PTSD (CAPS)	Distress	12	(1) 42.1 (12.8)<br>(2) 40 (6.2)	83	(1) Psychotherapy + MDMA (125mg)<br>(2) Psychotherapy + MDMA (25mg)	Pharmacological	Not reported
Ot'alora G et al. (2018)[99]	PTSD (CAPS)	Distress	28	(1) 42.55 (13.36)<br>(2) 40 (11.7)	67.9	(1) Psychotherapy + MDMA (125mg and 100mg)<br>(2) Psychotherapy + MDMA (40mg)	Pharmacological	Not reported
Otto et al. (2010)[100]	Panic disorder (PDSS)	Fear	27	*35 (11)	50	(1) CBT + D-cycloserine<br>(2) CBT + Placebo	Pharmacological	5 weeks
Pace-Schott et al. (2018)[101]	Social anxiety (LSAS)	Fear	31	(1) 27.24 (6.79)<br>(2) 24.60 (5.49)	56.3	(1) Exposure therapy + Nap<br>(2) Exposure therapy + Wake	Somatic	5 weeks
Powers et al. (2009)[102]	Claustrophobia (BAT, peak fear)	Fear	24	*24.45 (8.65)	79	(1) Exposure therapy + Yohimbine hydrochloride<br>(2) Exposure therapy + Placebo	Pharmacological	Not reported
Pyrkosch et al. (2018)[103]	Agoraphobia (PAS)	Fear	69	(1) 34.11 (10.37)<br>(2) 40.86 (12.94)	65.8	(1) CBT + D-cycloserine<br>(2) CBT + Placebo	Pharmacological	12 weeks
Raeder et al. (2019)[104]	Spider phobia (FSQ)	Fear	43	(1) 22.7 (4.21)<br>(2) 22.57 (3.78)	76.7	(1) Exposure therapy + Cortisol<br>(2) Exposure therapy + Placebo	Pharmacological	Single session
Reinecke et al. (2020)[105]	Panic disorder (Faces dot probe task)	Fear	31	(1) 42.1 (16.7)<br>(2) 41.9 (13.7)	75.8	(1) Exposure therapy + D-cycloserine<br>(2) Exposure therapy + Placebo	Pharmacological	Single session
Reitmaier et al. (2022)[106]	Spider phobia (FSQ)	Fear	53	*24.14 (6.00)	9.6	(1) VRET + Rhythmic eye movements<br>(2) VRET	Psychological/cognitive	Single session
Rodebaugh et al. (2013)[107]	Social anxiety (SUDS)	Fear	34	(1) 49<br>(2) 38	67	(1) Exposure therapy + D-cycloserine<br>(2) Exposure therapy + Placebo	Pharmacological	2 weeks
Ross et al. (2016)[108]	Anxiety and depression (HADS)	-	26	(1) 52 (15.03)<br>(2) 60.27 (9.45)	62	(1) Psychotherapy + Psilocybin<br>(2) Psychotherapy + Niacin	Pharmacological	Not reported

Ross et al. (2021)[109]	Anxiety or adjustment disorder due to cancer (Suicidal Ideation [item 9 of the BDI & BSI])	Distress	11	(1) 57.5 (7.5)<br>(2) 63.6 (5.3)	63.6	(1) Preparatory and post-integrative psychotherapy + Psilocybin<br>(2) Preparatory and post-integrative psychotherapy + Niacin	Pharmacological	Single session
Rubin et al. (2022)[110]	Social anxiety disorder (PRPSA)	Fear	21	(1) 25.90 (15.42)<br>(2) 19.20 (1.23)	61.9	(1) VRET + Attention guidance tracking<br>(2) VRET	Psychological/cognitive	Single session
Santa Ana et al. (2015)[111]	Cocaine dependence (Craving rating)	Substance abuse	47	(1) 49.2 (8.8)<br>(2) 44.4 (8.7)	14.9	(1) Cue exposure therapy + D-cycloserine<br>(2) Cue exposure therapy + Placebo	Pharmacological	Not reported
Sherman et al. (2017)[112]	Cannabis dependence (TLFB)	Substance abuse	15	*25.5 (7.6)	37.5	(1) Motivational enhancement therapy + Oxytocin<br>(2) Motivational enhancement therapy + Placebo	Pharmacological	4 weeks
Shiban et al. (2017)[26]	Aviophobia (FFS)	Fear	29	(1) 34.3 (9.81)<br>(2) 43 (9.96)	82.8	(1) VRET + Diaphragmatic breathing<br>(2) VRET	Somatic	2 weeks
Siegmund et al. (2011)[113]	Panic disorder (PAS)	Fear	39	(1) 37.85 (11.3)<br>(2) 37.32 (13)	46.2	(1) CBT + D-cycloserine<br>(2) CBT	Pharmacological	Not reported
Simpson et al. (2010)[114]	OCD (Y-BOCS)	Fear	30	(1) 40.7 (11.1)<br>(2) 39.1 (15.7)	47	(1) Exposure and response prevention + Motivational interviewing<br>(2) Exposure and response prevention	Psychological/cognitive	8 weeks
Sippel et al. (2020)[115]	PTSD (PCL-5)	Distress	17	(1) 41.63 (14.58)<br>(2) 45.78 (15.09)	17.6	(1) Prolonged exposure + Oxytocin<br>(2) Prolonged exposure + Placebo	Pharmacological	10 weeks
Smits et al. (2006)[116]	Social anxiety (LSAS)	Fear	68	(1) 22.99 (7.55)<br>(2) 20.42 (4.43)	57	(1) Exposure therapy + Videotape feedback<br>(2) Exposure therapy + No feedback	Psychological/cognitive	1 week
Smits et al. (2014)[117]	Social anxiety (LSAS)	Fear	40	(1) 35.50 (14.02)<br>(2) 38.70 (12.08)	35	(1) Exposure therapy + Yohimbine hydrochloride<br>(2) Exposure therapy + Placebo	Pharmacological	5 weeks
Smits et al. (2020a)[118]	Social anxiety (LSAS)	Fear	152	(1) 29.39 (9.61)<br>(2) 28.76 (11.81)	55.3	(1) Exposure therapy + D-cycloserine<br>(2) Exposure therapy + Placebo	Pharmacological	5 weeks
Smits et al. (2020b)[119]	Nicotine dependence (ASI-3)	Substance abuse	53	(1) 35.19 (12.37)<br>(2) 36.64 (12.85)	69.1	(1) Exposure therapy + D-cycloserine<br>(2) Exposure therapy + Placebo	Pharmacological	7 weeks
Soravia et al. (2014)[120]	Spider phobia (FSQ)	Fear	22	(1) 33.1 (2.9)<br>(2) 30.5 (3.7)	77.3	(1) Exposure therapy + Cortisol<br>(2) Exposure therapy + Placebo	Pharmacological	2 weeks
Soucy et al. (2021)[121]	Generalised anxiety (GAD-7)	Distress	434	(1) 37.80 (12.98)<br>(2) 37.55 (12.88)	74.9	(1) iCBT + Motivational interviewing<br>(2) iCBT	Psychological/cognitive	25 weeks
Steudte-Schmiedgen et al. (2021)[122]	Arachnophobia (SAS)	Fear	33	(1) 25.12 (7.98)<br>(2) 24.25 (6.13)	78.8	(1) CBT (exposure-based) + Hydrocortisone<br>(2) CBT (exposure-based) + Placebo	Pharmacological	Single session
Storch et al. (2007)[123]	OCD (Y-BOCS)	Fear	24	(1) 32 (9.4)<br>(2) 25.9 (9.7)	50	(1) Exposure and response prevention therapy + D-cycloserine<br>(2) Exposure and response prevention therapy + Placebo	Pharmacological	12 weeks
Tart et al. (2013)[124]	Acrophobia (BAT)	Fear	29	(1) 29.33 (14.67)<br>(2) 37.71 (16.81)	Not reported	(1) VRET + D-cycloserine<br>(2) VRET + Placebo	Pharmacological	2 weeks
Thierrée et al. (2021)[125]	PTSD (CAPS)	Distress	38	(1) 31.3 (10.0)<br>(2) 33.5 (11.1)	65.8	(1) Exposure therapy (trauma script) + rTMS (hi-freq; 110% motor threshold)<br>(2) Exposure therapy (trauma script) + rTMS (low-freq; 70% motor threshold)	Somatic	>2 weeks (8 sessions)
Tuerk et al. (2018)[126]	PTSD (Trauma-cued heart-rate reactivity)	Distress	26	(1) 34.6 (5.5)<br>(2) 29.9 (6.7)	0	(1) Prolonged exposure + Yohimbine hydrochloride<br>(2) Prolonged exposure + Placebo	Pharmacological	15 weeks
Vermes et al. (2020)[127]	PTSD (SCR)	Distress	42	(1) 34.7 (9.81)<br>(2) 37.4 (11)	90.5	(1) Imaginal exposure + Trauma retrieval<br>(2) Imaginal exposure + Neutral retrieval	Psychological/cognitive	1 week
Weingarden et al. (2019)[128]	Body dysmorphic disorder (BDD-YBOCS)	-	26	(1) 23.92 (5.74)<br>(2) 29.07 (11.04)	65.4	(1) CBT + D-cycloserine<br>(2) CBT + Placebo	Pharmacological	10 weeks

Wilhelm et al. (2008)[129]	OCD (Y-BOCS)	Fear	23	(1) 40 (13.4)<br>(2) 38.2 (13)	Not reported	(1) Behaviour therapy + D-cycloserine<br>(2) Behaviour therapy + Placebo	Pharmacological	5 weeks
Witkiewitz et al. (2019)[17]	Alcohol use disorder (TLFB [drinks per drinking day])	Substance abuse	84	(1) 51.43 (14.19)<br>(2) 53.35 (11.41)	40.5	(1) Mindfulness-based relapse prevention + tDCS<br>(2) Mindfulness-based relapse prevention + Sham tDCS	Somatic	8 weeks
Wolfson et al. (2020)[130]	Anxiety (STAI)	Fear	18	(1) 55.5 (7)<br>(2) 53.2 (10.5)	77.8	(1) Psychotherapy + MDMA<br>(2) Psychotherapy + Placebo	Pharmacological	Not reported
Zoellner et al. (2017)[131]	PTSD (PSS-I)	Distress	31	*37.5 (12.4)	71.4	(1) Imaginal exposure + Methylene blue<br>(2) Imaginal exposure + Placebo	Pharmacological	1 week

* indicates the average age between both groups; N: Sample Size; SD: Standard Deviation; PHARM: Pharmacological Augmentations; PSYC: Psychological/Cognitive Augmentations; SOM: Somatic Interventions.

Mental health dimensions and assessment measures: ADIS-5, CSR: Anxiety and Related Disorders Interview Schedule for DSM-5, Clinician Severity Rating; AQ: Acrophobia Questionnaire; ASI-3: Anxiety Sensitivity Index; AUDIT: Alcohol Use Disorders Identification Test; BAT: Behavioural Approach Task; BDD: Body Dysmorphic Disorder; BDD-YBOCS: Body Dysmorphic Disorder Modification Of The Yale-Brown Obsessive-Compulsive Scale ; BDI: Beck Depression Inventory; BSFS: Birchwood Social Functioning Scale; BSI: Brief Symptom Inventory; BSS: Body Satisfaction Scale; CAPS: Clinically Administered Post-Traumatic Stress Disorder Scale; FAM: Flight Anxiety Modality Questionnaire; FCC: Fluid Cognitive Composite; FFI: Fear Of Flying Inventory; FFS: Fear Of Flying Scale; FSQ: Fear Of Spiders Questionnaire; GAD-7: Generalised Anxiety Disorder Assessment; GSI: Global Severity Index; HAM-A: Hamilton Anxiety Rating Scale; HAM-D: Hamilton Depression Rating Scale; IDS-SR: Inventory Of Depressive Symptomatology – Self-Report; IES-R: Impact Of Event Scale – Revised; LSAS: Liebowitz Social Anxiety Scale; MADRS: Montgomery–Åsberg Depression Rating Scale; MDD: Major Depressive Disorder; MI: Mobility Inventory; NFQ: Nightmare Frequency Questionnaire; OCD: Obsessive Compulsive Disorder; OCDUS: Obsessive-Compulsive Drug Use Scale; PAS: Panic And Agoraphobia Scale; PCL-5: Post-Traumatic Stress Disorder Checklist for DSM-5; PCL-M: Post-Traumatic Stress Disorder Checklist – Military; PDSS: Panic Disorder Severity Scale; PRPSA: Personal Report of Public Speaking Apprehension; PSQI: Pittsburgh Sleep Quality Index; PSS-I: Post-Traumatic Symptom Scale – Interview; PSS-SR: Post-Traumatic Stress Disorder Symptom Scale – Self Report Version; PSYRATS-D: Psychotic Symptom Rating Scale (delusion subscale); PTSD: Post-Traumatic Stress Disorder; SAS: Spider Anxiety Screening; SCL-90-R: Symptom Checklist – 90 – Revised; SCR: Skin Conductance Response; SIAS: Social Interaction Anxiety Scale; SPAI: Social Phobia And Anxiety Inventory; SSSPTSD: Severity Of Symptoms Scale For Post-Traumatic Stress Disorder; SUDS: Subjective Units Of Distress Scale; TBI: Target Bias Index; TLFB: Timeline Followback; VAS: Visual Analogue Scale; Y-BOCS: Yale-Brown Obsessive-Compulsive Scale.

Psychological therapies, augmentation and control groups: ABM: Attention Bias Modification; BATD: Behavioural Activation Therapy For Depression; CBGT: Cognitive Behavioural Group Therapy; CBI: Cognitive Behavioural Intervention; CBM-I: Cognitive Bias Modification for Interpretation; CBSST: Cognitive Behavioural Social Skills Training; CBT: Cognitive Behavioural Therapy; CBT-I: Cognitive Behavioural Therapy For Insomnia; CCT: Cognitive Control Training; CPT: Cognitive Processing Therapy CR: Cognitive Restructuring; ERP: Exposure And Response Prevention; DTMS: Deep Transcranial Magnetic Stimulation; ICBT: Internet Cognitive Behavioural Therapy; IE: Imaginal Exposure; IVE: In Vivo Exposure; MET: Motivational Enhancement Therapy; MBRP: Mindfulness Based Relapse Prevention; MDMA: 3,4-Methylenedioxy methamphetamine; PVT: Peripheral Vision Training; rTMS: Repetitive Transcranial Magnetic Stimulation; STPP: Short-Term Psychoanalytic Psychotherapy; tDCS: Transcranial Direct Current Stimulation; TEAS: Transcutaneous Electrical Acupuncture Stimulation; VR: Virtual Reality; VRET: Virtual Reality Exposure Therapy; YOH: Yohimbine Hydrochloride.

2.2. Outlier analyses

Supplemental Table S3. Studies defined as outliers (confidence intervals not overlapping with the confidence intervals of the pooled effect).

Authors (year of publication)	Mental health measure (assessment)	Mean (SD) age (1) Augmentation (2) Control group	Gender (% female)	(1) Augmentation group(s) & (2) Control group	Type of augmentation	Duration
Acheson et al. (2015)[1]	Arachnophobia (FSQ)	(1) 33.3 (4.01)<br>(2) 26.77 (2.67)	82.6	(1) Exposure therapy + Oxytocin<br>(2) Exposure therapy + Placebo	Pharmacological	Single session
Andersson et al. (2015)[2]	OCD (Y-BOCS)	(1) 34.64 (12.42)<br>(2) 34.88 (12.22)	58	(1) ICBT + D-cycloserine<br>(2) ICBT + Placebo	Pharmacological	12 weeks
Davis et al. (2021)[3]	MDD (GRID-HAMD)	(1) 43.6 (13)<br>(2) 35.2 (9.9)	66.7	(1) Psychotherapy + Psilocybin<br>(2) Wait list (no therapy or augmentation)	Pharmacological	8 weeks
Falkenstein et al. (2022)[4]	OCRDs (TBI, interpretation)	(1) 30.36 (8.44)<br>(2) 27.25 (3.77)	50	(1) IRT + CBM-I<br>(2) IRT + Psychoeducation	Psychological/ cognitive	~4 weeks
Fayaz et al. (2022)[5]	Methamphetamine use disorder (OCDUS)	(1) 34 (6.7)<br>(2) 37 (7)	0	(1) Matrix Model psychotherapy + tDCS<br>(2) Matrix Model psychotherapy + Sham tDCS	Somatic	12 weeks
Feng et al. (2019)[6]	PTSD (CAPS)	(1) 40.9 (12.7)<br>(2) 41 (12.8)	69.2	(1) CBT + Transcutaneous electrical acupoint stimulation<br>(2) CBT + Simulated TEAS	Somatic	12 weeks
Harb et al. (2019)[7]	PTSD (NFQ)	(1) 37 (9.9)<br>(2) 37.2 (10.1)	13.9	(1) CBT-I + Imagery rescripting<br>(2) CBT-I	Psychological /cognitive	6 weeks
Isserles et al. (2021)[8]	PTSD (CAPS)	(1) 44.8 (13.19)<br>(2) 43.7 (12.25)	66.4	(1) Exposure (ultrabrief SDI) + DTMS<br>(2) Exposure (ultrabrief SDI) + Sham DTMS	Somatic	4 weeks
Krupitsky et al. (2002)[9]	Heroin dependence (VAS, craving)	(1) 23 (4.4)<br>(2) 21.6 (3)	21.4	(1) Psychotherapy + Ketamine (2mg)<br>(2) Psychotherapy + Ketamine (0.2mg)	Pharmacological	Single session
Lass-Hennemann & Michael (2014)[10]	Spider phobia (BAT)	(1) 23.33 (1.12)<br>(2) 25.87 (1.06)	100	(1) Exposure therapy + High endogenous cortisol levels (morning)<br>(2) Exposure therapy + Low endogenous cortisol (evening)	Somatic	Single session
Litz et al. (2012)[11]	PTSD (CAPS)	(1) 32.77 (9.85)<br>(2) 31.62 (9.10)	0	(1) Exposure therapy + D-cycloserine<br>(2) Exposure therapy + Placebo	Pharmacological	6 weeks
McEvoy et al. (2020)[12]	Social anxiety (SIAS)	(1) 30.14 (12.79)<br>(2) 27.06 (9.71)	49.5	(1) CBT + Imagery-enhanced CBT<br>(2) CBT + Verbally-based CBT	Psychological/ cognitive	12 weeks
Meyerbröker et al. (2018)[13]	Fear of flying (FAM)	*36.6 (12.1)	55	(1) VRET + Yohimbine Hydrochloride & propranolol<br>(2) VRET + Placebo	Pharmacological	Not reported
Mithoefer et al. (2011)[14]	PTSD (CAPS)	(1) 40.2 (7.6)<br>(2) 40.8 (7)	85	(1) Psychotherapy + MDMA<br>(2) Psychotherapy + Placebo	Pharmacological	Not reported
Ross et al. (2016)[15]	Anxiety and depression (HADS)	(1) 52 (15.03)<br>(2) 60.27 (9.45)	62	(1) Psychotherapy + Psilocybin<br>(2) Psychotherapy + Niacin	Pharmacological	Not reported
Siegmund et al. (2011)[16]	Panic disorder (PAS)	(1) 37.85 (11.3)<br>(2) 37.32 (13)	46.2	(1) CBT + D-cycloserine<br>(2) CBT	Pharmacological	Not reported

Supplemental Figure S1. Results from random effect meta-analysis excluding outliers. Main effect (k = 92 studies): Hedges' g = -0.21, 95% CI = (-0.27, -0.14); p < 0.0001.

Study	Augmentation N	Augmentation Mean	Augmentation SD	Control N	Control Mean	Control SD	SMD	95% CI	Weight
Alladin & Alibhai, 2007	42	17.50	8.50	42	21.30	7.20	−0.48	[−0.91; −0.04]	1.8%
Arntz et al., 2007	28	18.52	14.50	39	17.63	13.76	0.06	[−0.42; 0.55]	1.5%
Aust et al., 2022	43	14.40	6.90	42	15.70	7.30	−0.18	[−0.61; 0.24]	1.8%
Baker et al., 2020	11	3.36	3.11	12	6.82	4.09	−0.91	[−1.78; −0.04]	0.5%
Bischoff et al., 2018	39	12.00	7.60	38	13.00	7.50	−0.13	[−0.58; 0.32]	1.7%
Bouso et al., 2008	3	28.30	10.48	2	40.00	1.41	−0.98	[−3.12; 1.15]	0.1%
Brooks et al., 2021	10	−47.70	8.60	13	−49.80	10.70	0.21	[−0.62; 1.03]	0.6%
Bryant et al., 2005	30	11.30	9.98	33	16.58	12.50	−0.46	[−0.96; 0.04]	1.4%
Bryant et al., 2008	59	43.27	35.11	59	55.72	29.54	−0.38	[−0.75; −0.02]	2.4%
Buchanan et al., 2021	22	125.30	20.30	20	126.30	17.40	−0.05	[−0.66; 0.55]	1.0%
Burton et al., 2019	10	48.70	12.70	10	53.60	15.40	−0.33	[−1.22; 0.55]	0.5%
Cesa et al., 2013	27	45.00	13.90	39	49.97	14.30	−0.35	[−0.84; 0.15]	1.4%
Chien et al., 2015	43	11.58	4.51	46	12.74	4.19	−0.26	[−0.68; 0.15]	1.9%
Cobb et al., 2021	23	7.83	11.52	19	12.74	17.75	−0.33	[−0.94; 0.28]	1.0%
Coffey et al., 2016	40	20.49	17.87	86	21.54	19.57	−0.05	[−0.43; 0.32]	2.3%
Contalbrigo et al., 2017	12	0.35	0.19	10	0.67	0.43	−0.96	[−1.85; −0.06]	0.5%
Craske et al., 2019	39	0.20	0.56	21	0.58	0.50	−0.69	[−1.24; −0.15]	1.2%
Danforth et al., 2018	8	46.40	15.20	4	64.00	13.30	−1.11	[−2.42; 0.21]	0.2%
Davis et al., 2014	11	0.11	0.70	11	0.35	0.80	−0.31	[−1.15; 0.53]	0.5%
Davis et al., 2017	26	33.54	8.41	47	32.59	12.61	0.08	[−0.40; 0.56]	1.5%
De Kleine et al., 2012	33	34.33	37.11	34	53.65	38.19	−0.51	[−0.99; −0.02]	1.5%
de Leeuw et al., 2017	19	16.60	4.20	20	18.00	5.20	−0.29	[−0.92; 0.34]	0.9%
De Quervain et al., 2011	20	30.40	13.86	20	40.20	13.86	−0.69	[−1.33; −0.05]	0.9%
Difede et al., 2014	13	32.38	28.55	12	42.17	20.75	−0.38	[−1.17; 0.42]	0.6%
Diminich et al., 2020	22	11.91	5.26	22	12.18	3.79	−0.06	[−0.65; 0.53]	1.0%
Flanagan et al., 2018	8	13.00	12.74	9	17.71	9.62	−0.40	[−1.36; 0.56]	0.4%
Foa et al., 2005	74	16.80	13.20	79	17.90	14.50	−0.08	[−0.40; 0.24]	3.0%
Fryml et al., 2019	5	36.00	36.71	3	43.30	35.23	−0.18	[−1.61; 1.26]	0.2%
Guastella et al., 2008	28	89.52	22.63	28	99.30	27.26	−0.38	[−0.91; 0.14]	1.3%
Guastella et al., 2009	12	96.57	20.19	13	91.41	30.59	0.19	[−0.60; 0.98]	0.6%
Harvey et al., 2016	22	19.41	11.69	20	25.45	10.83	−0.52	[−1.14; 0.09]	1.0%
Hofmann et al., 2006	10	81.74	27.01	13	96.21	37.74	−0.42	[−1.25; 0.42]	0.5%
Hofmann et al., 2013	87	2.68	1.41	82	2.95	1.43	−0.19	[−0.49; 0.11]	3.2%
Hofmeijer−Sevink et al., 2017	38	2.15	2.32	19	1.88	2.48	0.11	[−0.44; 0.66]	1.2%
Hutschemaekers et al., 2021	27	38.22	21.22	27	36.88	20.84	0.06	[−0.47; 0.60]	1.2%
Isserles et al., 2013	9	61.00	26.40	9	76.00	32.10	−0.49	[−1.43; 0.45]	0.4%
Janse et al., 2020	190	0.41	0.40	178	0.41	0.39	0.01	[−0.19; 0.22]	5.3%
Kamboj et al., 2012	16	63.11	25.83	16	56.38	28.29	0.24	[−0.45; 0.94]	0.8%
Kozel et al., 2018	31	31.11	22.49	30	38.29	24.43	−0.30	[−0.81; 0.20]	1.4%
Kushner et al., 2007	14	10.90	4.70	11	11.20	6.80	−0.05	[−0.84; 0.74]	0.6%
Kwee et al., 2022	39	35.38	18.24	39	37.13	17.26	−0.10	[−0.54; 0.35]	1.7%
Lancaster et al., 2020	97	6.95	1.71	33	6.85	1.84	0.06	[−0.34; 0.45]	2.1%
Lazarov et al., 2018	25	48.37	24.25	25	60.05	20.70	−0.51	[−1.07; 0.05]	1.1%
Lee et al., 2020	22	−9.86	6.13	20	−7.94	3.37	−0.38	[−0.99; 0.24]	1.0%
Lehrner et al., 2021	24	52.89	16.31	26	48.94	17.49	0.23	[−0.33; 0.79]	1.1%
Leuchter et al., 2022	8	71.13	21.85	9	80.22	21.31	−0.40	[−1.36; 0.56]	0.4%
Maples−Keller et al., 2017	45	65.55	49.88	44	76.57	47.46	−0.22	[−0.64; 0.19]	1.9%
Marker et al., 2020	18	2.43	1.28	18	2.85	1.28	−0.32	[−0.98; 0.34]	0.8%
Meyer et al., 2010	48	8.15	5.43	45	13.90	9.39	−0.75	[−1.17; −0.33]	1.9%
Meyer et al., 2022	5	2.21	3.35	5	5.62	3.47	−0.90	[−2.24; 0.43]	0.2%
Meyerbroeker et al., 2012	20	26.87	19.64	25	23.96	17.10	0.16	[−0.43; 0.75]	1.0%
Mithoefer et al., 2018	19	37.49	30.12	7	76.00	23.40	−1.30	[−2.25; −0.36]	0.4%
Morissette et al., 2008	4	5.15	1.05	4	4.40	1.33	0.54	[−0.89; 1.98]	0.2%
Moshier & Otto, 2017	21	23.10	10.80	13	18.60	13.30	0.37	[−0.33; 1.07]	0.8%
Nations et al., 2012	24	6.64	3.78	13	6.60	4.29	0.01	[−0.67; 0.68]	0.8%
Nejati et al., 2017	4	12.25	5.62	7	22.99	7.66	−1.39	[−2.81; 0.03]	0.2%
Nicholas et al., 2022	42	3.24	3.36	40	3.23	3.65	0.00	[−0.43; 0.44]	1.8%
Nord et al., 2019	20	12.65	6.91	19	14.37	5.81	−0.26	[−0.89; 0.37]	0.9%
Oehen et al., 2013	8	50.80	19.70	4	66.50	7.60	−0.85	[−2.12; 0.42]	0.2%
Ot'alora G et al., 2018	22	66.74	30.78	6	73.30	24.50	−0.21	[−1.12; 0.69]	0.5%
Otto et al., 2010	13	3.58	1.96	14	6.77	3.30	−1.13	[−1.95; −0.31]	0.6%
Pace−Schott et al., 2018	17	62.82	23.68	14	55.29	19.27	0.34	[−0.38; 1.05]	0.7%
Powers et al., 2009	12	15.42	13.39	12	27.08	21.37	−0.63	[−1.45; 0.19]	0.6%
Pyrkosch et al., 2018	36	21.16	8.81	33	19.79	9.89	0.15	[−0.33; 0.62]	1.5%
Raeder et al., 2019	20	26.05	14.35	23	32.17	19.72	−0.34	[−0.95; 0.26]	1.0%
Reinecke et al., 2020	17	2.10	17.20	14	21.30	30.80	−0.77	[−1.51; −0.03]	0.7%
Reitmaier et al., 2022	27	64.45	19.13	26	56.46	24.76	0.36	[−0.19; 0.90]	1.2%
Rodebaugh et al., 2013	18	55.89	20.46	16	65.85	20.19	−0.48	[−1.16; 0.21]	0.8%
Ross et al., 2021	6	40.33	10.97	5	49.01	17.87	−0.55	[−1.77; 0.67]	0.3%
Rubin et al., 2022	10	123.50	21.21	8	119.63	19.54	0.18	[−0.75; 1.11]	0.4%
Santa Ana et al., 2015	23	0.76	1.37	24	1.42	1.89	−0.39	[−0.97; 0.19]	1.1%
Sherman et al., 2017	8	1.05	1.65	7	0.79	1.39	0.16	[−0.86; 1.18]	0.4%
Shiban et al., 2017	15	1.06	0.63	14	1.29	0.84	−0.30	[−1.04; 0.43]	0.7%
Simpson et al., 2010	15	11.90	2.35	15	13.75	3.60	−0.59	[−1.33; 0.14]	0.7%
Sippel et al., 2020	6	20.50	19.53	7	29.71	26.39	−0.36	[−1.47; 0.74]	0.3%
Smits et al., 2006	35	58.48	24.33	33	58.97	23.01	−0.02	[−0.50; 0.46]	1.5%
Smits et al., 2014	20	38.78	10.24	20	47.19	10.49	−0.80	[−1.44; −0.15]	0.9%
Smits et al., 2020a	114	60.56	23.34	38	65.88	25.16	−0.22	[−0.59; 0.15]	2.3%
Smits et al., 2020b	27	15.37	10.38	26	17.80	13.77	−0.20	[−0.74; 0.34]	1.2%
Soravia et al., 2014	11	43.30	11.28	11	53.50	17.58	−0.66	[−1.53; 0.20]	0.5%
Soucy et al., 2021	203	6.26	4.78	231	6.22	5.05	0.01	[−0.18; 0.20]	5.8%
Steudte−Schmiedgen et al., 2021	17	14.17	2.39	16	15.95	3.92	−0.54	[−1.24; 0.16]	0.8%
Storch et al., 2007	12	10.10	6.80	12	8.60	8.80	0.18	[−0.62; 0.99]	0.6%
Tart et al., 2013	15	29.73	25.67	14	35.55	25.18	−0.22	[−0.95; 0.51]	0.7%
Thierree et al., 2021	13	41.80	31.90	16	51.60	23.70	−0.34	[−1.08; 0.39]	0.7%
Tuerk et al., 2018	14	71.01	14.37	12	75.08	10.77	−0.31	[−1.08; 0.47]	0.6%
Vermes et al., 2020	21	0.36	0.60	21	0.51	0.44	−0.28	[−0.89; 0.33]	1.0%
Weingarden et al., 2019	12	19.64	6.80	14	18.77	10.03	0.10	[−0.67; 0.87]	0.6%
Wilhelm et al., 2008	10	10.20	7.20	13	14.50	6.40	−0.61	[−1.46; 0.23]	0.5%
Witkiewitz et al., 2019	47	5.12	3.14	37	5.64	4.06	−0.14	[−0.58; 0.29]	1.8%
Wolfson et al., 2020	13	38.90	10.60	5	48.60	12.60	−0.83	[−1.91; 0.25]	0.3%
Zoellner et al., 2017	15	17.10	10.57	16	14.67	9.27	0.24	[−0.47; 0.95]	0.7%
**Random effects model**	**2561**			**2407**			**0.21**	**[0.27; 0.14]**	**100.0%**
**Prediction interval**								**[0.40; 0.01]**	

Heterogeneity: I^2 = 10%, □^{2} = 0.0086, p = 0.22

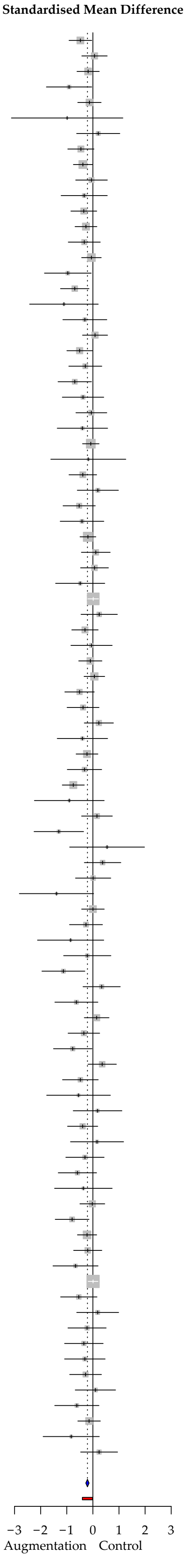

Supplemental Figure S2. Random effects meta-analysis results including only outliers. Main effect (k = 16 studies):
Hedges' g = -0.56, 95% CI = (-1.11, -0.00); p = 0.0489.

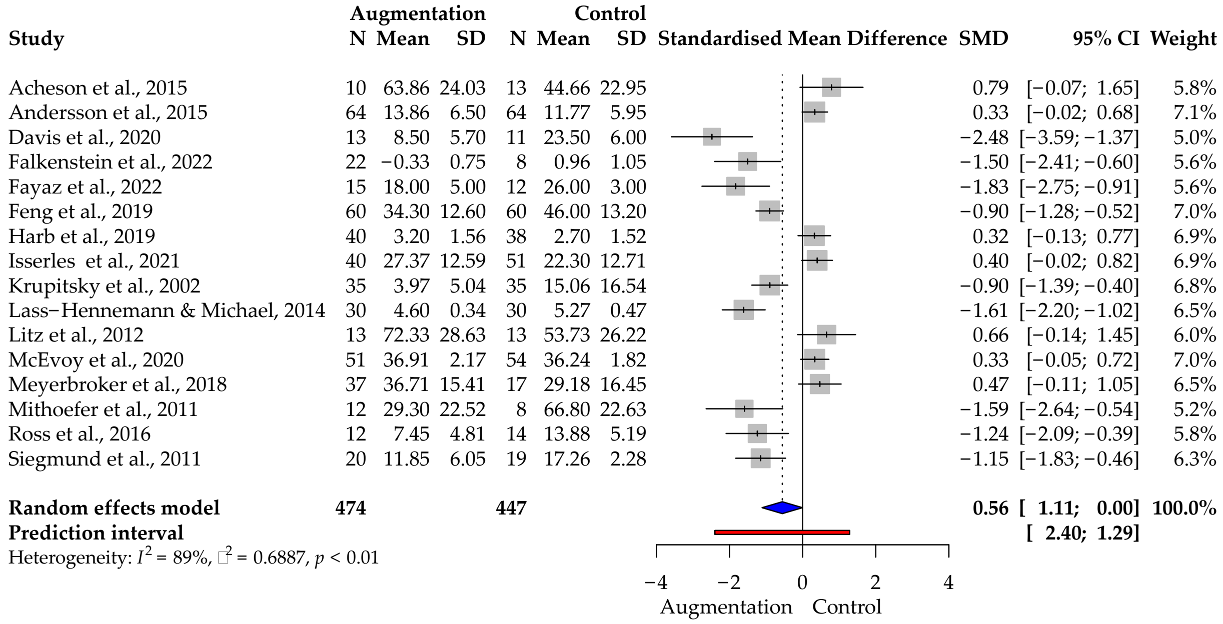

2.3. Risk of bias assessment

Supplemental Table S4. Risk of bias assessment. Studies marked according to the Cochrane Risk of Bias Tool. Green indicates low risk of bias on the specified index, yellow indicates unclear risk of bias on the specified index, and red indicates high risk of bias on the specified index. Selective reporting was not assessed due to our meta-analysis focus on specified (mental health) outcome criteria.

**Authors (year of publication)**	**Mental health measure (assessment)**	**Random sequence generation**	**Allocation concealment**	**Blinding (participants/ personnel)**	**Blinding outcome assessment***	**Incomplete outcome data (attrition)**
Acheson et al. (2015)[1]	Arachnophobia (FSQ)	Green	Yellow	Green	Green	Green
Alladin & Alibhai (2007)[17]	Depression (BDI-II)	Yellow	Yellow	Red	Red	Red
Andersson et al. (2015)[2]	OCD (Y-BOCS)	Green	Green	Green	Green	Green
Arntz et al. (2007)[18]	PTSD (PSS-SR)	Yellow	Red	Red	Red	Red
Aust et al. (2022)[19]	Depression (MADRS)	Green	Green	Green	Green	Red
Baker et al. (2020)[20]	Panic Disorder (PDSS)	Yellow	Yellow	Green	Green	Green
Bischoff et al. (2018)[21]	Panic Disorder with agoraphobia (HAM-A)	Green	Yellow	Red	Green	Green
Bouso et al. (2008)[22]	PTSD (SSSPTSD)	Yellow	Yellow	Green	Green	Green
Brooks et al. (2021)[23]	Depression or anxiety + cognitive decline (FCC)	Yellow	Yellow	Green	Green	Red
Bryant et al. (2005)[24]	Acute stress disorder (IES)	Green	Red	Red	Green	Red
Bryant et al. (2008)[25]	PTSD (CAPS)	Green	Green	Green	Green	Red

Buchanan et al. (2021)[26]	Social functioning in schizophrenia (BSFS)
Burton et al. (2019)[27]	PTSD (PCL-M)
Cesa et al. (2013)[28]	Binge eating disorder (BSS)
Chien et al. (2015)[29]	Depression (PSQI)
Cobb et al. (2021)[30]	Phobia (BAT)
Coffey et al. (2016)[31]	PTSD (IES-R)
Contalbrigo et al. (2017)[32]	Substance use disorder (SCL-90-R)
Craske et al. (2019)[33]	Social anxiety (SCR to public speaking onset)
Danforth et al. (2018)[34]	Social anxiety (LSAS)
Davis et al. (2014)[35]	Schizophrenia (Social-cognition composite score)
Davis et al. (2017)[36]	Social anxiety (LSAS)
Davis et al. (2021)[3]	MDD (GRID-HAMD)
De Kleine et al. (2012)[37]	PTSD (CAPS)
de Leeuw et al. (2017)[38]	OCD (Y-BOCS)
De Quervain et al. (2011)[39]	Acrophobia (AQ)
Difede et al. (2014)[40]	PTSD (CAPS)
Diminich et al. (2020)[41]	Delusions (PSYRATS-D)
Falkenstein et al. (2022)[4]	OCRDs (TBI, interpretation)
Fayaz Feyzi et al. (2022)[5]	Methamphetamine use disorder (OCDUS)
Feng et al. (2019)[6]	PTSD (CAPS)
Flanagan et al. (2018)[42]	PTSD (CAPS)
Foa et al. (2005)[43]	PTSD (PSS-I)
Fryml et al. (2019)[44]	PTSD (CAPS)
Guastella et al. (2008)[45]	Social anxiety (SPAI)
Guastella et al. (2009)[46]	Social anxiety (SPAI)
Harb et al. (2019)[7]	PTSD (NFQ)
Harvey et al. (2016)[47]	MDD (IDS-SR)
Hofmann et al. (2006)[48]	Social anxiety (SPAI)
Hofmann et al. (2013)[49]	Social anxiety (SPD-S)
Hofmeijer-Sevink et al. (2017)[50]	Panic disorder with agoraphobia (Mobility Inventory, alone subscale)
Hutschemaekers et al. (2021)[51]	Social anxiety disorder (SUDS)
Isserles et al. (2021)[8]	PTSD (CAPS)
Isserles et al. (2013)[52]	PTSD (CAPS)

Janse et al. (2020)[53]	Somatoform, mood, and/or anxiety disorder (SCL-90-R, GSI)					
Kamboj et al. (2012)[54]	Nicotine dependence (VAS)					
Kozel et al. (2018)[55]	PTSD (CAPS)					
Krupitsky et al. (2002)[9]	Heroin dependence (VAS, craving)					
Kushner et al. (2007)[56]	OCD (Y-BOCS)					
Kwee et al. (2022)[57]	Panic disorder with agoraphobia or social anxiety (Fear Questionnaire)					
Lancaster et al. (2020)[58]	Fear of spiders or snakes (BAT-T, step no.)					
Lass-Hennemann & Michael (2014)[10]	Spider phobia (BAT)					
Lazarov et al. (2018)[59]	Social anxiety (LSAS)					
Lee et al. (2020)[60]	MDD (Patient recall task)					
Lehrner et al. (2021)[61]	PTSD (CAPS)					
Leuchter et al. (2022)[62]	Depression (FSQ)					
Litz et al. (2012)[11]	PTSD (CAPS)					
Maples-Keller et al. (2017)[63]	Fear of flying (FFI)					
Marker et al. (2020)[64]	Anxiety, OCD and/or PTSD (ADIS-5, CSR)					
McEvoy et al. (2020)[12]	Social anxiety (SIAS)					
Meyer et al. (2010)[65]	OCD (Y-BOCS)					
Meyer et al. (2022)[66]	Depression (HAM-D)					
Meyerbroeker et al. (2012)[67]	Fear of flying (FAM)					
Meyerbröker et al. (2018)[13]	Fear of flying (FAM)					
Mithoefer et al. (2011)[14]	PTSD (CAPS)					
Mithoefer et al. (2018)[68]	PTSD (CAPS)					
Morissette et al. (2008)[69]	Social anxiety (SUDS)					
Moshier & Otto (2017)[70]	MDD (BDI-II)					
Nations et al. (2012)[71]	Panic disorder (PDSS)					
Nejati et al. (2017)[72]	Major depression (BDI-II)					
Nicholas et al. (2022)[73]	Comorbid PTSD and substance use disorder (AUDIT)					
Nord et al. (2019)[74]	Major depression (HAM-D)					
Oehen et al. (2013)[75]	PTSD (CAPS)					
Ot'alora G et al. (2018)[76]	PTSD (CAPS)					

Otto et al. (2010)[77]	Panic disorder (PDSS)
Pace-Schott et al. (2018)[78]	Social anxiety (LSAS)
Powers et al. (2009)[79]	Claustrophobia (BAT, peak fear)
Pyrkosch et al. (2018)[80]	Agoraphobia (PAS)
Raeder et al. (2019)[81]	Spider phobia (FSQ)
Reinecke et al. (2020)[82]	Panic disorder (Faces dot probe task)
Reitmaier et al. (2022)[83]	Spider phobia (FSQ)
Rodebaugh et al. (2013)[84]	Social anxiety (SUDS)
Ross et al. (2016)[15]	Anxiety and depression (HADS)
Ross et al. (2021)[85]	Anxiety or adjustment disorder due to cancer (Suicidal Ideation [item 9 of the BDI & BSI])
Rubin et al. (2022)[86]	Social anxiety disorder (PRPSA)
Santa Ana et al. (2015)[87]	Cocaine dependence (Craving rating)
Sherman et al. (2017)[88]	Cannabis dependence (TLFB)
Shiban et al. (2017)[89]	Aviophobia (FFS)
Siegmund et al. (2011)[16]	Panic disorder (PAS)
Simpson et al. (2010)[90]	OCD (Y-BOCS)
Sippel et al. (2020)[91]	PTSD (PCL-5)
Smits et al. (2006)[92]	Social anxiety (LSAS)
Smits et al. (2014)[93]	Social anxiety (LSAS)
Smits et al. (2020a)[94]	Social anxiety (LSAS)
Smits et al. (2020b)[95]	Nicotine dependence (ASI-3)
Soravia et al. (2014)[96]	Spider phobia (FSQ)
Soucy et al. (2021)[97]	Generalised anxiety (GAD-7)
Steudte-Schmiedgen et al. (2021)[98]	Arachnophobia (SAS)
Storch et al. (2007)[99]	OCD (Y-BOCS)
Tart et al. (2013)[100]	Acrophobia (BAT)
Thierrée et al. (2021)[101]	PTSD (CAPS)
Tuerk et al. (2018)[102]	PTSD (Trauma-cued heart-rate reactivity)
Vermes et al. (2020)[103]	PTSD (SCR)
Weingarden et al. (2019)[104]	Body dysmorphic disorder (BDD-YBOCS)
Wilhelm et al. (2008)[105]	OCD (Y-BOCS)

Witkiewitz et al. (2019)[106]	Alcohol use disorder (TLFB [drinks per drinking day])					
Wolfson et al. (2020)[107]	Anxiety (STAI)					
Zoellner et al. (2017)[108]	PTSD (PSS-I)					

*The fifteen studies coloured red or yellow in this category were labelled 'high risk' due to the importance of blinding of outcome assessment for our meta-analysis; these were excluded in our follow-up analysis in the main text (see 'Risk of bias analysis').

<u>Mental health dimensions and assessment measures</u>: ADIS-5, CSR: Anxiety and Related Disorders Interview Schedule for DSM-5, Clinician Severity Rating; AQ: Acrophobia Questionnaire; ASI-3: Anxiety Sensitivity Index; AUDIT: Alcohol Use Disorders Identification Test; BAT: Behavioural Approach Task; BDD: Body Dysmorphic Disorder; BDD-YBOCS: Body Dysmorphic Disorder Modification Of The Yale-Brown Obsessive-Compulsive Scale ; BDI: Beck Depression Inventory; BSFS: Birchwood Social Functioning Scale; BSI: Brief Symptom Inventory; BSS: Body Satisfaction Scale; CAPS: Clinically Administered Post-Traumatic Stress Disorder Scale; FAM: Flight Anxiety Modality Questionnaire; FCC: Fluid Cognitive Composite; FFI: Fear Of Flying Inventory; FFS: Fear Of Flying Scale; FSQ: Fear Of Spiders Questionnaire; GAD-7: Generalised Anxiety Disorder Assessment; GSI: Global Severity Index; HAM-A: Hamilton Anxiety Rating Scale; HAM-D: Hamilton Depression Rating Scale; IDS-SR: Inventory Of Depressive Symptomatology – Self-Report; IES-R: Impact Of Event Scale – Revised; LSAS: Liebowitz Social Anxiety Scale; MADRS: Montgomery–Åsberg Depression Rating Scale; MDD: Major Depressive Disorder; MI: Mobility Inventory; NFQ: Nightmare Frequency Questionnaire; OCD: Obsessive Compulsive Disorder; OCDUS: Obsessive-Compulsive Drug Use Scale; PAS: Panic And Agoraphobia Scale; PCL-5: Post-Traumatic Stress Disorder Checklist for DSM-5; PCL-M: Post-Traumatic Stress Disorder Checklist – Military; PDSS: Panic Disorder Severity Scale; PRPSA: Personal Report of Public Speaking Apprehension; PSQI: Pittsburgh Sleep Quality Index; PSS-I: Post-Traumatic Symptom Scale – Interview; PSS-SR: Post-Traumatic Stress Disorder Symptom Scale – Self Report Version; PSYRATS-D: Psychotic Symptom Rating Scale (delusion subscale); PTSD: Post-Traumatic Stress Disorder; SAS: Spider Anxiety Screening; SCL-90-R: Symptom Checklist – 90 – Revised; SCR: Skin Conductance Response; SIAS: Social Interaction Anxiety Scale; SPAI: Social Phobia And Anxiety Inventory; SSSPTSD: Severity Of Symptoms Scale For Post-Traumatic Stress Disorder; SUDS: Subjective Units Of Distress Scale; TBI: Target Bias Index; TLFB: Timeline Followback; VAS: Visual Analogue Scale; Y-BOCS: Yale-Brown Obsessive-Compulsive Scale.

<u>Psychological therapies, augmentation and control groups:</u> ABM: Attention Bias Modification; BATD: Behavioural Activation Therapy For Depression; CBGT: Cognitive Behavioural Group Therapy; CBI: Cognitive Behavioural Intervention; CBM-I: Cognitive Bias Modification for Interpretation; CBSST: Cognitive Behavioural Social Skills Training; CBT: Cognitive Behavioural Therapy; CBT-I: Cognitive Behavioural Therapy For Insomnia; CCT: Cognitive Control Training; CPT: Cognitive Processing Therapy CR: Cognitive Restructuring; ERP: Exposure And Response Prevention; DTMS: Deep Transcranial Magnetic Stimulation; ICBT: Internet Cognitive Behavioural Therapy; IE: Imaginal Exposure; IVE: In Vivo Exposure; MET: Motivational Enhancement Therapy; MBRP: Mindfulness Based Relapse Prevention; MDMA: 3,4-Methylenedioxymethamphetamine; PVT: Peripheral Vision Training; rTMS: Repetitive Transcranial Magnetic Stimulation; STPP: Short-Term Psychoanalytic Psychotherapy; tDCS: Transcranial Direct Current Stimulation; TEAS: Transcutaneous Electrical Acupuncture Stimulation; VR: Virtual Reality; VRET: Virtual Reality Exposure Therapy; YOH: Yohimbine Hydrochloride.

Supplementary Figure S3. Random effects meta-analysis results excluding studies with high risk of bias. Main effect (k = 93 studies): Hedges' g = -0.29, 95% CI = (-0.39, -0.19); p < 0.0001.

Study	Augmentation N	Augmentation Mean	Augmentation SD	Control N	Control Mean	Control SD	SMD	95% CI	Weight
Acheson et al., 2015	10	63.86	24.03	13	44.66	22.95	0.79	[−0.07; 1.65]	0.8%
Andersson et al., 2015	64	13.86	6.50	64	11.77	5.95	0.33	[−0.02; 0.68]	1.7%
Aust et al., 2022	43	14.40	6.90	42	15.70	7.30	−0.18	[−0.61; 0.24]	1.6%
Baker et al., 2020	11	3.36	3.11	12	6.82	4.09	−0.91	[−1.78; −0.04]	0.8%
Bischoff et al., 2018	39	12.00	7.60	38	13.00	7.50	−0.13	[−0.58; 0.32]	1.5%
Bouso et al., 2008	3	28.30	10.48	2	40.00	1.41	−0.98	[−3.12; 1.15]	0.2%
Brooks et al., 2021	10	−47.70	8.60	13	−49.80	10.70	0.21	[−0.62; 1.03]	0.8%
Bryant et al., 2005	30	11.30	9.98	33	16.58	12.50	−0.46	[−0.96; 0.04]	1.4%
Bryant et al., 2008	59	43.27	35.11	59	55.72	29.54	−0.38	[−0.75; −0.02]	1.7%
Buchanan et al., 2021	22	125.30	20.30	20	126.30	17.40	−0.05	[−0.66; 0.55]	1.2%
Cesa et al., 2013	27	45.00	13.90	39	49.97	14.30	−0.35	[−0.84; 0.15]	1.4%
Cobb et al., 2021	23	7.83	11.52	19	12.74	17.75	−0.33	[−0.94; 0.28]	1.2%
Coffey et al., 2016	40	20.49	17.87	86	21.54	19.57	−0.05	[−0.43; 0.32]	1.7%
Craske et al., 2019	39	0.20	0.56	21	0.58	0.50	−0.69	[−1.24; −0.15]	1.3%
Danforth et al., 2018	8	46.40	15.20	4	64.00	13.30	−1.11	[−2.42; 0.21]	0.4%
Davis et al., 2014	11	0.11	0.70	11	0.35	0.80	−0.31	[−1.15; 0.53]	0.8%
Davis et al., 2017	26	33.54	8.41	47	32.59	12.61	0.08	[−0.40; 0.56]	1.4%
Davis et al., 2020	13	8.50	5.70	11	23.50	6.00	−2.48	[−3.59; −1.37]	0.6%
De Kleine et al., 2012	33	34.33	37.11	34	53.65	38.19	−0.51	[−0.99; −0.02]	1.4%
de Leeuw et al., 2017	19	16.60	4.20	20	18.00	5.20	−0.29	[−0.92; 0.34]	1.1%
De Quervain et al., 2011	20	30.40	13.86	20	40.20	13.86	−0.69	[−1.33; −0.05]	1.1%
Difede et al., 2014	13	32.38	28.55	12	42.17	20.75	−0.38	[−1.17; 0.42]	0.9%
Diminich et al., 2020	22	11.91	5.26	22	12.18	3.79	−0.06	[−0.65; 0.53]	1.2%
Falkenstein et al., 2022	22	−0.33	0.75	8	0.96	1.05	−1.50	[−2.41; −0.60]	0.7%
Fayaz et al., 2022	15	18.00	5.00	12	26.00	3.00	−1.83	[−2.75; −0.91]	0.7%
Feng et al., 2019	60	34.30	12.60	60	46.00	13.20	−0.90	[−1.28; −0.52]	1.7%
Flanagan et al., 2018	8	13.00	12.74	9	17.71	9.62	−0.40	[−1.36; 0.56]	0.7%
Foa et al., 2005	74	16.80	13.20	79	17.90	14.50	−0.08	[−0.40; 0.24]	1.8%
Fryml et al., 2019	5	36.00	36.71	3	43.30	35.23	−0.18	[−1.61; 1.26]	0.4%
Guastella et al., 2008	28	89.52	22.63	28	99.30	27.26	−0.38	[−0.91; 0.14]	1.3%
Guastella et al., 2009	12	96.57	20.19	13	91.41	30.59	0.19	[−0.60; 0.98]	0.9%
Harb et al., 2019	40	3.20	1.56	38	2.70	1.52	0.32	[−0.13; 0.77]	1.5%
Harvey et al., 2016	22	19.41	11.69	20	25.45	10.83	−0.52	[−1.14; 0.09]	1.2%
Hofmann et al., 2006	10	81.74	27.01	13	96.21	37.74	−0.42	[−1.25; 0.42]	0.8%
Hofmann et al., 2013	87	2.68	1.41	82	2.95	1.43	−0.19	[−0.49; 0.11]	1.8%
Hofmeijer−Sevink et al., 2017	38	2.15	2.32	19	1.88	2.48	0.11	[−0.44; 0.66]	1.3%
Hutschemaekers et al., 2021	27	38.22	21.22	27	36.88	20.84	0.06	[−0.47; 0.60]	1.3%
Isserles et al., 2021	40	27.37	12.59	51	22.30	12.71	0.40	[−0.02; 0.82]	1.6%
Isserles et al., 2013	9	61.00	26.40	9	76.00	32.10	−0.49	[−1.43; 0.45]	0.7%
Kamboj et al., 2012	16	63.11	25.83	16	56.38	28.29	0.24	[−0.45; 0.94]	1.0%
Kozel et al., 2018	31	31.11	22.49	30	38.29	24.43	−0.30	[−0.81; 0.20]	1.4%
Krupitsky et al., 2002	35	3.97	5.04	35	15.06	16.54	−0.90	[−1.39; −0.40]	1.4%
Kushner et al., 2007	14	10.90	4.70	11	11.20	6.80	−0.05	[−0.84; 0.74]	0.9%
Kwee et al., 2022	39	35.38	18.24	39	37.13	17.26	−0.10	[−0.54; 0.35]	1.5%
Lazarov et al., 2018	25	48.37	24.25	25	60.05	20.70	−0.51	[−1.07; 0.05]	1.3%
Lee et al., 2020	22	−9.86	6.13	20	−7.94	3.37	−0.38	[−0.99; 0.24]	1.2%
Lehrner et al., 2021	24	52.89	16.31	26	48.94	17.49	0.23	[−0.33; 0.79]	1.3%
Leuchter et al., 2022	8	71.13	21.85	9	80.22	21.31	−0.40	[−1.36; 0.56]	0.7%
Litz et al., 2012	13	72.33	28.63	13	53.73	26.22	0.66	[−0.14; 1.45]	0.9%
Maples−Keller et al., 2017	45	65.55	49.88	44	76.57	47.46	−0.22	[−0.64; 0.19]	1.6%
Marker et al., 2020	18	2.43	1.28	18	2.85	1.28	−0.32	[−0.98; 0.34]	1.1%
McEvoy et al., 2020	51	36.91	2.17	54	36.24	1.82	0.33	[−0.05; 0.72]	1.7%
Meyer et al., 2010	48	8.15	5.43	45	13.90	9.39	−0.75	[−1.17; −0.33]	1.6%
Meyer et al., 2022	5	2.21	3.35	5	5.62	3.47	−0.90	[−2.24; 0.43]	0.4%
Meyerbroeker et al., 2012	20	26.87	19.64	25	23.96	17.10	0.16	[−0.43; 0.75]	1.2%
Meyerbroker et al., 2018	37	36.71	15.41	17	29.18	16.45	0.47	[−0.11; 1.05]	1.2%
Mithoefer et al., 2011	12	29.30	22.52	8	66.80	22.63	−1.59	[−2.64; −0.54]	0.6%
Mithoefer et al., 2018	19	37.49	30.12	7	76.00	23.40	−1.30	[−2.25; −0.36]	0.7%
Morissette et al., 2008	4	5.15	1.05	4	4.40	1.33	0.54	[−0.89; 1.98]	0.4%
Moshier & Otto, 2017	21	23.10	10.80	13	18.60	13.30	0.37	[−0.33; 1.07]	1.0%
Nations et al., 2012	24	6.64	3.78	13	6.60	4.29	0.01	[−0.67; 0.68]	1.1%
Nejati et al., 2017	4	12.25	5.62	7	22.99	7.66	−1.39	[−2.81; 0.03]	0.4%
Nicholas et al., 2022	42	3.24	3.36	40	3.23	3.65	0.00	[−0.43; 0.44]	1.5%
Nord et al., 2019	20	12.65	6.91	19	14.37	5.81	−0.26	[−0.89; 0.37]	1.1%
Oehen et al., 2013	8	50.80	19.70	4	66.50	7.60	−0.85	[−2.12; 0.42]	0.5%
Ot'alora G et al., 2018	22	66.74	30.78	6	73.30	24.50	−0.21	[−1.12; 0.69]	0.7%
Otto et al., 2010	13	3.58	1.96	14	6.77	3.30	−1.13	[−1.95; −0.31]	0.8%
Pace−Schott et al., 2018	17	62.82	23.68	14	55.29	19.27	0.34	[−0.38; 1.05]	1.0%
Powers et al., 2009	12	15.42	13.39	12	27.08	21.37	−0.63	[−1.45; 0.19]	0.8%
Pyrkosch et al., 2018	36	21.16	8.81	33	19.79	9.89	0.15	[−0.33; 0.62]	1.5%
Raeder et al., 2019	20	26.05	14.35	23	32.17	19.72	−0.34	[−0.95; 0.26]	1.2%
Reinecke et al., 2020	17	2.10	17.20	14	21.30	30.80	−0.77	[−1.51; −0.03]	1.0%
Rodebaugh et al., 2013	18	55.89	20.46	16	65.85	20.19	−0.48	[−1.16; 0.21]	1.0%
Ross et al., 2016	12	7.45	4.81	14	13.88	5.19	−1.24	[−2.09; −0.39]	0.8%
Ross et al., 2021	6	40.33	10.97	5	49.01	17.87	−0.55	[−1.77; 0.67]	0.5%
Santa Ana et al., 2015	23	0.76	1.37	24	1.42	1.89	−0.39	[−0.97; 0.19]	1.2%
Siegmund et al., 2011	20	11.85	6.05	19	17.26	2.28	−1.15	[−1.83; −0.46]	1.0%
Simpson et al., 2010	15	11.90	2.35	15	13.75	3.60	−0.59	[−1.33; 0.14]	1.0%
Sippel et al., 2020	6	20.50	19.53	7	29.71	26.39	−0.36	[−1.47; 0.74]	0.6%
Smits et al., 2014	20	38.78	10.24	20	47.19	10.49	−0.80	[−1.44; −0.15]	1.1%
Smits et al., 2020a	114	60.56	23.34	38	65.88	25.16	−0.22	[−0.59; 0.15]	1.7%
Smits et al., 2020b	27	15.37	10.38	26	17.80	13.77	−0.20	[−0.74; 0.34]	1.3%
Soravia et al., 2014	11	43.30	11.28	11	53.50	17.58	−0.66	[−1.53; 0.20]	0.8%
Steudte−Schmiedgen et al., 2021	17	14.17	2.39	16	15.95	3.92	−0.54	[−1.24; 0.16]	1.0%
Storch et al., 2007	12	10.10	6.80	12	8.60	8.80	0.18	[−0.62; 0.99]	0.9%
Tart et al., 2013	15	29.73	25.67	14	35.55	25.18	−0.22	[−0.95; 0.51]	1.0%
Thierree et al., 2021	13	41.80	31.90	16	51.60	23.70	−0.34	[−1.08; 0.39]	1.0%
Tuerk et al., 2018	14	71.01	14.37	12	75.08	10.77	−0.31	[−1.08; 0.47]	0.9%
Weingarden et al., 2019	12	19.64	6.80	14	18.77	10.03	0.10	[−0.67; 0.87]	0.9%
Wilhelm et al., 2008	10	10.20	7.20	13	14.50	6.40	−0.61	[−1.46; 0.23]	0.8%
Witkiewitz et al., 2019	47	5.12	3.14	37	5.64	4.06	−0.14	[−0.58; 0.29]	1.5%
Wolfson et al., 2020	13	38.90	10.60	5	48.60	12.60	−0.83	[−1.91; 0.25]	0.6%
Zoellner et al., 2017	15	17.10	10.57	16	14.67	9.27	0.24	[−0.47; 0.95]	1.0%
**Random effects model**	**2264**			**2126**			**0.29**	**[0.39; 0.19]**	**100.0%**
**Prediction interval**								**[0.94; 0.37]**	

Heterogeneity: I^2 = 54%, $\square^2$ = 0.1054, p < 0.01

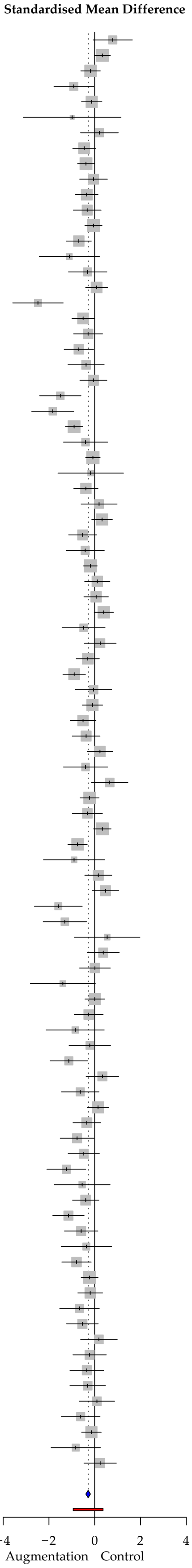

Supplementary Figure S4. Random effects meta-analysis results including only studies with high risk of bias. Main effect (k = 15 studies): Hedges' g = -0.19, 95% CI = (-0.45, 0.07); p = 0.1314.

Study	Augmentation N	Augmentation Mean	Augmentation SD	Control N	Control Mean	Control SD	SMD	95% CI	Weight
Alladin & Alibhai, 2007	42	17.50	8.50	42	21.30	7.20	−0.48	[−0.91; −0.04]	8.0%
Arntz et al., 2007	28	18.52	14.50	39	17.63	13.76	0.06	[−0.42; 0.55]	7.4%
Burton et al., 2019	10	48.70	12.70	10	53.60	15.40	−0.33	[−1.22; 0.55]	3.8%
Chien et al., 2015	43	11.58	4.51	46	12.74	4.19	−0.26	[−0.68; 0.15]	8.3%
Contalbrigo et al., 2017	12	0.35	0.19	10	0.67	0.43	−0.96	[−1.85; −0.06]	3.7%
Janse et al., 2020	190	0.41	0.40	178	0.41	0.39	0.01	[−0.19; 0.22]	11.1%
Lancaster et al., 2020	97	6.95	1.71	33	6.85	1.84	0.06	[−0.34; 0.45]	8.6%
Lass−Hennemann & Michael, 2014	30	4.60	0.34	30	5.27	0.47	−1.61	[−2.20; −1.02]	6.2%
Reitmaier et al., 2022	27	64.45	19.13	26	56.46	24.76	0.36	[−0.19; 0.90]	6.7%
Rubin et al., 2022	10	123.50	21.21	8	119.63	19.54	0.18	[−0.75; 1.11]	3.5%
Sherman et al., 2017	8	1.05	1.65	7	0.79	1.39	0.16	[−0.86; 1.18]	3.1%
Shiban et al., 2017	15	1.06	0.63	14	1.29	0.84	−0.30	[−1.04; 0.43]	4.8%
Smits et al., 2006	35	58.48	24.33	33	58.97	23.01	−0.02	[−0.50; 0.46]	7.5%
Soucy et al., 2021	203	6.26	4.78	231	6.22	5.05	0.01	[−0.18; 0.20]	11.3%
Vermes et al., 2020	21	0.36	0.60	21	0.51	0.44	−0.28	[−0.89; 0.33]	6.0%
**Random effects model**	**771**			**728**			**0.19**	**[0.45; 0.07]**	**100.0%**
**Prediction interval**								**[0.88; 0.49]**	

Heterogeneity: I^2 = 65%, □^{2} = 0.0887, p < 0.01

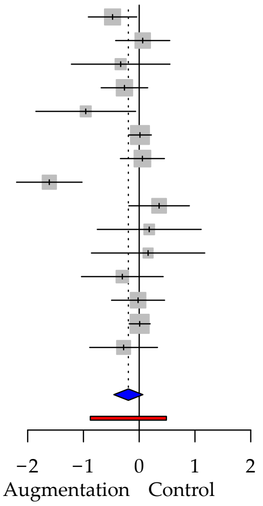

2.4. Results excluding psychological therapy combinations

Supplemental Figure S5. Psychological sub-study random effects meta-analysis results excluding six studies[31,43,64,65,90,97] identified as being "combinations" of psychotherapies rather than psychotherapy augmented with a psychological intervention. Main effect (k = 20 studies): Hedges' g = -0.16, 95% CI = (- 0.35, 0.03); p = 0.0886.

Study	Augmentation N	Augmentation Mean	Augmentation SD	Control N	Control Mean	Control SD	SMD	95% CI	Weight
Alladin & Alibhai, 2007	42	17.50	8.50	42	21.30	7.20	−0.48	[−0.91; −0.04]	5.9%
Arntz et al., 2007	28	18.52	14.50	39	17.63	13.76	0.06	[−0.42; 0.55]	5.3%
Baker et al., 2020	11	3.36	3.11	12	6.82	4.09	−0.91	[−1.78; −0.04]	2.6%
Bryant et al., 2005	30	11.30	9.98	33	16.58	12.50	−0.46	[−0.96; 0.04]	5.2%
Bryant et al., 2008	59	43.27	35.11	59	55.72	29.54	−0.38	[−0.75; −0.02]	6.8%
Cesa et al., 2013	27	45.00	13.90	39	49.97	14.30	−0.35	[−0.84; 0.15]	5.2%
Falkenstein et al., 2022	22	−0.33	0.75	8	0.96	1.05	−1.50	[−2.41; −0.60]	2.4%
Harb et al., 2019	40	3.20	1.56	38	2.70	1.52	0.32	[−0.13; 0.77]	5.8%
Harvey et al., 2016	22	19.41	11.69	20	25.45	10.83	−0.52	[−1.14; 0.09]	4.1%
Janse et al., 2020	190	0.41	0.40	178	0.41	0.39	0.01	[−0.19; 0.22]	8.8%
Lancaster et al., 2020	97	6.95	1.71	33	6.85	1.84	0.06	[−0.34; 0.45]	6.4%
Lazarov et al., 2018	25	48.37	24.25	25	60.05	20.70	−0.51	[−1.07; 0.05]	4.6%
Lee et al., 2020	22	−9.86	6.13	20	−7.94	3.37	−0.38	[−0.99; 0.24]	4.2%
Maples−Keller et al., 2017	45	65.55	49.88	44	76.57	47.46	−0.22	[−0.64; 0.19]	6.1%
McEvoy et al., 2020	51	36.91	2.17	54	36.24	1.82	0.33	[−0.05; 0.72]	6.5%
Moshier & Otto, 2017	21	23.10	10.80	13	18.60	13.30	0.37	[−0.33; 1.07]	3.5%
Reitmaier et al., 2022	27	64.45	19.13	26	56.46	24.76	0.36	[−0.19; 0.90]	4.8%
Rubin et al., 2022	10	123.50	21.21	8	119.63	19.54	0.18	[−0.75; 1.11]	2.3%
Smits et al., 2006	35	58.48	24.33	33	58.97	23.01	−0.02	[−0.50; 0.46]	5.4%
Vermes et al., 2020	21	0.36	0.60	21	0.51	0.44	−0.28	[−0.89; 0.33]	4.2%
**Random effects model**	**825**			**745**			**0.16**	**[0.35; 0.03]**	**100.0%**
**Prediction interval**								**[0.73; 0.41]**	

Heterogeneity: I^2 = 54%, □^{2} = 0.0661, p < 0.01

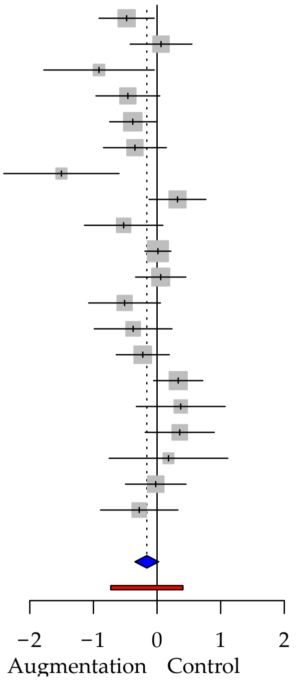

Supplemental Figure S6. Random effects meta-analysis results excluding the six psychological combination studies. Main effect (k = 102 studies): Hedges' g = -0.28, 95% CI = (-0.37, -0.18); $p < 0.0001$.

Study	Augmentation N	Augmentation Mean	Augmentation SD	Control N	Control Mean	Control SD	Standardised Mean Difference	SMD	95% CI	Weight
Acheson et al., 2015	10	63.86	24.03	13	44.66	22.95		0.79	[−0.07; 1.65]	0.7%
Alladin & Alibhai, 2007	42	17.50	8.50	42	21.30	7.20		−0.48	[−0.91; −0.04]	1.4%
Andersson et al., 2015	64	13.86	6.50	64	11.77	5.95		0.33	[−0.02; 0.68]	1.6%
Arntz et al., 2007	28	18.52	14.50	39	17.63	13.76		0.06	[−0.42; 0.55]	1.3%
Aust et al., 2022	43	14.40	6.90	42	15.70	7.30		−0.18	[−0.61; 0.24]	1.4%
Baker et al., 2020	11	3.36	3.11	12	6.82	4.09		−0.91	[−1.78; −0.04]	0.7%
Bischoff et al., 2018	39	12.00	7.60	38	13.00	7.50		−0.13	[−0.58; 0.32]	1.4%
Bouso et al., 2008	3	28.30	10.48	2	40.00	1.41		−0.98	[−3.12; 1.15]	0.2%
Brooks et al., 2021	10	−47.70	8.60	13	−49.80	10.70		0.21	[−0.62; 1.03]	0.8%
Bryant et al., 2005	30	11.30	9.98	33	16.58	12.50		−0.46	[−0.96; 0.04]	1.3%
Bryant et al., 2008	59	43.27	35.11	59	55.72	29.54		−0.38	[−0.75; −0.02]	1.5%
Buchanan et al., 2021	22	125.30	20.30	20	126.30	17.40		−0.05	[−0.66; 0.55]	1.1%
Burton et al., 2019	10	48.70	12.70	10	53.60	15.40		−0.33	[−1.22; 0.55]	0.7%
Cesa et al., 2013	27	45.00	13.90	39	49.97	14.30		−0.35	[−0.84; 0.15]	1.3%
Chien et al., 2015	43	11.58	4.51	46	12.74	4.19		−0.26	[−0.68; 0.15]	1.4%
Cobb et al., 2021	23	7.83	11.52	19	12.74	17.75		−0.33	[−0.94; 0.28]	1.1%
Contalbrigo et al., 2017	12	0.35	0.19	10	0.67	0.43		−0.96	[−1.85; −0.06]	0.7%
Craske et al., 2019	39	0.20	0.56	21	0.58	0.50		−0.69	[−1.24; −0.15]	1.2%
Danforth et al., 2018	8	46.40	15.20	4	64.00	13.30		−1.11	[−2.42; 0.21]	0.4%
Davis et al., 2014	11	0.11	0.70	11	0.35	0.80		−0.31	[−1.15; 0.53]	0.8%
Davis et al., 2017	26	33.54	8.41	47	32.59	12.61		0.08	[−0.40; 0.56]	1.3%
Davis et al., 2021	13	8.50	5.70	11	23.50	6.00		−2.48	[−3.59; −1.37]	0.5%
De Kleine et al., 2012	33	34.33	37.11	34	53.65	38.19		−0.51	[−0.99; −0.02]	1.3%
de Leeuw et al., 2017	19	16.60	4.20	20	18.00	5.20		−0.29	[−0.92; 0.34]	1.0%
De Quervain et al., 2011	20	30.40	13.86	20	40.20	13.86		−0.69	[−1.33; −0.05]	1.0%
Difede et al., 2014	13	32.38	28.55	12	42.17	20.75		−0.38	[−1.17; 0.42]	0.8%
Diminich et al., 2020	22	11.91	5.26	22	12.18	3.79		−0.06	[−0.65; 0.53]	1.1%
Falkenstein et al., 2022	22	−0.33	0.75	8	0.96	1.05		−1.50	[−2.41; −0.60]	0.7%
Fayaz et al., 2022	15	18.00	5.00	12	26.00	3.00		−1.83	[−2.75; −0.91]	0.7%
Feng et al., 2019	60	34.30	12.60	60	46.00	13.20		−0.90	[−1.28; −0.52]	1.5%
Flanagan et al., 2018	8	13.00	12.74	9	17.71	9.62		−0.40	[−1.36; 0.56]	0.6%
Fryml et al., 2019	5	36.00	36.71	3	43.30	35.23		−0.18	[−1.61; 1.26]	0.3%
Guastella et al., 2008	28	89.52	22.63	28	99.30	27.26		−0.38	[−0.91; 0.14]	1.2%
Guastella et al., 2009	12	96.57	20.19	13	91.41	30.59		0.19	[−0.60; 0.98]	0.8%
Harb et al., 2019	40	3.20	1.56	38	2.70	1.52		0.32	[−0.13; 0.77]	1.4%
Harvey et al., 2016	22	19.41	11.69	20	25.45	10.83		−0.52	[−1.14; 0.09]	1.1%
Hofmann et al., 2006	10	81.74	27.01	13	96.21	37.74		−0.42	[−1.25; 0.42]	0.8%
Hofmann et al., 2013	87	2.68	1.41	82	2.95	1.43		−0.19	[−0.49; 0.11]	1.6%
Hofmeijer−Sevink et al., 2017	38	2.15	2.32	19	1.88	2.48		0.11	[−0.44; 0.66]	1.2%
Hutschemaekers et al., 2021	27	38.22	21.22	27	36.88	20.84		0.06	[−0.47; 0.60]	1.2%
Isserles et al., 2013	9	61.00	26.40	9	76.00	32.10		−0.49	[−1.43; 0.45]	0.7%
Isserles et al., 2021	40	27.37	12.59	51	22.30	12.71		0.40	[−0.02; 0.82]	1.4%
Janse et al., 2020	190	0.41	0.40	178	0.41	0.39		0.01	[−0.19; 0.22]	1.8%
Kamboj et al., 2012	16	63.11	25.83	16	56.38	28.29		0.24	[−0.45; 0.94]	0.9%
Kozel et al., 2018	31	31.11	22.49	30	38.29	24.43		−0.30	[−0.81; 0.20]	1.3%
Krupitsky et al., 2002	35	3.97	5.04	35	15.06	16.54		−0.90	[−1.39; −0.40]	1.3%
Kushner et al., 2007	14	10.90	4.70	11	11.20	6.80		−0.05	[−0.84; 0.74]	0.8%
Kwee et al., 2022	39	35.38	18.24	39	37.13	17.26		−0.10	[−0.54; 0.35]	1.4%
Lancaster et al., 2020	97	6.95	1.71	33	6.85	1.84		0.06	[−0.34; 0.45]	1.5%
Lass−Hennemann & Michael, 2014	30	4.60	0.34	30	5.27	0.47		−1.61	[−2.20; −1.02]	1.1%
Lazarov et al., 2018	25	48.37	24.25	25	60.05	20.70		−0.51	[−1.07; 0.05]	1.1%
Lee et al., 2020	22	−9.86	6.13	20	−7.94	3.37		−0.38	[−0.99; 0.24]	1.1%
Lehrner et al., 2021	24	52.89	16.31	26	48.94	17.49		0.23	[−0.33; 0.79]	1.2%
Leuchter et al., 2022	8	71.13	21.85	9	80.22	21.31		−0.40	[−1.36; 0.56]	0.6%
Litz et al., 2012	13	72.33	28.63	13	53.73	26.22		0.66	[−0.14; 1.45]	0.8%
Maples−Keller et al., 2017	45	65.55	49.88	44	76.57	47.46		−0.22	[−0.64; 0.19]	1.4%
McEvoy et al., 2020	51	36.91	2.17	54	36.24	1.82		0.33	[−0.05; 0.72]	1.5%
Meyer et al., 2022	5	2.21	3.35	5	5.62	3.47		−0.90	[−2.24; 0.43]	0.4%
Meyerbroeker et al., 2012	20	26.87	19.64	25	23.96	17.10		0.16	[−0.43; 0.75]	1.1%
Meyerbroker et al., 2018	37	36.71	15.41	17	29.18	16.45		0.47	[−0.11; 1.05]	1.1%
Mithoefer et al., 2011	12	29.30	22.52	8	66.80	22.63		−1.59	[−2.64; −0.54]	0.6%
Mithoefer et al., 2018	19	37.49	30.12	7	76.00	23.40		−1.30	[−2.25; −0.36]	0.6%
Morissette et al., 2008	4	5.15	1.05	4	4.40	1.33		0.54	[−0.89; 1.98]	0.3%
Moshier & Otto, 2017	21	23.10	10.80	13	18.60	13.30		0.37	[−0.33; 1.07]	0.9%
Nations et al., 2012	24	6.64	3.78	13	6.60	4.29		0.01	[−0.67; 0.68]	1.0%
Nejati et al., 2017	4	12.25	5.62	7	22.99	7.66		−1.39	[−2.81; 0.03]	0.3%
Nicholas et al., 2022	42	3.24	3.36	40	3.23	3.65		0.00	[−0.43; 0.44]	1.4%
Nord et al., 2019	20	12.65	6.91	19	14.37	5.81		−0.26	[−0.89; 0.37]	1.0%
Oehen et al., 2013	8	50.80	19.70	4	66.50	7.60		−0.85	[−2.12; 0.42]	0.4%
Ot'alora G et al., 2018	22	66.74	30.78	6	73.30	24.50		−0.21	[−1.12; 0.69]	0.7%
Otto et al., 2010	13	3.58	1.96	14	6.77	3.30		−1.13	[−1.95; −0.31]	0.8%
Pace−Schott et al., 2018	17	62.82	23.68	14	55.29	19.27		0.34	[−0.38; 1.05]	0.9%
Powers et al., 2009	12	15.42	13.39	12	27.08	21.37		−0.63	[−1.45; 0.19]	0.8%
Pyrkosch et al., 2018	36	21.16	8.81	33	19.79	9.89		0.15	[−0.33; 0.62]	1.3%
Raeder et al., 2019	20	26.05	14.35	23	32.17	19.72		−0.34	[−0.95; 0.26]	1.1%
Reinecke et al., 2020	17	2.10	17.20	14	21.30	30.80		−0.77	[−1.51; −0.03]	0.9%
Reitmaier et al., 2022	27	64.45	19.13	26	56.46	24.76		0.36	[−0.19; 0.90]	1.2%
Rodebaugh et al., 2013	18	55.89	20.46	16	65.85	20.19		−0.48	[−1.16; 0.21]	1.0%
Ross et al., 2016	12	7.45	4.81	14	13.88	5.19		−1.24	[−2.09; −0.39]	0.7%
Ross et al., 2021	6	40.33	10.97	5	49.01	17.87		−0.55	[−1.77; 0.67]	0.4%
Rubin et al., 2022	10	123.50	21.21	8	119.63	19.54		0.18	[−0.75; 1.11]	0.7%
Santa Ana et al., 2015	23	0.76	1.37	24	1.42	1.89		−0.39	[−0.97; 0.19]	1.1%
Sherman et al., 2017	8	1.05	1.65	7	0.79	1.39		0.16	[−0.86; 1.18]	0.6%
Shiban et al., 2017	15	1.06	0.63	14	1.29	0.84		−0.30	[−1.04; 0.43]	0.9%
Siegmund et al., 2011	20	11.85	6.05	19	17.26	2.28		−1.15	[−1.83; −0.46]	1.0%
Sippel et al., 2020	6	20.50	19.53	7	29.71	26.39		−0.36	[−1.47; 0.74]	0.5%
Smits et al., 2006	35	58.48	24.33	33	58.97	23.01		−0.02	[−0.50; 0.46]	1.3%
Smits et al., 2014	20	38.78	10.24	20	47.19	10.49		−0.80	[−1.44; −0.15]	1.0%
Smits et al., 2020a	114	60.56	23.34	38	65.88	25.16		−0.22	[−0.59; 0.15]	1.5%
Smits et al., 2020b	27	15.37	10.38	26	17.80	13.77		−0.20	[−0.74; 0.34]	1.2%
Soravia et al., 2014	11	43.30	11.28	11	53.50	17.58		−0.66	[−1.53; 0.20]	0.7%
Steudte−Schmiedgen et al., 2021	17	14.17	2.39	16	15.95	3.92		−0.54	[−1.24; 0.16]	0.9%
Storch et al., 2007	12	10.10	6.80	12	8.60	8.80		0.18	[−0.62; 0.99]	0.8%
Tart et al., 2013	15	29.73	25.67	14	35.55	25.18		−0.22	[−0.95; 0.51]	0.9%
Thierree et al., 2021	13	41.80	31.90	16	51.60	23.70		−0.34	[−1.08; 0.39]	0.9%
Tuerk et al., 2018	14	71.01	14.37	12	75.08	10.77		−0.31	[−1.08; 0.47]	0.8%
Vermes et al., 2020	21	0.36	0.60	21	0.51	0.44		−0.28	[−0.89; 0.33]	1.1%
Weingarden et al., 2019	12	19.64	6.80	14	18.77	10.03		0.10	[−0.67; 0.87]	0.8%
Wilhelm et al., 2008	10	10.20	7.20	13	14.50	6.40		−0.61	[−1.46; 0.23]	0.7%
Witkiewitz et al., 2019	47	5.12	3.14	37	5.64	4.06		−0.14	[−0.58; 0.29]	1.4%
Wolfson et al., 2020	13	38.90	10.60	5	48.60	12.60		−0.83	[−1.91; 0.25]	0.5%
Zoellner et al., 2017	15	17.10	10.57	16	14.67	9.27		0.24	[−0.47; 0.95]	0.9%
**Random effects model**	**2637**			**2380**				**0.28**	**[0.37; 0.18]**	**100.0%**
**Prediction interval**									**[0.95; 0.39]**	

Heterogeneity: I^2 = 56%, □^{2} = 0.1120, p < 0.01

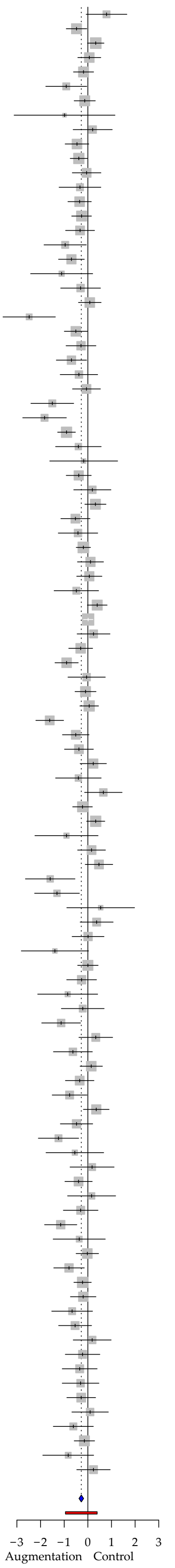

2.5. Results excluding non-randomised studies

Supplemental Figure S7. Random effects meta-analysis results excluding two non-randomised studies[27,32]. Main effect (k = 106 studies): Hedges' g = -0.27, 95% CI = (-0.36, -0.17); p < 0.0001.

	Augmentation			Control					
**Study**	**N**	**Mean**	**SD**	**N**	**Mean**	**SD**	**SMD**	**95% CI**	**Weight**
Acheson et al., 2015	10	63.86	24.03	13	44.66	22.95	0.79	[−0.07; 1.65]	0.7%
Alladin & Alibhai, 2007	42	17.50	8.50	42	21.30	7.20	−0.48	[−0.91; −0.04]	1.3%
Andersson et al., 2015	64	13.86	6.50	64	11.77	5.95	0.33	[−0.02; 0.68]	1.5%
Arntz et al., 2007	28	18.52	14.50	39	17.63	13.76	0.06	[−0.42; 0.55]	1.2%
Aust et al., 2022	43	14.40	6.90	42	15.70	7.30	−0.18	[−0.61; 0.24]	1.3%
Baker et al., 2020	11	3.36	3.11	12	6.82	4.09	−0.91	[−1.78; −0.04]	0.7%
Bischoff et al., 2018	39	12.00	7.60	38	13.00	7.50	−0.13	[−0.58; 0.32]	1.3%
Bouso et al., 2008	3	28.30	10.48	2	40.00	1.41	−0.98	[−3.12; 1.15]	0.2%
Brooks et al., 2021	10	−47.70	8.60	13	−49.80	10.70	0.21	[−0.62; 1.03]	0.7%
Bryant et al., 2005	30	11.30	9.98	33	16.58	12.50	−0.46	[−0.96; 0.04]	1.2%
Bryant et al., 2008	59	43.27	35.11	59	55.72	29.54	−0.38	[−0.75; −0.02]	1.5%
Buchanan et al., 2021	22	125.30	20.30	20	126.30	17.40	−0.05	[−0.66; 0.55]	1.0%
Cesa et al., 2013	27	45.00	13.90	39	49.97	14.30	−0.35	[−0.84; 0.15]	1.2%
Chien et al., 2015	43	11.58	4.51	46	12.74	4.19	−0.26	[−0.68; 0.15]	1.3%
Cobb et al., 2021	23	7.83	11.52	19	12.74	17.75	−0.33	[−0.94; 0.28]	1.0%
Coffey et al., 2016	40	20.49	17.87	86	21.54	19.57	−0.05	[−0.43; 0.32]	1.4%
Craske et al., 2019	39	0.20	0.56	21	0.58	0.50	−0.69	[−1.24; −0.15]	1.1%
Danforth et al., 2018	8	46.40	15.20	4	64.00	13.30	−1.11	[−2.42; 0.21]	0.4%
Davis et al., 2014	11	0.11	0.70	11	0.35	0.80	−0.31	[−1.15; 0.53]	0.7%
Davis et al., 2017	26	33.54	8.41	47	32.59	12.61	0.08	[−0.40; 0.56]	1.2%
Davis et al., 2020	13	8.50	5.70	11	23.50	6.00	−2.48	[−3.59; −1.37]	0.5%
De Kleine et al., 2012	33	34.33	37.11	34	53.65	38.19	−0.51	[−0.99; −0.02]	1.2%
de Leeuw et al., 2017	19	16.60	4.20	20	18.00	5.20	−0.29	[−0.92; 0.34]	1.0%
De Quervain et al., 2011	20	30.40	13.86	20	40.20	13.86	−0.69	[−1.33; −0.05]	1.0%
Difede et al., 2014	13	32.38	28.55	12	42.17	20.75	−0.38	[−1.17; 0.42]	0.7%
Diminich et al., 2020	22	11.91	5.26	22	12.18	3.79	−0.06	[−0.65; 0.53]	1.0%
Falkenstein et al., 2022	22	−0.33	0.75	8	0.96	1.05	−1.50	[−2.41; −0.60]	0.6%
Fayaz et al., 2022	15	18.00	5.00	12	26.00	3.00	−1.83	[−2.75; −0.91]	0.6%
Feng et al., 2019	60	34.30	12.60	60	46.00	13.20	−0.90	[−1.28; −0.52]	1.4%
Flanagan et al., 2018	8	13.00	12.74	9	17.71	9.62	−0.40	[−1.36; 0.56]	0.6%
Foa et al., 2005	74	16.80	13.20	79	17.90	14.50	−0.08	[−0.40; 0.24]	1.6%
Fryml et al., 2019	5	36.00	36.71	3	43.30	35.23	−0.18	[−1.61; 1.26]	0.3%
Guastella et al., 2008	28	89.52	22.63	28	99.30	27.26	−0.38	[−0.91; 0.14]	1.1%
Guastella et al., 2009	12	96.57	20.19	13	91.41	30.59	0.19	[−0.60; 0.98]	0.8%
Harb et al., 2019	40	3.20	1.56	38	2.70	1.52	0.32	[−0.13; 0.77]	1.3%
Harvey et al., 2016	22	19.41	11.69	20	25.45	10.83	−0.52	[−1.14; 0.09]	1.0%
Hofmann et al., 2006	10	81.74	27.01	13	96.21	37.74	−0.42	[−1.25; 0.42]	0.7%
Hofmann et al., 2013	87	2.68	1.41	82	2.95	1.43	−0.19	[−0.49; 0.11]	1.6%
Hofmeijer−Sevink et al., 2017	38	2.15	2.32	19	1.88	2.48	0.11	[−0.44; 0.66]	1.1%
Hutschemaekers et al., 2021	27	38.22	21.22	27	36.88	20.84	0.06	[−0.47; 0.60]	1.1%
Isserles et al., 2021	40	27.37	12.59	51	22.30	12.71	0.40	[−0.02; 0.82]	1.3%
Isserles et al., 2013	9	61.00	26.40	9	76.00	32.10	−0.49	[−1.43; 0.45]	0.6%
Janse et al., 2020	190	0.41	0.40	178	0.41	0.39	0.01	[−0.19; 0.22]	1.8%
Kamboj et al., 2012	16	63.11	25.83	16	56.38	28.29	0.24	[−0.45; 0.94]	0.9%
Kozel et al., 2018	31	31.11	22.49	30	38.29	24.43	−0.30	[−0.81; 0.20]	1.2%
Krupitsky et al., 2002	35	3.97	5.04	35	15.06	16.54	−0.90	[−1.39; −0.40]	1.2%
Kushner et al., 2007	14	10.90	4.70	11	11.20	6.80	−0.05	[−0.84; 0.74]	0.7%
Kwee et al., 2022	39	35.38	18.24	39	37.13	17.26	−0.10	[−0.54; 0.35]	1.3%
Lancaster et al., 2020	97	6.95	1.71	33	6.85	1.84	0.06	[−0.34; 0.45]	1.4%
Lass−Hennemann & Michael, 2014	30	4.60	0.34	30	5.27	0.47	−1.61	[−2.20; −1.02]	1.0%
Lazarov et al., 2018	25	48.37	24.25	25	60.05	20.70	−0.51	[−1.07; 0.05]	1.1%
Lee et al., 2020	22	−9.86	6.13	20	−7.94	3.37	−0.38	[−0.99; 0.24]	1.0%
Lehrner et al., 2021	24	52.89	16.31	26	48.94	17.49	0.23	[−0.33; 0.79]	1.1%
Leuchter et al., 2022	8	71.13	21.85	9	80.22	21.31	−0.40	[−1.36; 0.56]	0.6%
Litz et al., 2012	13	72.33	28.63	13	53.73	26.22	0.66	[−0.14; 1.45]	0.7%
Maples−Keller et al., 2017	45	65.55	49.88	44	76.57	47.46	−0.22	[−0.64; 0.19]	1.3%
Marker et al., 2020	18	2.43	1.28	18	2.85	1.28	−0.32	[−0.98; 0.34]	0.9%
McEvoy et al., 2020	51	36.91	2.17	54	36.24	1.82	0.33	[−0.05; 0.72]	1.4%
Meyer et al., 2010	48	8.15	5.43	45	13.90	9.39	−0.75	[−1.17; −0.33]	1.3%
Meyer et al., 2022	5	2.21	3.35	5	5.62	3.47	−0.90	[−2.24; 0.43]	0.3%
Meyerbroeker et al., 2012	20	26.87	19.64	25	23.96	17.10	0.16	[−0.43; 0.75]	1.0%
Meyerbroker et al., 2018	37	36.71	15.41	17	29.18	16.45	0.47	[−0.11; 1.05]	1.0%
Mithoefer et al., 2011	12	29.30	22.52	8	66.80	22.63	−1.59	[−2.64; −0.54]	0.5%
Mithoefer et al., 2018	19	37.49	30.12	7	76.00	23.40	−1.30	[−2.25; −0.36]	0.6%
Morissette et al., 2008	4	5.15	1.05	4	4.40	1.33	0.54	[−0.89; 1.98]	0.3%
Moshier & Otto, 2017	21	23.10	10.80	13	18.60	13.30	0.37	[−0.33; 1.07]	0.9%
Nations et al., 2012	24	6.64	3.78	13	6.60	4.29	0.01	[−0.67; 0.68]	0.9%
Nejati et al., 2017	4	12.25	5.62	7	22.99	7.66	−1.39	[−2.81; 0.03]	0.3%
Nicholas et al., 2022	42	3.24	3.36	40	3.23	3.65	0.00	[−0.43; 0.44]	1.3%
Nord et al., 2019	20	12.65	6.91	19	14.37	5.81	−0.26	[−0.89; 0.37]	1.0%
Oehen et al., 2013	8	50.80	19.70	4	66.50	7.60	−0.85	[−2.12; 0.42]	0.4%
Ot'alora G et al., 2018	22	66.74	30.78	6	73.30	24.50	−0.21	[−1.12; 0.69]	0.6%
Otto et al., 2010	13	3.58	1.96	14	6.77	3.30	−1.13	[−1.95; −0.31]	0.7%
Pace−Schott et al., 2018	17	62.82	23.68	14	55.29	19.27	0.34	[−0.38; 1.05]	0.8%
Powers et al., 2009	12	15.42	13.39	12	27.08	21.37	−0.63	[−1.45; 0.19]	0.7%
Pyrkosch et al., 2018	36	21.16	8.81	33	19.79	9.89	0.15	[−0.33; 0.62]	1.2%
Raeder et al., 2019	20	26.05	14.35	23	32.17	19.72	−0.34	[−0.95; 0.26]	1.0%
Reinecke et al., 2020	17	2.10	17.20	14	21.30	30.80	−0.77	[−1.51; −0.03]	0.8%
Reitmaier et al., 2022	27	64.45	19.13	26	56.46	24.76	0.36	[−0.19; 0.90]	1.1%
Rodebaugh et al., 2013	18	55.89	20.46	16	65.85	20.19	−0.48	[−1.16; 0.21]	0.9%
Ross et al., 2016	12	7.45	4.81	14	13.88	5.19	−1.24	[−2.09; −0.39]	0.7%
Ross et al., 2021	6	40.33	10.97	5	49.01	17.87	−0.55	[−1.77; 0.67]	0.4%
Rubin et al., 2022	10	123.50	21.21	8	119.63	19.54	0.18	[−0.75; 1.11]	0.6%
Santa Ana et al., 2015	23	0.76	1.37	24	1.42	1.89	−0.39	[−0.97; 0.19]	1.0%
Sherman et al., 2017	8	1.05	1.65	7	0.79	1.39	0.16	[−0.86; 1.18]	0.5%
Shiban et al., 2017	15	1.06	0.63	14	1.29	0.84	−0.30	[−1.04; 0.43]	0.8%
Siegmund et al., 2011	20	11.85	6.05	19	17.26	2.28	−1.15	[−1.83; −0.46]	0.9%
Simpson et al., 2010	15	11.90	2.35	15	13.75	3.60	−0.59	[−1.33; 0.14]	0.8%
Sippel et al., 2020	6	20.50	19.53	7	29.71	26.39	−0.36	[−1.47; 0.74]	0.5%
Smits et al., 2006	35	58.48	24.33	33	58.97	23.01	−0.02	[−0.50; 0.46]	1.2%
Smits et al., 2014	20	38.78	10.24	20	47.19	10.49	−0.80	[−1.44; −0.15]	0.9%
Smits et al., 2020a	114	60.56	23.34	38	65.88	25.16	−0.22	[−0.59; 0.15]	1.4%
Smits et al., 2020b	27	15.37	10.38	26	17.80	13.77	−0.20	[−0.74; 0.34]	1.1%
Soravia et al., 2014	11	43.30	11.28	11	53.50	17.58	−0.66	[−1.53; 0.20]	0.7%
Soucy et al., 2021	203	6.26	4.78	231	6.22	5.05	0.01	[−0.18; 0.20]	1.8%
Steudte−Schmiedgen et al., 2021	17	14.17	2.39	16	15.95	3.92	−0.54	[−1.24; 0.16]	0.9%
Storch et al., 2007	12	10.10	6.80	12	8.60	8.80	0.18	[−0.62; 0.99]	0.7%
Tart et al., 2013	15	29.73	25.67	14	35.55	25.18	−0.22	[−0.95; 0.51]	0.8%
Thierree et al., 2021	13	41.80	31.90	16	51.60	23.70	−0.34	[−1.08; 0.39]	0.8%
Tuerk et al., 2018	14	71.01	14.37	12	75.08	10.77	−0.31	[−1.08; 0.47]	0.8%
Vermes et al., 2020	21	0.36	0.60	21	0.51	0.44	−0.28	[−0.89; 0.33]	1.0%
Weingarden et al., 2019	12	19.64	6.80	14	18.77	10.03	0.10	[−0.67; 0.87]	0.8%
Wilhelm et al., 2008	10	10.20	7.20	13	14.50	6.40	−0.61	[−1.46; 0.23]	0.7%
Witkiewitz et al., 2019	47	5.12	3.14	37	5.64	4.06	−0.14	[−0.58; 0.29]	1.3%
Wolfson et al., 2020	13	38.90	10.60	5	48.60	12.60	−0.83	[−1.91; 0.25]	0.5%
Zoellner et al., 2017	15	17.10	10.57	16	14.67	9.27	0.24	[−0.47; 0.95]	0.9%
**Random effects model**	**3013**			**2834**			**0.27**	**[0.36; 0.17]**	**100.0%**
**Prediction interval**								**[0.90; 0.37]**	

Heterogeneity: I^2 = 57%, □^{2} = 0.1011, p < 0.01

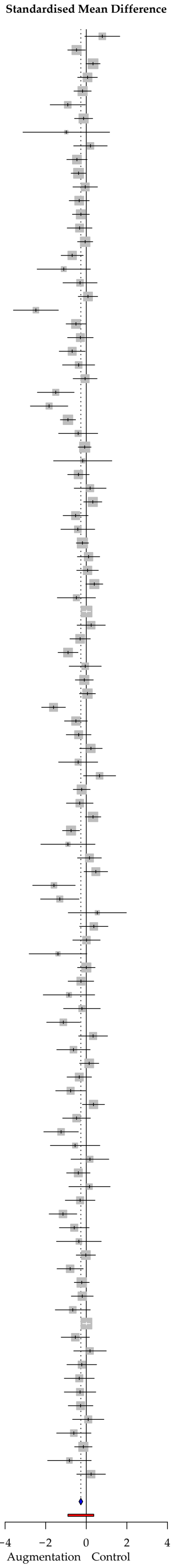

2.6. Results excluding studies with non-therapeutic controls

Supplemental Figure S8. Random effects meta-analysis results excluding two studies in which the control group did not receive psychological therapy (either waitlist[3] or occupational therapy[29]). Main effect (k = 106 studies): Hedges' g = -0.27, 95% CI = (-0.36, -0.17); $p < 0.0001$.

Study	Augmentation N	Augmentation Mean	Augmentation SD	Control N	Control Mean	Control SD	SMD	95% CI	Weight
Acheson et al., 2015	10	63.86	24.03	13	44.66	22.95	0.79	[−0.07; 1.65]	0.7%
Alladin & Alibhai, 2007	42	17.50	8.50	42	21.30	7.20	−0.48	[−0.91; −0.04]	1.3%
Andersson et al., 2015	64	13.86	6.50	64	11.77	5.95	0.33	[−0.02; 0.68]	1.5%
Arntz et al., 2007	28	18.52	14.50	39	17.63	13.76	0.06	[−0.42; 0.55]	1.2%
Aust et al., 2022	43	14.40	6.90	42	15.70	7.30	−0.18	[−0.61; 0.24]	1.4%
Baker et al., 2020	11	3.36	3.11	12	6.82	4.09	−0.91	[−1.78; −0.04]	0.7%
Bischoff et al., 2018	39	12.00	7.60	38	13.00	7.50	−0.13	[−0.58; 0.32]	1.3%
Bouso et al., 2008	3	28.30	10.48	2	40.00	1.41	−0.98	[−3.12; 1.15]	0.1%
Brooks et al., 2021	10	−47.70	8.60	13	−49.80	10.70	0.21	[−0.62; 1.03]	0.7%
Bryant et al., 2005	30	11.30	9.98	33	16.58	12.50	−0.46	[−0.96; 0.04]	1.2%
Bryant et al., 2008	59	43.27	35.11	59	55.72	29.54	−0.38	[−0.75; −0.02]	1.5%
Buchanan et al., 2021	22	125.30	20.30	20	126.30	17.40	−0.05	[−0.66; 0.55]	1.0%
Burton et al., 2019	10	48.70	12.70	10	53.60	15.40	−0.33	[−1.22; 0.55]	0.6%
Cesa et al., 2013	27	45.00	13.90	39	49.97	14.30	−0.35	[−0.84; 0.15]	1.2%
Cobb et al., 2021	23	7.83	11.52	19	12.74	17.75	−0.33	[−0.94; 0.28]	1.0%
Coffey et al., 2016	40	20.49	17.87	86	21.54	19.57	−0.05	[−0.43; 0.32]	1.5%
Contalbrigo et al., 2017	12	0.35	0.19	10	0.67	0.43	−0.96	[−1.85; −0.06]	0.6%
Craske et al., 2019	39	0.20	0.56	21	0.58	0.50	−0.69	[−1.24; −0.15]	1.1%
Danforth et al., 2018	8	46.40	15.20	4	64.00	13.30	−1.11	[−2.42; 0.21]	0.3%
Davis et al., 2014	11	0.11	0.70	11	0.35	0.80	−0.31	[−1.15; 0.53]	0.7%
Davis et al., 2017	26	33.54	8.41	47	32.59	12.61	0.08	[−0.40; 0.56]	1.2%
De Kleine et al., 2012	33	34.33	37.11	34	53.65	38.19	−0.51	[−0.99; −0.02]	1.2%
de Leeuw et al., 2017	19	16.60	4.20	20	18.00	5.20	−0.29	[−0.92; 0.34]	1.0%
De Quervain et al., 2011	20	30.40	13.86	20	40.20	13.86	−0.69	[−1.33; −0.05]	0.9%
Difede et al., 2014	13	32.38	28.55	12	42.17	20.75	−0.38	[−1.17; 0.42]	0.7%
Diminich et al., 2020	22	11.91	5.26	22	12.18	3.79	−0.06	[−0.65; 0.53]	1.0%
Falkenstein et al., 2022	22	−0.33	0.75	8	0.96	1.05	−1.50	[−2.41; −0.60]	0.6%
Fayaz et al., 2022	15	18.00	5.00	12	26.00	3.00	−1.83	[−2.75; −0.91]	0.6%
Feng et al., 2019	60	34.30	12.60	60	46.00	13.20	−0.90	[−1.28; −0.52]	1.5%
Flanagan et al., 2018	8	13.00	12.74	9	17.71	9.62	−0.40	[−1.36; 0.56]	0.6%
Foa et al., 2005	74	16.80	13.20	79	17.90	14.50	−0.08	[−0.40; 0.24]	1.6%
Fryml et al., 2019	5	36.00	36.71	3	43.30	35.23	−0.18	[−1.61; 1.26]	0.3%
Guastella et al., 2008	28	89.52	22.63	28	99.30	27.26	−0.38	[−0.91; 0.14]	1.1%
Guastella et al., 2009	12	96.57	20.19	13	91.41	30.59	0.19	[−0.60; 0.98]	0.7%
Harb et al., 2019	40	3.20	1.56	38	2.70	1.52	0.32	[−0.13; 0.77]	1.3%
Harvey et al., 2016	22	19.41	11.69	20	25.45	10.83	−0.52	[−1.14; 0.09]	1.0%
Hofmann et al., 2006	10	81.74	27.01	13	96.21	37.74	−0.42	[−1.25; 0.42]	0.7%
Hofmann et al., 2013	87	2.68	1.41	82	2.95	1.43	−0.19	[−0.49; 0.11]	1.6%
Hofmeijer−Sevink et al., 2017	38	2.15	2.32	19	1.88	2.48	0.11	[−0.44; 0.66]	1.1%
Hutschemaekers et al., 2021	27	38.22	21.22	27	36.88	20.84	0.06	[−0.47; 0.60]	1.1%
Isserles et al., 2021	40	27.37	12.59	51	22.30	12.71	0.40	[−0.02; 0.82]	1.4%
Isserles et al., 2013	9	61.00	26.40	9	76.00	32.10	−0.49	[−1.43; 0.45]	0.6%
Janse et al., 2020	190	0.41	0.40	178	0.41	0.39	0.01	[−0.19; 0.22]	1.8%
Kamboj et al., 2012	16	63.11	25.83	16	56.38	28.29	0.24	[−0.45; 0.94]	0.9%
Kozel et al., 2018	31	31.11	22.49	30	38.29	24.43	−0.30	[−0.81; 0.20]	1.2%
Krupitsky et al., 2002	35	3.97	5.04	35	15.06	16.54	−0.90	[−1.39; −0.40]	1.2%
Kushner et al., 2007	14	10.90	4.70	11	11.20	6.80	−0.05	[−0.84; 0.74]	0.7%
Kwee et al., 2022	39	35.38	18.24	39	37.13	17.26	−0.10	[−0.54; 0.35]	1.3%
Lancaster et al., 2020	97	6.95	1.71	33	6.85	1.84	0.06	[−0.34; 0.45]	1.4%
Lass−Hennemann & Michael, 2014	30	4.60	0.34	30	5.27	0.47	−1.61	[−2.20; −1.02]	1.0%
Lazarov et al., 2018	25	48.37	24.25	25	60.05	20.70	−0.51	[−1.07; 0.05]	1.1%
Lee et al., 2020	22	−9.86	6.13	20	−7.94	3.37	−0.38	[−0.99; 0.24]	1.0%
Lehrner et al., 2021	24	52.89	16.31	26	48.94	17.49	0.23	[−0.33; 0.79]	1.1%
Leuchter et al., 2022	8	71.13	21.85	9	80.22	21.31	−0.40	[−1.36; 0.56]	0.6%
Litz et al., 2012	13	72.33	28.63	13	53.73	26.22	0.66	[−0.14; 1.45]	0.7%
Maples−Keller et al., 2017	45	65.55	49.88	44	76.57	47.46	−0.22	[−0.64; 0.19]	1.4%
Marker et al., 2020	18	2.43	1.28	18	2.85	1.28	−0.32	[−0.98; 0.34]	0.9%
McEvoy et al., 2020	51	36.91	2.17	54	36.24	1.82	0.33	[−0.05; 0.72]	1.4%
Meyer et al., 2010	48	8.15	5.43	45	13.90	9.39	−0.75	[−1.17; −0.33]	1.4%
Meyer et al., 2022	5	2.21	3.35	5	5.62	3.47	−0.90	[−2.24; 0.43]	0.3%
Meyerbroeker et al., 2012	20	26.87	19.64	25	23.96	17.10	0.16	[−0.43; 0.75]	1.0%
Meyerbroker et al., 2018	37	36.71	15.41	17	29.18	16.45	0.47	[−0.11; 1.05]	1.0%
Mithoefer et al., 2011	12	29.30	22.52	8	66.80	22.63	−1.59	[−2.64; −0.54]	0.5%
Mithoefer et al., 2018	19	37.49	30.12	7	76.00	23.40	−1.30	[−2.25; −0.36]	0.6%
Morissette et al., 2008	4	5.15	1.05	4	4.40	1.33	0.54	[−0.89; 1.98]	0.3%
Moshier & Otto, 2017	21	23.10	10.80	13	18.60	13.30	0.37	[−0.33; 1.07]	0.9%
Nations et al., 2012	24	6.64	3.78	13	6.60	4.29	0.01	[−0.67; 0.68]	0.9%
Nejati et al., 2017	4	12.25	5.62	7	22.99	7.66	−1.39	[−2.81; 0.03]	0.3%
Nicholas et al., 2022	42	3.24	3.36	40	3.23	3.65	0.00	[−0.43; 0.44]	1.3%
Nord et al., 2019	20	12.65	6.91	19	14.37	5.81	−0.26	[−0.89; 0.37]	1.0%
Oehen et al., 2013	8	50.80	19.70	4	66.50	7.60	−0.85	[−2.12; 0.42]	0.4%
Ot'alora G et al., 2018	22	66.74	30.78	6	73.30	24.50	−0.21	[−1.12; 0.69]	0.6%
Otto et al., 2010	13	3.58	1.96	14	6.77	3.30	−1.13	[−1.95; −0.31]	0.7%
Pace−Schott et al., 2018	17	62.82	23.68	14	55.29	19.27	0.34	[−0.38; 1.05]	0.8%
Powers et al., 2009	12	15.42	13.39	12	27.08	21.37	−0.63	[−1.45; 0.19]	0.7%
Pyrkosch et al., 2018	36	21.16	8.81	33	19.79	9.89	0.15	[−0.33; 0.62]	1.3%
Raeder et al., 2019	20	26.05	14.35	23	32.17	19.72	−0.34	[−0.95; 0.26]	1.0%
Reinecke et al., 2020	17	2.10	17.20	14	21.30	30.80	−0.77	[−1.51; −0.03]	0.8%
Reitmaier et al., 2022	27	64.45	19.13	26	56.46	24.76	0.36	[−0.19; 0.90]	1.1%
Rodebaugh et al., 2013	18	55.89	20.46	16	65.85	20.19	−0.48	[−1.16; 0.21]	0.9%
Ross et al., 2016	12	7.45	4.81	14	13.88	5.19	−1.24	[−2.09; −0.39]	0.7%
Ross et al., 2021	6	40.33	10.97	5	49.01	17.87	−0.55	[−1.77; 0.67]	0.4%
Rubin et al., 2022	10	123.50	21.21	8	119.63	19.54	0.18	[−0.75; 1.11]	0.6%
Santa Ana et al., 2015	23	0.76	1.37	24	1.42	1.89	−0.39	[−0.97; 0.19]	1.1%
Sherman et al., 2017	8	1.05	1.65	7	0.79	1.39	0.16	[−0.86; 1.18]	0.5%
Shiban et al., 2017	15	1.06	0.63	14	1.29	0.84	−0.30	[−1.04; 0.43]	0.8%
Siegmund et al., 2011	20	11.85	6.05	19	17.26	2.28	−1.15	[−1.83; −0.46]	0.9%
Simpson et al., 2010	15	11.90	2.35	15	13.75	3.60	−0.59	[−1.33; 0.14]	0.8%
Sippel et al., 2020	6	20.50	19.53	7	29.71	26.39	−0.36	[−1.47; 0.74]	0.5%
Smits et al., 2006	35	58.48	24.33	33	58.97	23.01	−0.02	[−0.50; 0.46]	1.3%
Smits et al., 2014	20	38.78	10.24	20	47.19	10.49	−0.80	[−1.44; −0.15]	0.9%
Smits et al., 2020a	114	60.56	23.34	38	65.88	25.16	−0.22	[−0.59; 0.15]	1.5%
Smits et al., 2020b	27	15.37	10.38	26	17.80	13.77	−0.20	[−0.74; 0.34]	1.1%
Soravia et al., 2014	11	43.30	11.28	11	53.50	17.58	−0.66	[−1.53; 0.20]	0.7%
Soucy et al., 2021	203	6.26	4.78	231	6.22	5.05	0.01	[−0.18; 0.20]	1.9%
Steudte−Schmiedgen et al., 2021	17	14.17	2.39	16	15.95	3.92	−0.54	[−1.24; 0.16]	0.9%
Storch et al., 2007	12	10.10	6.80	12	8.60	8.80	0.18	[−0.62; 0.99]	0.7%
Tart et al., 2013	15	29.73	25.67	14	35.55	25.18	−0.22	[−0.95; 0.51]	0.8%
Thierree et al., 2021	13	41.80	31.90	16	51.60	23.70	−0.34	[−1.08; 0.39]	0.8%
Tuerk et al., 2018	14	71.01	14.37	12	75.08	10.77	−0.31	[−1.08; 0.47]	0.8%
Vermes et al., 2020	21	0.36	0.60	21	0.51	0.44	−0.28	[−0.89; 0.33]	1.0%
Weingarden et al., 2019	12	19.64	6.80	14	18.77	10.03	0.10	[−0.67; 0.87]	0.8%
Wilhelm et al., 2008	10	10.20	7.20	13	14.50	6.40	−0.61	[−1.46; 0.23]	0.7%
Witkiewitz et al., 2019	47	5.12	3.14	37	5.64	4.06	−0.14	[−0.58; 0.29]	1.3%
Wolfson et al., 2020	13	38.90	10.60	5	48.60	12.60	−0.83	[−1.91; 0.25]	0.5%
Zoellner et al., 2017	15	17.10	10.57	16	14.67	9.27	0.24	[−0.47; 0.95]	0.8%
**Random effects model**	**2979**			**2797**			**0.26**	**[0.35; 0.17]**	**100.0%**
**Prediction interval**								**[0.87; 0.35]**	

Heterogeneity: I^2 = 54%, $\square^2$ = 0.0921, $p < 0.01$

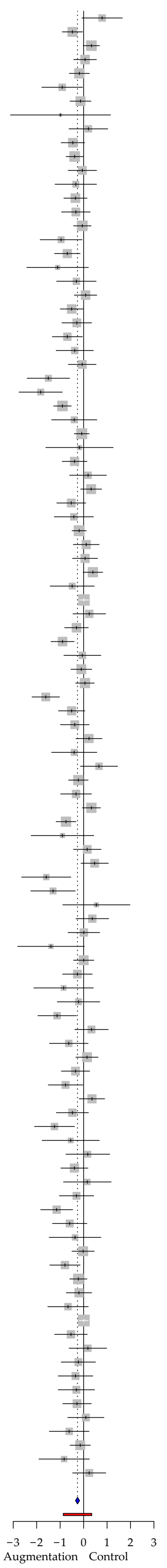

2.7. Analyses stratified by HiTOP dimensions (excludes five studies)

Supplemental Figure S9. Sub-study Random effects meta-analysis results for the HiTOP dimensions of (A) Fear, (B) Thought disorder, (C) Distress and (D) Substance abuse.

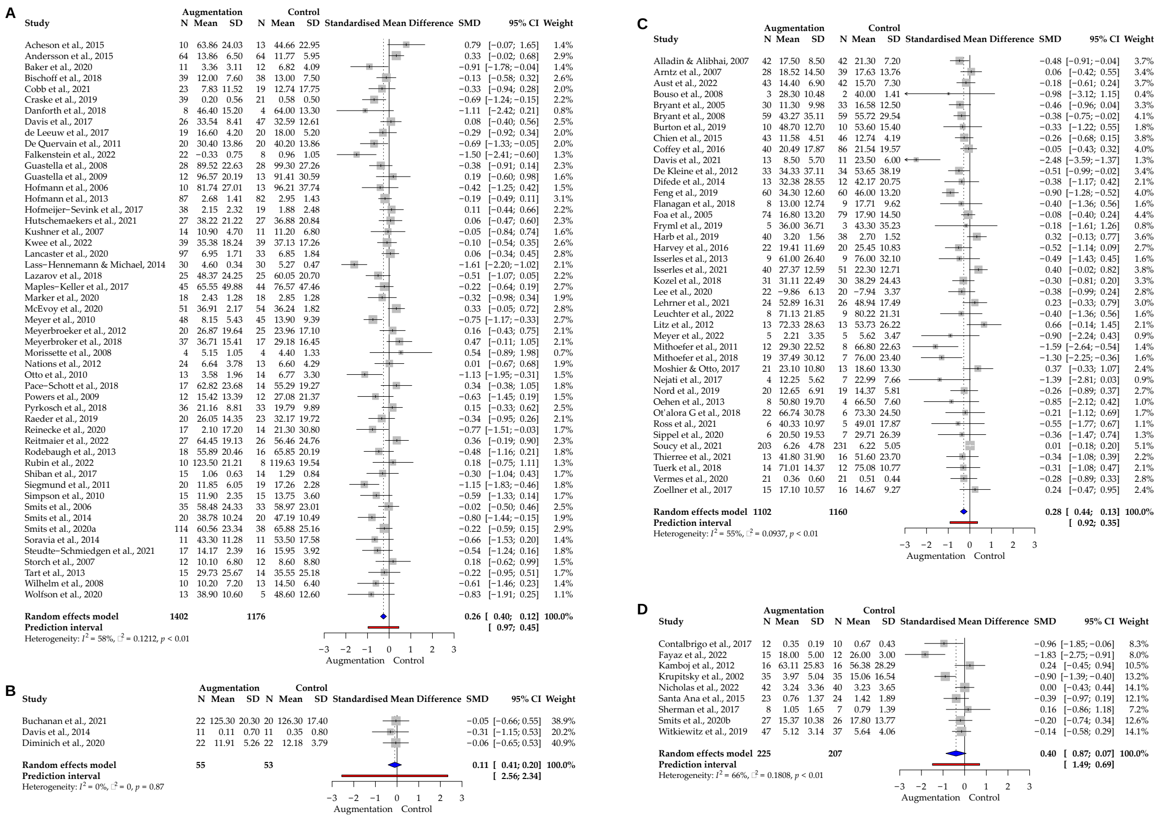

Supplementary Table S5. Main effects for each of the HiTOP dimensions (excluding five studies[15,23,28,53,104] which did not fit any of them) from a random effects meta-analysis

HiTOP dimension	No. studies	Hedges' *g*	95% CI	*p*
Fear	51	-0.26	(-0.40, -0.12)	0.0004
Distress	40	-0.28	(-0.44, -0.13)	0.0008
Substance abuse	9	-0.40	(-0.87, 0.065)	0.083
Thought disorder	3	-0.11	(-0.41, 0.20)	0.28

2.8. Analyses stratified by specific augmentation (> 3 studies per category; excludes 22 studies)

Supplemental Figure S10. Sub-study random effects meta-analysis results for augmentation categories including more than three studies. These were (A) memory (e.g., memory support; trauma retrieval), (B) imagery (e.g., imagery rescripting; imagery-enhanced CBT), (C) cortisol, (D) D-cycloserine, (E) motivational (e.g., motivational interviewing), (F) brain stimulation (e.g., rTMS, tDCS), (G) psychotropic (e.g., MDMA, psilocybin, ketamine, or cannabidiol), (H) oxytocin, (I) yohimbine, and (J) bias modification (e.g., cognitive bias or attentional bias modifications) augmentations.

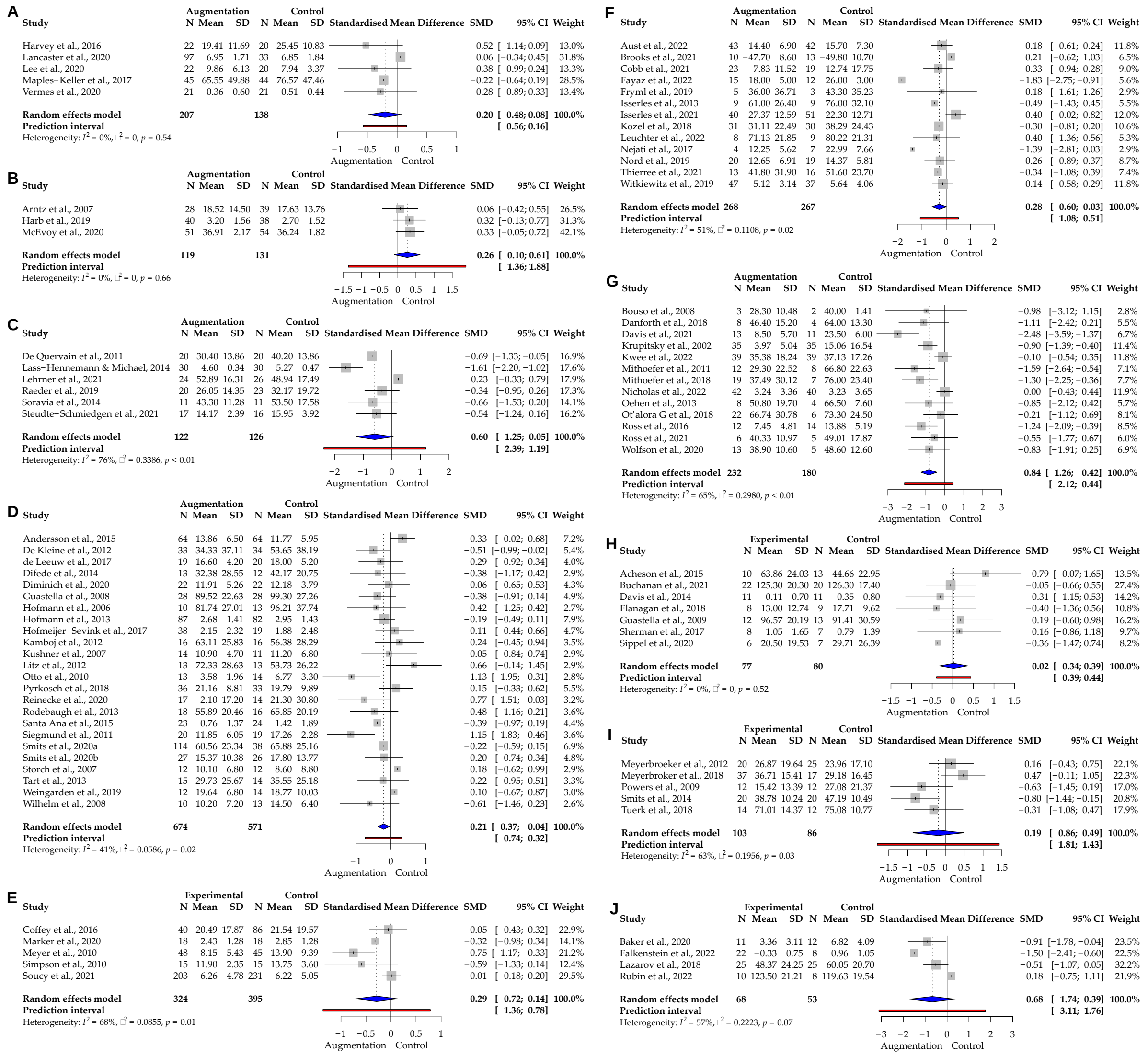

Supplementary Table S6. Main effects for each of the specific augmentation categories (excluding 22 studies which did not fit into groups larger than three) from a random effects meta-analysis

Augmentation type	No. studies	Hedges' *g*	95% CI	*p*
Memory	5	-0.20	(-0.48, 0.076)	0.11
Imagery	3	0.26	(-0.10, 0.61)	0.09
Cortisol	6	-0.60	(-1.25, 0.048)	0.06
D-cycloserine	24	-0.21	(-0.37, -0.037)	0.02
Brain stimulation	13	-0.28	(-0.60, 0.030)	0.07
Psychotropic	13	-0.84	(-1.26, -0.42)	0.0009
Psychotropic (excluding waitlist control[3])	12	-0.69	(-1.04, -0.34)	0.001
Oxytocin	7	0.23	(-0.34, 0.39)	0.89
Yohimbine	5	-0.19	(-0.86, 0.49)	0.48
Motivational	5	-0.29	(-0.72, 0.14)	0.14
Bias modifications	4	-0.68	(-1.74, 0.39)	0.14

References

1. Acheson, D. T., Feifel, D., Kamenski, M., Mckinney, R. & Risbrough, V. B. Intranasal oxytocin administration prior to exposure therapy for arachnophobia impedes treatment response. *Depression and Anxiety* **32**, 400–407 (2015).

2. Andersson, E. *et al.* D-cycloserine vs placebo as adjunct to cognitive behavioral therapy for obsessive-compulsive disorder and interaction with antidepressants: a randomized clinical trial. *JAMA Psychiatry* **72**, 659–667 (2015).

3. Davis, A. K. *et al.* Effects of psilocybin-assisted therapy on major depressive disorder: a randomized clinical trial. *JAMA Psychiatry* **78**, 481–489 (2021).

4. Falkenstein, M. J. *et al.* Feasibility and Acceptability of Cognitive Bias Modification for Interpretation as an Adjunctive Treatment for OCD and Related Disorders: A Pilot Randomized Controlled Trial. *Behavior Therapy* **53**, 294–309 (2022).

5. Fayaz Feyzi, Y., Vahed, N., Sadeghamal Nikraftar, N. & Arezoomandan, R. Synergistic effect of combined transcranial direct current stimulation and Matrix Model on the reduction of methamphetamine craving and improvement of cognitive functioning: a randomized sham-controlled study. *The American Journal of Drug and Alcohol Abuse* **48**, 311–320 (2022).

6. Feng, B. *et al.* Transcutaneous electrical acupoint stimulation for post-traumatic stress disorder: Assessor-blinded, randomized controlled study. *Psychiatry and clinical neurosciences* **73**, 179–186 (2019).

7. Harb, G. C. *et al.* Randomized controlled trial of imagery rehearsal for posttraumatic nightmares in combat veterans. *Journal of Clinical Sleep Medicine* **15**, 757–767 (2019).

8. Isserles, M. *et al.* Deep Transcranial Magnetic Stimulation Combined With Brief Exposure for Posttraumatic Stress Disorder: A Prospective Multisite Randomized Trial. *Biological Psychiatry* **90**, 721–728 (2021).

9. Krupitsky, E. *et al.* Ketamine psychotherapy for heroin addiction: immediate effects and two-year follow-up. *Journal of Substance Abuse Treatment* **23**, 273–283 (2002).

10. Lass-Hennemann, J. & Michael, T. Endogenous cortisol levels influence exposure therapy in spider phobia. *Behaviour Research and Therapy* **60**, 39–45 (2014).

11. Litz, B. T. *et al.* A randomized placebo-controlled trial of D-cycloserine and exposure therapy for posttraumatic stress disorder. *Journal of Psychiatric Research* **46**, 1184–1190 (2012).

12. McEvoy, P. M. *et al.* Imagery-enhanced v. verbally-based group cognitive behavior therapy for social anxiety disorder: a randomized clinical trial. *Psychological Medicine* 1–10 (2020).

13. Meyerbröker, K., Morina, N. & Emmelkamp, P. Enhancement of exposure therapy in participants with specific phobia: a randomized controlled trial comparing yohimbine, propranolol and placebo. *Journal of Anxiety Disorders* **57**, 48–56 (2018).

14. Mithoefer, M. C., Wagner, M. T., Mithoefer, A. T., Jerome, L. & Doblin, R. The safety and efficacy of 3, 4-methylenedioxymethamphetamine-assisted psychotherapy in

subjects with chronic, treatment-resistant posttraumatic stress disorder: the first randomized controlled pilot study. *Journal of Psychopharmacology* **25**, 439–452 (2011).

15. Ross, S. *et al.* Rapid and sustained symptom reduction following psilocybin treatment for anxiety and depression in patients with life-threatening cancer: a randomized controlled trial. *Journal of Psychopharmacology* **30**, 1165–1180 (2016).
16. Siegmund, A. *et al.* D-cycloserine does not improve but might slightly speed up the outcome of in-vivo exposure therapy in patients with severe agoraphobia and panic disorder in a randomized double blind clinical trial. *Journal of Psychiatric Research* **45**, 1042–1047 (2011).
17. Alladin, A. & Alibhai, A. Cognitive hypnotherapy for depression: An empirical investigation. *International Journal of Clinical and Experimental Hypnosis* **55**, 147–166 (2007).
18. Arntz, A., Tiesema, M. & Kindt, M. Treatment of PTSD: A comparison of imaginal exposure with and without imagery rescripting. *Journal of Behavior Therapy and Experimental Psychiatry* **38**, 345–370 (2007).
19. Aust, S. *et al.* Efficacy of Augmentation of Cognitive Behavioral Therapy With Transcranial Direct Current Stimulation for Depression: A Randomized Clinical Trial. *JAMA Psychiatry* **79**, 528–537 (2022).
20. Baker, A. *et al.* A pilot study augmenting cognitive behavioral therapy for panic disorder with attention bias modification: Clinical and psychophysiological outcomes. *Journal of Behavior Therapy and Experimental Psychiatry* **68**, 101568 (2020).
21. Bischoff, S. *et al.* Running for extinction? Aerobic exercise as an augmentation of exposure therapy in panic disorder with agoraphobia. *Journal of Psychiatric Research* **101**, 34–41 (2018).
22. Bouso, J. C., Doblin, R., Farré, M., Alcázar, M. Á. & Gómez-Jarabo, G. MDMA-assisted psychotherapy using low doses in a small sample of women with chronic posttraumatic stress disorder. *Journal of Psychoactive Drugs* **40**, 225–236 (2008).
23. Brooks, H. *et al.* Enhancing Cognition in Older Persons with Depression or Anxiety with a Combination of Mindfulness-Based Stress Reduction (MBSR) and Transcranial Direct Current Stimulation (tDCS): Results of a Pilot Randomized Clinical Trial. *Mindfulness* **12**, 3047–3059 (2021).
24. Bryant, R. A., Moulds, M. L., Guthrie, R. M. & Nixon, R. D. The additive benefit of hypnosis and cognitive-behavioral therapy in treating acute stress disorder. *Journal of Consulting and Clinical Psychology* **73**, 334 (2005).
25. Bryant, R. A. *et al.* A randomized controlled trial of exposure therapy and cognitive restructuring for posttraumatic stress disorder. *Journal of Consulting and Clinical Psychology* **76**, 695 (2008).
26. Buchanan, R. W. *et al.* Combined Oxytocin and Cognitive Behavioral Social Skills Training for Social Function in People With Schizophrenia. *Journal of Clinical Psychopharmacology* **41**, 236–243 (2021).

27. Burton, L. E., Qeadan, F. & Burge, M. R. Efficacy of equine-assisted psychotherapy in veterans with posttraumatic stress disorder. *Journal of Integrative Medicine* **17**, 14–19 (2019).

28. Cesa, G. L. *et al.* Virtual reality for enhancing the cognitive behavioral treatment of obesity with binge eating disorder: randomized controlled study with one-year follow-up. *Journal of Medical Internet Research* **15**, e2441 (2013).

29. Chien, H., Chung, Y., Yeh, M. & Lee, J. Breathing exercise combined with cognitive behavioural intervention improves sleep quality and heart rate variability in major depression. *Journal of Clinical Nursing* **24**, 3206–3214 (2015).

30. Cobb, A. R. *et al.* tDCS-Augmented in vivo exposure therapy for specific fears: A randomized clinical trial. *Journal of Anxiety Disorders* **78**, 102344 (2021).

31. Coffey, S. F. *et al.* Trauma-focused exposure therapy for chronic posttraumatic stress disorder in alcohol and drug dependent patients: A randomized controlled trial. *Psychology of Addictive Behaviors* **30**, 778 (2016).

32. Contalbrigo, L. *et al.* The efficacy of dog assisted therapy in detained drug users: A pilot study in an Italian attenuated custody institute. *International Journal of Environmental Research and Public Health* **14**, 683 (2017).

33. Craske, M. G., Fanselow, M., Treanor, M. & Bystritksy, A. Cholinergic modulation of exposure disrupts hippocampal processes and augments extinction: Proof-of-concept study with social anxiety disorder. *Biological Psychiatry* **86**, 703–711 (2019).

34. Danforth, A. L. *et al.* Reduction in social anxiety after MDMA-assisted psychotherapy with autistic adults: a randomized, double-blind, placebo-controlled pilot study. *Psychopharmacology* **235**, 3137–3148 (2018).

35. Davis, M. C. *et al.* Oxytocin-augmented social cognitive skills training in schizophrenia. *Neuropsychopharmacology* **39**, 2070–2077 (2014).

36. Davis, M. L. *et al.* A randomized controlled study of power posing before public speaking exposure for social anxiety disorder: No evidence for augmentative effects. *Journal of Anxiety Disorders* **52**, 1–7 (2017).

37. de Kleine, R. A., Hendriks, G.-J., Kusters, W. J., Broekman, T. G. & van Minnen, A. A randomized placebo-controlled trial of D-cycloserine to enhance exposure therapy for posttraumatic stress disorder. *Biological Psychiatry* **71**, 962–968 (2012).

38. De Leeuw, A., van Megen, H., Kahn, R. & Westenberg, H. D-cycloserine addition to exposure sessions in the treatment of patients with obsessive-compulsive disorder. *European Psychiatry* **40**, 38–44 (2017).

39. de Quervain, J.-F., Dominique JF *et al.* Glucocorticoids enhance extinction-based psychotherapy. *Proceedings of the National Academy of Sciences* **108**, 6621–6625 (2011).

40. Difede, J. *et al.* D-cycloserine augmentation of exposure therapy for post-traumatic stress disorder: a pilot randomized clinical trial. *Neuropsychopharmacology* **39**, 1052–1058 (2014).

41. Diminich, E. D. *et al.* D-cycloserine augmentation of cognitive behavioral therapy for delusions: A randomized clinical trial. *Schizophrenia Research* **222**, 145–152 (2020).

42. Flanagan, J. C., Sippel, L. M., Wahlquist, A., Moran-Santa Maria, M. M. & Back, S. E. Augmenting prolonged exposure therapy for PTSD with intranasal oxytocin: a randomized, placebo-controlled pilot trial. *Journal of Psychiatric Research* **98**, 64–69 (2018).

43. Foa, E. B. *et al.* Randomized trial of prolonged exposure for posttraumatic stress disorder with and without cognitive restructuring: outcome at academic and community clinics. *Journal of Consulting and Clinical Psychology* **73**, 953 (2005).

44. Fryml, L. D. *et al.* Exposure therapy and simultaneous repetitive transcranial magnetic stimulation: a controlled pilot trial for the treatment of posttraumatic stress disorder. *The Journal of ECT* **35**, 53–60 (2019).

45. Guastella, A. J. *et al.* A randomized controlled trial of D-cycloserine enhancement of exposure therapy for social anxiety disorder. *Biological Psychiatry* **63**, 544–549 (2008).

46. Guastella, A. J., Howard, A. L., Dadds, M. R., Mitchell, P. & Carson, D. S. A randomized controlled trial of intranasal oxytocin as an adjunct to exposure therapy for social anxiety disorder. *Psychoneuroendocrinology* **34**, 917–923 (2009).

47. Harvey, A. G. *et al.* Improving outcome for mental disorders by enhancing memory for treatment. *Behaviour Research and Therapy* **81**, 35–46 (2016).

48. Hofmann, S. G. *et al.* Augmentation of exposure therapy with D-cycloserine for social anxiety disorder. *Archives of General Psychiatry* **63**, 298–304 (2006).

49. Hofmann, S. G. *et al.* D-Cycloserine as an augmentation strategy with cognitive-behavioral therapy for social anxiety disorder. *American Journal of Psychiatry* **170**, 751–758 (2013).

50. Hofmeijer-Sevink, M. K. *et al.* No effects of D-cycloserine enhancement in exposure with response prevention therapy in panic disorder with agoraphobia: a double-blind, randomized controlled trial. *Journal of Clinical Psychopharmacology* **37**, 531–539 (2017).

51. Hutschemaekers, M. H. M., de Kleine, R. A., Hendriks, G.-J., Kampman, M. & Roelofs, K. The enhancing effects of testosterone in exposure treatment for social anxiety disorder: a randomized proof-of-concept trial. *Transl Psychiatry* **11**, 1–7 (2021).

52. Isserles, M. *et al.* Effectiveness of deep transcranial magnetic stimulation combined with a brief exposure procedure in post-traumatic stress disorder–a pilot study. *Brain Stimulation* **6**, 377–383 (2013).

53. Janse, P. D. *et al.* The effect of feedback-informed cognitive behavioral therapy on treatment outcome: A randomized controlled trial. *Journal of Consulting and Clinical Psychology* **88**, 818–828 (2020).

54. Kamboj, S. K. *et al.* Cue exposure and response prevention with heavy smokers: a laboratory-based randomised placebo-controlled trial examining the effects of D-cycloserine on cue reactivity and attentional bias. *Psychopharmacology* **221**, 273–284 (2012).

55. Kozel, F. A. *et al.* Repetitive TMS to augment cognitive processing therapy in combat veterans of recent conflicts with PTSD: a randomized clinical trial. *Journal of Affective Disorders* **229**, 506–514 (2018).

56. Kushner, M. G. *et al.* D-cycloserine augmented exposure therapy for obsessive-compulsive disorder. *Biological Psychiatry* **62**, 835–838 (2007).

57. Kwee, C. M. *et al.* Cannabidiol enhancement of exposure therapy in treatment refractory patients with social anxiety disorder and panic disorder with agoraphobia: A randomised controlled trial. *European Neuropsychopharmacology* **59**, 58–67 (2022).

58. Lancaster, C. L., Monfils, M.-H. & Telch, M. J. Augmenting exposure therapy with pre-extinction fear memory reactivation and deepened extinction: A randomized controlled trial. *Behaviour Research and Therapy* **135**, 103730 (2020).

59. Lazarov, A. *et al.* Attention bias modification augments cognitive–behavioral group therapy for social anxiety disorder: a randomized controlled trial. *Psychological Medicine* **48**, 2177–2185 (2018).

60. Lee, J. Y., Dong, L., Gumport, N. B. & Harvey, A. G. Establishing the dose of memory support to improve patient memory for treatment and treatment outcome. *Journal of Behavior Therapy and Experimental Psychiatry* **68**, 101526 (2020).

61. Lehrner, A. *et al.* A randomized, double-blind, placebo-controlled trial of hydrocortisone augmentation of Prolonged Exposure for PTSD in U.S. combat veterans. *Behaviour Research and Therapy* **144**, 103924 (2021).

62. Leuchter, M. K. *et al.* Treatment of Spider Phobia Using Repeated Exposures and Adjunctive Repetitive Transcranial Magnetic Stimulation: A Proof-of-Concept Study. *Frontiers in Psychiatry* **13**, (2022).

63. Maples-Keller, J. L. *et al.* Targeting memory reconsolidation to prevent the return of fear in patients with fear of flying. *Depression and Anxiety* **34**, 610–620 (2017).

64. Marker, I., Corbett, B. E., Drummond, S. P. A. & Norton, P. J. Intermittent Motivational Interviewing and Transdiagnostic CBT for Anxiety: A Randomized Controlled Trial. *Journal of Anxiety Disorders* **75**, 102276 (2020).

65. Meyer, E. *et al.* A randomized clinical trial to examine enhancing cognitive-behavioral group therapy for obsessive-compulsive disorder with motivational interviewing and thought mapping. *Behavioural and Cognitive Psychotherapy* **38**, 319–336 (2010).

66. Meyer, J. D. *et al.* Feasibility of an Exercise and CBT Intervention for Treatment of Depression: A Pilot Randomized Controlled Trial. *Front Psychiatry* **13**, 799600 (2022).

67. Meyerbroeker, K., Powers, M. B., Van Stegeren, A. & Emmelkamp, P. M. Does yohimbine hydrochloride facilitate fear extinction in virtual reality treatment of fear of flying? A randomized placebo-controlled trial. *Psychotherapy and Psychosomatics* **81**, 29–37 (2012).

68. Mithoefer, M. C. *et al.* 3, 4-methylenedioxymethamphetamine (MDMA)-assisted psychotherapy for post-traumatic stress disorder in military veterans, firefighters, and police officers: a randomised, double-blind, dose-response, phase 2 clinical trial. *The Lancet Psychiatry* **5**, 486–497 (2018).

69. Morissette, S. B., Spiegel, D. A. & Barlow, D. H. Combining exposure and pharmacotherapy in the treatment of social anxiety disorder: A preliminary study of

state dependent learning. *Journal of Psychopathology and Behavioral Assessment* **30**, 211–219 (2008).

70. Moshier, S. J. & Otto, M. W. Behavioral activation treatment for major depression: A randomized trial of the efficacy of augmentation with cognitive control training. *Journal of Affective Disorders* **210**, 265–268 (2017).

71. Nations, K. R. *et al.* Evaluation of the glycine transporter inhibitor Org 25935 as augmentation to cognitive-behavioral therapy for panic disorder: a multicenter, randomized, double-blind, placebo-controlled trial. *The Journal of Clinical Psychiatry* **73**, 6751 (2012).

72. Nejati, V., Salehinejad, M. A., Shahidi, N. & Abedin, A. Psychological intervention combined with direct electrical brain stimulation (PIN-CODES) for treating major depression: A pre-test, post-test, follow-up pilot study. *Neurology, Psychiatry and Brain Research* **25**, 15–23 (2017).

73. Nicholas, C. R. *et al.* The effects of MDMA-assisted therapy on alcohol and substance use in a phase 3 trial for treatment of severe PTSD. *Drug and Alcohol Dependence* **233**, 109356 (2022).

74. Nord, C. L. *et al.* Neural predictors of treatment response to brain stimulation and psychological therapy in depression: a double-blind randomized controlled trial. *Neuropsychopharmacol.* **44**, 1613–1622 (2019).

75. Oehen, P., Traber, R., Widmer, V. & Schnyder, U. A randomized, controlled pilot study of MDMA (3, 4-Methylenedioxymethamphetamine)-assisted psychotherapy for treatment of resistant, chronic Post-Traumatic Stress Disorder (PTSD). *Journal of Psychopharmacology* **27**, 40–52 (2013).

76. Ot’alora G, M. *et al.* 3, 4-Methylenedioxymethamphetamine-assisted psychotherapy for treatment of chronic posttraumatic stress disorder: a randomized phase 2 controlled trial. *Journal of Psychopharmacology* **32**, 1295–1307 (2018).

77. Otto, M. W. *et al.* Efficacy of d-cycloserine for enhancing response to cognitive-behavior therapy for panic disorder. *Biological Psychiatry* **67**, 365–370 (2010).

78. Pace-Schott, E. F. *et al.* Effects of post-exposure naps on exposure therapy for social anxiety. *Psychiatry Research* **270**, 523–530 (2018).

79. Powers, M. B., Smits, J. A., Otto, M. W., Sanders, C. & Emmelkamp, P. M. Facilitation of fear extinction in phobic participants with a novel cognitive enhancer: a randomized placebo controlled trial of yohimbine augmentation. *Journal of Anxiety Disorders* **23**, 350–356 (2009).

80. Pyrkosch, L. *et al.* Learn to forget: Does post-exposure administration of d-cycloserine enhance fear extinction in agoraphobia? *Journal of Psychiatric Research* **105**, 153–163 (2018).

81. Raeder, F. *et al.* Post-exposure cortisol administration does not augment the success of exposure therapy: A randomized placebo-controlled study. *Psychoneuroendocrinology* **99**, 174–182 (2019).

82. Reinecke, A., Nickless, A., Browning, M. & Harmer, C. J. Neurocognitive processes in d-cycloserine augmented single-session exposure therapy for anxiety: A randomized placebo-controlled trial. *Behaviour Research and Therapy* **129**, 103607 (2020).

83. Reitmaier, J. *et al.* Effects of rhythmic eye movements during a virtual reality exposure paradigm for spider-phobic patients. *Psychology and Psychotherapy: Theory, Research and Practice* **95**, 57–78 (2022).

84. Rodebaugh, T. L., Levinson, C. A. & Lenze, E. J. A high-throughput clinical assay for testing drug facilitation of exposure therapy. *Depression and Anxiety* **30**, 631–637 (2013).

85. Ross, S. *et al.* Acute and Sustained Reductions in Loss of Meaning and Suicidal Ideation Following Psilocybin-Assisted Psychotherapy for Psychiatric and Existential Distress in Life-Threatening Cancer. *ACS Pharmacol Transl Sci* **4**, 553–562 (2021).

86. Rubin, M., Muller, K., Hayhoe, M. M. & Telch, M. J. Attention guidance augmentation of virtual reality exposure therapy for social anxiety disorder: a pilot randomized controlled trial. *Cognitive Behaviour Therapy* **0**, 1–17 (2022).

87. Santa Ana, E. J. *et al.* D-cycloserine combined with cue exposure therapy fails to attenuate subjective and physiological craving in cocaine dependence. *The American Journal on Addictions* **24**, 217–224 (2015).

88. Sherman, B. J., Baker, N. L. & McRae-Clark, A. L. Effect of oxytocin pretreatment on cannabis outcomes in a brief motivational intervention. *Psychiatry Research* **249**, 318–320 (2017).

89. Shiban, Y. *et al.* Diaphragmatic breathing during virtual reality exposure therapy for aviophobia: functional coping strategy or avoidance behavior? a pilot study. *BMC Psychiatry* **17**, 1–10 (2017).

90. Simpson, H. B. *et al.* Challenges using motivational interviewing as an adjunct to exposure therapy for obsessive-compulsive disorder. *Behaviour Research and Therapy* **48**, 941–948 (2010).

91. Sippel, L. M., King, C. E., Wahlquist, A. E. & Flanagan, J. C. A Preliminary Examination of Endogenous Peripheral Oxytocin in a Pilot Randomized Clinical Trial of Oxytocin-Enhanced Psychotherapy for Posttraumatic Stress Disorder. *J Clin Psychopharmacol* **40**, 401–404 (2020).

92. Smits, J. A., Powers, M. B., Buxkamper, R. & Telch, M. J. The efficacy of videotape feedback for enhancing the effects of exposure-based treatment for social anxiety disorder: a controlled investigation. *Behaviour Research and Therapy* **44**, 1773–1785 (2006).

93. Smits, J. A. *et al.* Yohimbine enhancement of exposure therapy for social anxiety disorder: a randomized controlled trial. *Biological Psychiatry* **75**, 840–846 (2014).

94. Smits, J. A. J. *et al.* Dose timing of D-cycloserine to augment exposure therapy for social anxiety disorder: A randomized clinical trial. *JAMA Network Open* **3**, e206777–e206777 (2020).

95. Smits, J. A. J. *et al.* Enhancing panic and smoking reduction treatment with D-Cycloserine: A pilot randomized clinical trial. *Drug and Alcohol Dependence* **208**, 107877 (2020).

96. Soravia, L. M. *et al.* Glucocorticoids enhance in vivo exposure-based therapy of spider phobia. *Depression and Anxiety* **31**, 429–435 (2014).

97. Soucy, J. N., Hadjistavropoulos, H. D., Karin, E., Dear, B. F. & Titov, N. Brief online motivational interviewing pre-treatment intervention for enhancing internet-delivered cognitive behaviour therapy: A randomized controlled trial. *Internet Interventions* **25**, 100394 (2021).

98. Steudte-Schmiedgen, S., Fay, E., Capitao, L., Kirschbaum, C. & Reinecke, A. Hydrocortisone as an adjunct to brief cognitive-behavioural therapy for specific fear: Endocrine and cognitive biomarkers as predictors of symptom improvement. *J Psychopharmacol* **35**, 641–651 (2021).

99. Storch, E. A. *et al.* D-cycloserine does not enhance exposure–response prevention therapy in obsessive–compulsive disorder. *International Clinical Psychopharmacology* **22**, 230–237 (2007).

100. Tart, C. D. *et al.* Augmentation of exposure therapy with post-session administration of D-cycloserine. *Journal of Psychiatric Research* **47**, 168–174 (2013).

101. Thierrée, S. *et al.* Combining Trauma Script Exposure With rTMS to Reduce Symptoms of Post-Traumatic Stress Disorder: Randomized Controlled Trial. *Neuromodulation: Technology at the Neural Interface* **25**, 549–557 (2022).

102. Tuerk, P. W. *et al.* Augmenting treatment efficiency in exposure therapy for PTSD: a randomized double-blind placebo-controlled trial of yohimbine HCl. *Cognitive Behaviour Therapy* **47**, 351–371 (2018).

103. Vermes, J. S. *et al.* Targeting the reconsolidation of traumatic memories with a brief 2-session imaginal exposure intervention in post-traumatic stress disorder. *Journal of Affective Disorders* **276**, 487–494 (2020).

104. Weingarden, H. *et al.* D-cycloserine-augmented behavior therapy for body dysmorphic disorder: a preliminary efficacy trial. *Cognitive Therapy and Research* **43**, 937–947 (2019).

105. Wilhelm, S. *et al.* Augmentation of behavior therapy with D-cycloserine for obsessive-compulsive disorder. *American Journal of Psychiatry* **165**, 335–341 (2008).

106. Witkiewitz, K. *et al.* Mindfulness-based relapse prevention and transcranial direct current stimulation to reduce heavy drinking: A double-blind sham-controlled randomized trial. *Alcoholism: Clinical and Experimental Research* **43**, 1296–1307 (2019).

107. Wolfson, P. E. *et al.* MDMA-assisted psychotherapy for treatment of anxiety and other psychological distress related to life-threatening illnesses: a randomized pilot study. *Scientific Reports* **10**, 1–15 (2020).

108. Zoellner, L. A. *et al.* Enhancing extinction learning in posttraumatic stress disorder with brief daily imaginal exposure and methylene blue: a randomized controlled trial. *The Journal of Clinical Psychiatry* **78**, 0–0 (2017).
